# Twisted-Planar-Twisted expanded porphyrinoid dimer as a rudimentary reaction-based methanol indicator

**DOI:** 10.1038/s41467-020-19118-9

**Published:** 2020-10-20

**Authors:** Qizhao Li, Chengjie Li, Glib Baryshnikov, Yubin Ding, Chengxi Zhao, Tingting Gu, Feng Sha, Xu Liang, Weihua Zhu, Xinyan Wu, Hans Ågren, Jonathan L. Sessler, Yongshu Xie

**Affiliations:** 1 Key Laboratory for Advanced Materials and Joint International Research Laboratory of Precision Chemistry and Molecular Engineering, Feringa Nobel Prize Scientist Joint Research Center, Frontiers Science Center for Materiobiology and Dynamic Chemistry, School of Chemistry and Molecular Engineering, East China University of Science & Technology, 130 Meilong Road, 200237 Shanghai, China; 2 School of Biotechnology, KTH Royal Institute of Technology, SE-10691 Stockholm, Sweden; 3 School of Chemistry and Chemical Engineering, Jiangsu University, 212013 Zhenjiang, China; 4 Department of Chemistry, The University of Texas at Austin, Austin, TX 78712-1224 USA

**Keywords:** Sensors, Synthetic chemistry methodology, Self-assembly

## Abstract

Directly linked porphyrin dimers have attracted considerable attention because of their intriguing electronic features. Most emphasis has been placed on either dimers with large dihedral angles between the constituent planar monomeric subunits or those with overall planarity, referred to as “Planar-Twisted-Planar” and “Planar-Planar-Planar”, respectively. Herein, we report a “Twisted-Planar-Twisted” framework, the hexaphyrin dimer **D** that exists in a *trans* configuration. Treatment of **D** with MeOH affords two isomeric dimers, **MD1** and **MD2**, both of which incorporate a methoxy moiety and exist in *cis* orientations with respect to the tethering linkage. The methanol-promoted conversion is accompanied by a readily discernible color change from green to brown and is not induced to an appreciable level by other alcohols. Dimer **D** thus acts as a rudimentary, albeit highly selective, reaction-based methanol indicator. This work provides a promising approach for constructing reaction-based chemosensors using porphyrinoid dimers of nonplanar subunits with biased reactivity.

## Abstract (web summary)

Directly linked porphyrin dimers show intriguing electronic features but emphasis has been placed on planar monomeric units. Here, the authors report a Twisted-Planar-Twisted framework which can undergo a cis-trans transformation accompanied by a colour change in presence of methanol, making this framework applicable as a methanol sensor.

## Introduction

**Fig. 1:**
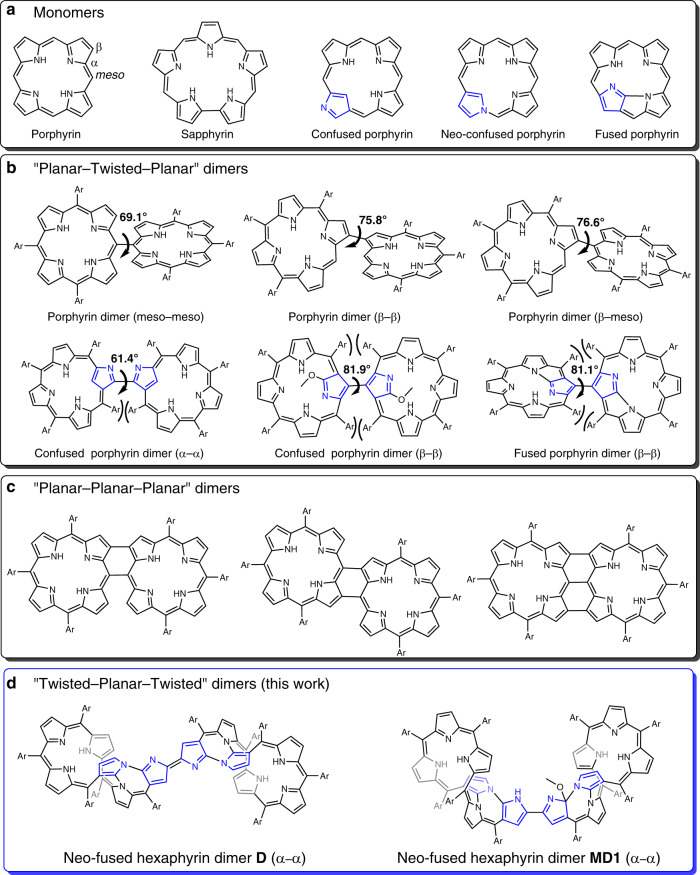
Chemical structures of porphyrin, porphyrin analogs, and their dimers. **a** Porphyrin and related monomeric macrocycles; **b** examples of “Planar–Twisted–Planar” dimers: the two planar porphyrin/porphyrinoid units are linked in a highly twisted manner; **c** examples of “Planar-Planar-Planar” dimers: the two planar porphyrin units are linked by means of direct fusion; **d** “Twisted–Planar–Twisted” dimer **D** reported in this work: Note that the twisted hexaphyrin units are linked through a coplanar bipyrrolic linkage. Ar represents aromatic substituents. The blue-colored pyrrolic units represent confused pyrroles, which are linked with adjacent pyrroles or meso-carbons through C_α_-C_β_ or N-C_β_ atoms.

Naturally occurring tetrapyrrolic pigments, including porphyrin (Fig. 1a), play essential roles in both aquatic and terrestrial life, while various synthetic analogs have seen extensive application in a wide range of application areas, including in materials and medicinal chemistry^1,2^. This importance has provided an incentive to create porphyrin analogs, i.e., porphyrinoids, including expanded porphyrins, contracted porphyrins, core-modified porphyrins and isomeric porphyrins^3,4^. The first member of this ever-increasing family, the pentapyrrolic macrocycle sapphyrin^5^, was disclosed by Woodward in 1966 as being an unexpected reaction by-product. Since then, numerous expanded porphyrins have been prepared and studied in light of their flexible conformations, rich coordination chemistry, unique redox activity, and unusual chemical reactivity^6–8^. Often these features have no parallel in the chemistry of simple porphyrins. Early on, for instance, it was found that under certain conditions sapphyrin would undergo nucleophilic attack by methanol to produce a formally non-aromatic derivative^9^. Similar chemistry has been occasionally observed in the case of other porphyrinoid systems^10–14^. However, systematic efforts to exploit this reaction in the context of chemical sensing (“chemosensing”) are to our knowledge unknown. Here we report a “Twisted–Planar–Twisted” dimeric expanded porphyrin system (**D**; Fig. 1) that reacts with methanol selectively and functions as a rudimentary, albeit selective, methanol chemosensor (formally a reaction-based indicator).

**Fig. 2:**
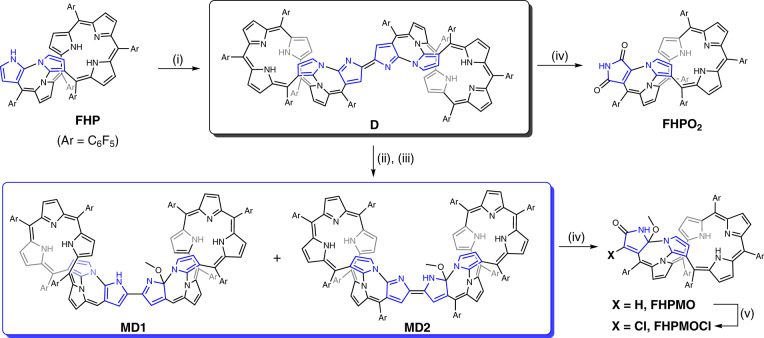
Syntheses of dimers and monomers considered in this study. Conditions: (i) *p*-chloranil, CHCl_3_, reflux; (ii) Cs_2_CO_3_, THF/MeOH; (iii) TFA, THF/MeOH; (iv) CF_3_COOAg, CH_2_Cl_2_/MeOH; (v) FeCl_3_, CHCl_3_/MeOH, reflux. The blue-colored pyrrolic units represent confused pyrroles that are linked with adjacent pyrroles or meso-carbons through C_α_-C_β_ or N-C_β_ atoms.

In recent years, considerable effort has been devoted to extending the chemistry of porphyrin analogs beyond the limits of simple macrocyclic systems to encompass, inter alia, two-/three-dimensional structures^15,16^, linear porphyrin tapes^17,18^, and annular nano-rings^19,20^. Directly linked porphyrin dimers have received particular attention within the context of this general paradigm because of their interesting optical and electrochemical features^21,22^. Confused porphyrins^22,23^ (containing a pyrrole unit linked at the α,*β*′ positions) have also been used for constructing such dimers. In this case, the unoccupied α position may also be exploited to link the subunits in addition to the *β*- and meso-positions that normally serve as inter-porphyrinoid linkage sites (Fig. 1b)^10,24^, reflecting the inherent biased (and relatively high) reactivity of the α-pyrrolic positions. However, the directly linked bipyrroles that result from dimerization are usually severely twisted (reflected in large dihedral angles), presumably as the result of steric hindrance between the planar monomeric subunits. This results in “Planar–Twisted–Planar” dimers (Fig. 1b) and little electronic communication between the individual macrocyclic components. To address this limitation, post-linkage reactions have been used to fuse the two porphyrin units to give “Planar-Planar-Planar” porphyrin dimers^25,26^ (Fig. 1c). Less well explored, and all but unknown, are porphyrinoid dimers with an overall “Twisted–Planar–Twisted” structure (Fig. 1d). Although a number of expanded porphyrins with twisted conformations (e.g., “figure-of-eight” structures) are known, they usually do not exhibit biased reactivity and are not readily dimerized in high yield^27,28^. However, we felt that if the confusion- and expansion-based approaches to porphyrin analog generation were combined, it might prove possible to obtain such dimers. Recently, we reported a neo-fused hexaphyrin **FHP**, a 5,5,5,7-tetracyclic ring system with a figure-of-eight structure, and an apparently accessible α position^29^. Here we show that, when **FHP** is treated with *p*-chloranil in chloroform, an α–α double-bond linked neo-hexaphyrin dimer **D** is obtained in high yield (65%) (Fig. 2). The directly linked central bipyrrolic subunit in **D** is exactly coplanar, and the whole molecule is centrosymmetrical. Also noteworthy is that, relative to **FHP**, dimer **D** displays a narrowed highest occupied molecular orbital–lowest unoccupied molecular orbital (HOMO–LUMO) gap, which leads to greater reactivity. Notably, **D** can react with MeOH under mild conditions while **FHP** is inert toward MeOH under the identical conditions. Upon treating **D** with a mixture of tetrahydrofuran (THF)/methanol under either basic or acidic conditions, a pair of isomeric dimers **MD1** and **MD2** are obtained into which a methoxy moiety is regioselectively attached (Fig. 2). Both **MD1** and **MD2** are characterized by relatively small HOMO–LUMO energy gaps, red-shifted absorption spectral features, and higher reactivity, compared to the **FHP** monomer. As a result, all the three dimers can be readily decomposed to give monomeric daughter products under mild conditions. Importantly, the initial insertion reaction that serves to convert **D** to **MD1** and **MD2** is relatively rapid and apparently selective for MeOH over other common alcohols, including ethanol. It also gives rise to a dramatic color change, from green to brown, that allows methanol to be differentiated from other alcohols visually without need for elaborate instrumentation.

Methanol is a key industrial chemical and a recognized human toxin. It is used as a denaturant in laboratory and industrial-grade ethanol but can also be present in distilled beverages whose quality is not carefully controlled^30,31^. Not surprisingly, therefore, considerable effort has been devoted to the problem of sensing methanol in the presence of ethanol and other potentially competing analytes^32,33^. Currently, physical techniques, such as gas chromatography^34^, Fourier transform infrared spectroscopy^35^, and Raman spectroscopy^36^, define the gold standard for methanol sensing. However, these methods rely on relatively expensive instrumentation. Electrochemical and bioanalytical approaches based on nanomaterials are also known^37,38^. In addition, several chemical indicators have been developed in recent years^39,40^. However, the number of bona fide colorimetric sensors and reaction-based indicators for methanol that allow its presence to be detected through simple color changes remain limited and selectivity remains an issue^41–43^. It is thus possible that the “Twisted–Planar–Twisted” dimer **D** may have a role to play in addressing this need.

Reaction-based chemosensors are usually based on analyte-specific reactions involving appropriately chosen functional groups^44,45^. The post-synthetic functionalization at a specific position of a macrocycles, such as a porphyrinoid, could provide an alternative approach. However, such transformations are usually challenging to implement due to the low selectivity or/and poor yields^46^. As a matter of fact, synthetic macrocycles used as analyte-specific sensors are usually based on supramolecular interactions like weak coordination bonds, hydrogen bonding, and electrostatics^44^, rather than reactivity. In contrast, the rational design and construction of macrocycles incorporating highly reactive N-confused pyrrole units with biased reactivity that can serve as reaction-based chemosensors represents an all but unexplored area of research. As detailed below, the “Twisted–Planar–Twisted” system **D** may serve as a model for this type of putative reaction-based indicator.

## Results

### Synthesis of dimers

When **FHP** was treated with 5 equivalents of tetrachloro-*p*-benzoquinone (*p*-chloranil) under reflux in chloroform for 24 h (Fig. 2), dimer **D** was obtained as a light-green powder in 65% yield. The high-resolution mass spectrum (HRMS) of **D** revealed a molecular ion peak at 2561.1683 (Supplementary Fig. 13, see Supporting Information), assignable to a homodimer of **FHP** produced formally through the elimination of four hydrogen atoms. The ^1^H spectrum of **D** in CDCl_3_ is characterized by features consistent with a symmetrical structure (Supplementary Fig. 1). Moreover, a singlet at 6.95 ppm corresponding to the β protons on the two bridging pyrrole subunits is seen, as would be expected for a dimer linked via the neighboring α-pyrrolic positions. No signal corresponding to the latter protons is seen in **D**, in contrast to what is observed for **FHP**. Four NH proton resonances are seen at *δ* = 11.29 and 9.09 ppm, respectively, as expected given the proposed structure.

Upon treating **FHP** with *p*-chloranil in a mixture of chloroform and methanol (4/1, v/v) under reflux and an N_2_ atmosphere, a heterodimer **MD1** and its isomer **MD2** were separated as brown solids in 18 and 2% yield, respectively. The HRMS of **MD1** exhibits a molecular ion peak at *m*/*z* = 2592.1881 (Supplementary Fig. 14). This value is ca. 31 amu higher than the corresponding peak in the HRMS of **D**, leading us to infer that a methoxy moiety is incorporated into the dimer during the oxidative coupling process. The addition of a single methoxy group was expected to disrupt the symmetry of the overall dimeric molecule. Indeed, ^1^H and ^13^C nuclear magnetic resonance (NMR) spectroscopic analyses of **MD1** in CDCl_3_ revealed features consistent with this presumed lack of symmetry. For instance, separate signals for the two distinct monomeric units could be observed (Supplementary Figs. 3–5). In addition, the peaks at 3.41 and 51.5 ppm, readily assigned to the methoxy substituents, were seen in the ^1^H and ^13^C NMR spectra, respectively (Supplementary Figs. 3, 5). Similar HRMS and NMR spectral features were seen for **MD2** (Supplementary Figs. 6 and 15). All three dimers proved soluble in a variety of organic solvents, including CH_2_Cl_2_, MeCN, THF, toluene, acetone, and dimethylformamide (DMF). This high solubility, which is not recapitulated in most Planar–Planar–Planar dimers, may reflect the twisted nature of the **FHP** unit and an inability to stack effectively in a layer-by-layer fashion.

Dimers **MD1** and **MD2** can also be obtained directly from dimer **D**. When a common inorganic base, such as K_2_CO_3_, Cs_2_CO_3_, or KOH, was added into a solution of **D** in THF and methanol, a rapid color change from green to brown was observed. After stirring for 5 min, **MD1** and **MD2** could be isolated in yields of ca. 56 and 41%, respectively. On the other hand, when common acids, such as HCl, H_2_SO_4_, TFA, and *p*-TsOH, were used instead of the base, **MD1** and **MD2** were obtained in yields of ca. 63 and 15%, respectively. While not studied in detail, the relatively higher yields of **MD1** and the increased **MD1**/**MD2** ratios under acidic conditions can be rationalized in terms of slightly different mechanisms that are considered operative under these disparate conditions (Supplementary Figs. 20 and 21).

In contrast to dimer **D**, the corresponding monomer **FHP** was not observed to react with MeOH under similar conditions, even if the reaction time was prolonged from 5 min to a few days. The higher reactivity of **D** is considered to reflect the dramatically narrowed HOMO–LUMO gap and the strong electron-deficient character of the central bipyrrolic unit (vide infra), both of which are expected to facilitate nucleophilic attack by methanol.

### Decomposition of the dimers to monomers

Although **FHP** is quite stable in MeOH and other common organic solvents in the presence of air, dimers **D**, **MD1**, and **MD2** are much more reactive and tend to decompose to afford monomers. However, the symmetrical dimer **D** and the unsymmetrical dimers **MD1/MD2** exhibit quite different decomposition reactivities. In particular, Ag(I) salts (CF_3_COOAg, AgPF_6_, AgNO_3_, etc.), anhydrous FeCl_3_, DDQ, and even air (O_2_) can induce the decomposition of **D** to give monomer **FHPO**_**2**_ (Fig. 2), along with other unidentified side products. The reaction is not clean and the best yield of **FHPO**_**2**_, obtained when CF_3_COOAg was used as the oxidant, was no higher than 8%. The ^1^H NMR spectrum of **FHPO**_**2**_ revealed the absence of a methoxy moiety within the expected 3.0–4.0 ppm chemical shift range (Supplementary Fig. 7). HRMS analysis (*m*/*z* = 1313.0767, [M + H]^+^; Supplementary Fig. 16) provided support for the conclusion that two oxygen atoms are incorporated into **D** to give **FHPO**_**2**_ during the oxidative cleavage process. In contrast to what is seen for **D**, under similar reaction conditions both **MD1** and **MD2** undergo decomposition to afford a methoxy-bearing neo-fused hexaphyrinone **FHPMO** in relatively clean fashion. For instance, when **MD1** and **MD2** were treated separately with 8 equivalents of CF_3_COOAg in CH_2_Cl_2_/MeOH, **FHPMO** was obtained in high yield (ca. 92% in both cases). Electrospray ionization–HRMS analysis (*m*/*z* = 1329.1089, [M + H]^+^; Supplementary Fig. 17) revealed that **FHPMO** incorporates an oxygen atom in addition to a methoxy moiety. A strong peak ascribed to the methoxy moiety was observed at 3.47 ppm in the ^1^H NMR spectrum (Supplementary Fig. 11). We thus conclude that the silver(I)-promoted decomposition reactions of the unsymmetrical dimers **MD1** and **MD2** are much simpler and cleaner than those of the homodimer **D**, a difference attributed to the effect of the methoxy group. Furthermore, **FHPMO** was found to undergo a highly regioselective monochlorination reaction to afford **FHPMOCl** in 78% yield when treated with anhydrous FeCl_3_ in chloroform/methanol. Evidence for the formation of **FHPMOCl** came from HRMS analyses (*m*/*z* = 1363.0712, [M + H]^+^, Supplementary Fig. 18), ^1^H/^13^C NMR spectroscopy (Supplementary Figs. 11 and 12), and a single crystal X-ray diffraction analysis (vide infra).

### Single crystal structures

**Fig. 3:**
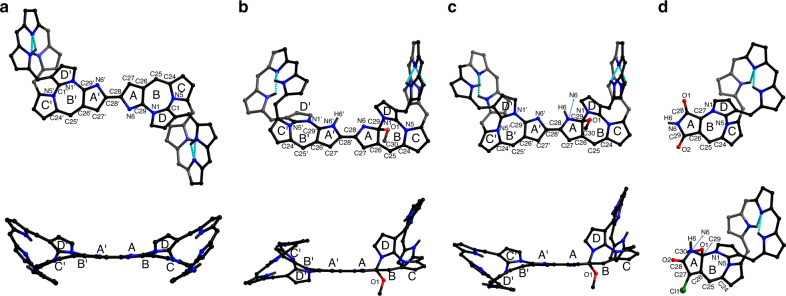
Crystal structures of the dimers and monomers considered in this study. Complementary views of the molecular structures of **D** (**a**), **MD1** (**b**), and **MD2** (**c**) and molecular structures of **FHPO**_**2**_ (**d**, top) and **FHPMOCl** (**d**, bottom). Pentafluorophenyl substituents, solvents, and the hydrogen atoms attached to the carbon atoms are omitted for clarity. The dotted lines represent intramolecular hydrogen bonds inferred on the basis of the metric parameters. N, O, and Cl atoms are denoted with balls of blue, red, and green colors, respectively. C and H atoms (linked to N atoms) are denoted with black balls, with smaller balls used for the latter.

Further insight into the structures of **D**, **MD1**, **MD1**, **FHPO**_**2**_, and **FHPMOCl** came from X-ray diffraction analyses. As shown in Fig. 3, **D** displays a “Z”-shaped *trans* configuration with the “figure-of-eight” conformation of the hexaphyrin subunits reserved in the dimer (Fig. 3a). Dimer **D** is centrosymmetric and the two units are bridged by the C28-C28’ double bond (1.393(8) Å) (Supplementary Fig. 29; for numbering and subunit labels, see Fig. 3). The two monomeric units are linked through the coplanar bipyrrolic bridging unit A-A’, with pyrroles C (C’) and D (D’) slightly tilted off this plane as reflected in the relatively small dihedral angles of 4.5° and 14.4°, respectively. The relative planarity of the multiply fused constituent cycles (C-D-B-A-A’-B’-D’-C’) was expected to favor effective π-conjugation and associated electronic coupling between the two hexaphyrin subunits. To the extent this proved true, it would be expected to have a significant effect on the electronic and electrochemical features, as in fact is seen by experiment (vide infra).

In contrast to **D**, the unsymmetrical heterodimers **MD1** and **MD2** adopt *cis* configurations about the bridging C28-C28’, for which bond lengths of 1.416(5) and 1.379(6) Å are observed, respectively. The difference in the latter values is ascribed to the presence of what may be considered as being formal single and double bonds, respectively (Fig. 3b, c and Supplementary Figs. 30 and 31). The bipyrrolic units A-A’ in **MD1** and **MD2** both show good planarity and are characterized by small dihedral angles of 4.6° and 5.9°, respectively.

In both unsymmetrical dimers, a methoxy group is attached to one of the hexaphyrin units. In **MD1**, this group is attached at C29, which is best described as being an sp^3^-hybridized carbon center. The surrounding C-N, C-O and C-C bond lengths are in the range of 1.40–1.54 Å, typical for single bonds. It is noteworthy that pyrrole D is tilted from the A-A’ plane with a relatively large dihedral angle of 80.6°. On the other hand, the methoxy-free subunit of **MD1** is more planar, with pyrroles C’ and D’ tilted from A-A’ at smaller dihedral angles of 37.9° and 36.8°, respectively. In **MD2**, the corresponding values are 31.9° and 34.8°, respectively, slightly smaller than those observed for **MD1**. These dihedral angles are all larger than the corresponding values in **D**, indicative of greater distortion.

### Ultraviolet/visible/near-infrared (UV/vis/NIR) absorption spectra

**Fig. 4:**
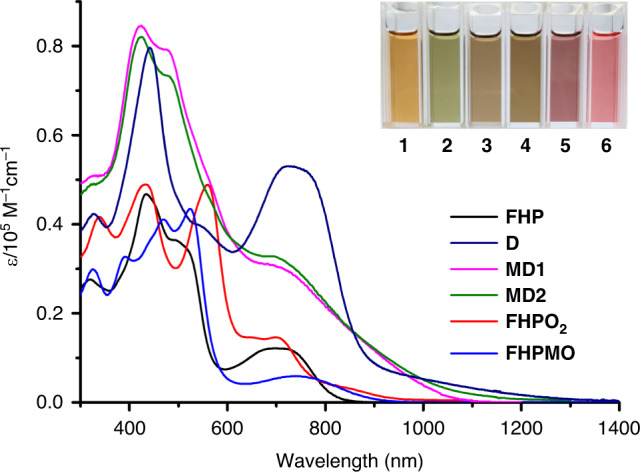
UV/vis/NIR absorption spectra of the dimers and monomers considered in this study as recorded in CH_2_Cl_2_. The inset shows photographs of the corresponding solutions in CH_2_Cl_2_ (1.0 × 10^−5^ M for all compounds). Here the numbers **1**–**6** refer to **FHP**, **D**, **MD1**, **MD2**, **FHPO**_**2**_, and **FHPMO**, respectively.

Compared with **FHP**, the absorption bands of the dimers tail further into the red (Fig. 4). They are also roughly twice as intense, as might be expected for dimers incorporating twice the number of chromophores on a per mole basis. In the case of **D**, a Soret-like band with a sharp peak centered at ca. 442 nm is seen, along with a broad and very intense Q-like band centered at 727 nm that tails out to ca. 1300 nm. In contrast, the spectrum of the unsymmetrical dimer **MD1** is characterized by a split Soret-like band with local maxima centered at 423 and 479 nm, as well as a shoulder at ca. 693 nm. The absorption edge of **MD1** extends to ca. 1070 nm, a reduction in the tailing compared to **D** that may reflect interruption in the local conjugation as a consequence of the incorporated methoxy group. The absorption spectrum of heterodimer **MD2** resembles that of **MD1**. However, the absorption edge of **MD2** approaches ca. 1250 nm, an observation consistent with the more extended conjugation expected for **MD2** as the result of the double bond link that connects the two monomeric units (Supplementary Fig. 34). The absorption bands of **FHPO**_**2**_ and **FHPMO** roughly mirror those of **FHP**, consistent with their monomeric structures. However, their absorption features tail a bit further to the red (to ca. 960 and 910 nm for **FHPO**_**2**_ and **FHPMO**, respectively). In addition, the Soret-like feature is strongly split in the case of **FHPO**_**2**_.

### Electrochemistry

**Fig. 5:**
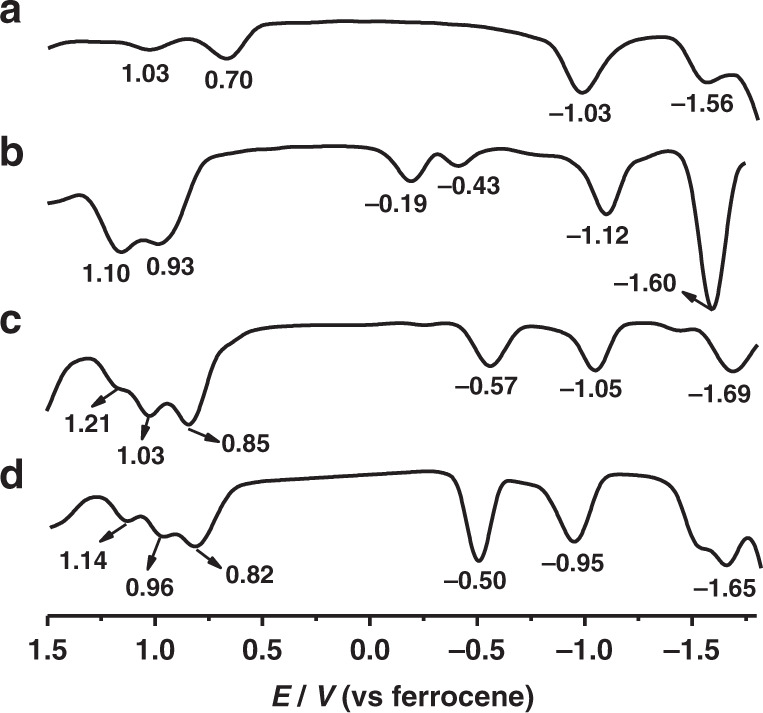
Electrochemical studies of the dimers considered in this study and the monomer FHP. Differential pulse voltammograms (DPV) of **FHP** (**a**), **D** (**b**), **MD1** (**c**), and **MD2** (**d**). DPV measurements were conducted in anhydrous CH_2_Cl_2_ containing 0.1 M tetra-*n*-butylammonium perchlorate (TBAClO_4_) as the supporting electrolyte. A three-electrode cell was used: a glassy carbon (working electrode), platinum wire (counter electrode), and Ag/AgCl (reference electrode). Ferrocene/ferrocenium (Fc/Fc−) was used as an external reference.

The electrochemical properties of the dimers and monomers were studied using cyclic voltammetry (CV) and differential pulse voltammetry (DPV) in CH_2_Cl_2_ containing 0.1 M tetra-*n*-butylammonium perchlorate (TBAClO_4_) as the supporting electrolyte (Fig. 5 and Supplementary Figs. 27 and 28). All the three dimers, **D**, **MD1**, and **MD2**, displayed electrochemical features that were noticeably different from those of **FHP**. Homodimer **D** displays two partially overlapped quasi-reversible oxidation waves at 0.93 and 1.10 V, which are anodically shifted compared with **FHP** (0.70, 1.03 V). Four different reduction processes were observed and the potentials for the first two are anodically shifted to −0.19 and −0.43 V, respectively, compared to the corresponding value of −1.03 V observed for **FHP**. The positively shifted reductions are considered to reflect the more electron-deficient nature of **D**. The first and second reduction potentials can be viewed as a split reduction couple, and the large potential difference between them (Δ*E*_red_ = 0.24 V) is taken as evidence of effective electronic communication^47,48^ between the two hexaphyrin subunits mediated by the planar bipyrrolic linking unit. As true in the case of the UV-vis-NIR spectral studies (vide supra), the unsymmetrical dimers **MD1** and **MD2** demonstrate similar electrochemical features. Three slightly anodically shifted oxidation waves (versus two processes for **FHP** and **D**) were observed for these unsymmetrical dimers, with the values for first oxidation potentials being 0.85 and 0.82 V, respectively. Similar to what proved true for **D**, the first reduction waves of **MD1** and **MD2** (−0.57, −0.50 V) are anodically shifted compared to **FHP** (−1.03 V), although to a lesser extent than **D** (−0.19 V). Based on the DPV data, the electrochemical HOMO–LUMO gaps between the first oxidation and first reduction potentials of **D**, **MD1**, and **MD2** were estimated to be 1.12, 1.42, and 1.32 V, respectively (Supplementary Table 1). This observation is consistent with what was seen for the tailing absorption band edges. The inferred HOMO–LUMO gaps for the dimers are much smaller than that for **FHP** (1.73 eV). Again, this is consistent with effective π-conjugation occurring to a greater or lesser extent throughout the three dimers.

### Theoretical calculations

**Fig. 6:**
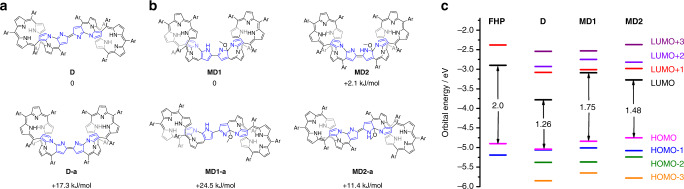
DFT-calculated relative/orbital energies at the B3LYP/6-31G(d) level of theory. **a** Computationally derived relative energies of **D**-**a**; and the putative *cis* isomer of **D**; **b** computational relative energies of the unsymmetrical dimers **MD1**, **MD2**, and the possible *trans* isomers **MD1-a** and **MD2-a**; **c** selected molecular orbital energies for **FHP** and dimers **D**, **MD1**, and **MD2**. Ar = C_6_F_5_. The blue-colored pyrrolic units in **a** and **b** represent confused pyrroles that are linked with adjacent pyrroles or meso-carbons through C_α_-C_β_ or N-C_β_ atoms.

Density functional theory (DFT) calculations using the Gaussian 16 program package^49^ were performed on the dimers in an effort to understand further their distinct structures and properties. First, the energies of both the putative *cis* and *trans* isomers were calculated at different energy levels. The B3LYP/6-31G(d)^50^ and extended basis sets (6-31G(d,p) and 6-311G(d,p))^51,52^ all gave similar values for the energy barriers between isomers **D**/**D-a** and **MD1**/**MD1-a**/**MD2**/**MD2-a**, respectively. The use of the BMK functional^53^ gave similar results as the B3LYP functional. In addition, the Grimme D3 correction^54^ was included for the B3LYP functional; this gave the same trend (Supplementary Tables 5 and 6). It was found that the relative energy of the *trans* dimer (**D**) is 17.3 kJ/mol lower than the corresponding *cis* isomer **D-a** (Fig. 6a). Such a computational finding is consistent with the observation that **D** is obtained as the sole product and in relatively high yield when **FHP** is subject to oxidative coupling. In contrast, the isomeric *cis* dimers (**MD1** and **MD2**) were both calculated to lie at much lower energies than their corresponding *trans* dimers **MD1-a** and **MD2-a**; again, this finding agrees well with experiment, specifically the finding that neither **MD1-a** nor **MD2-a** could be detected under the conditions of the methanol insertion reaction (Fig. 6b). On the other hand, the energy of **MD2** is slightly higher than its isomer **MD1** (by +2.1 kJ/mol), roughly consistent with the observation that the synthetic yields for **MD1** are higher than **MD2** under the various synthetic conditions tested in the context of this study (vide supra).

**Fig. 7:**
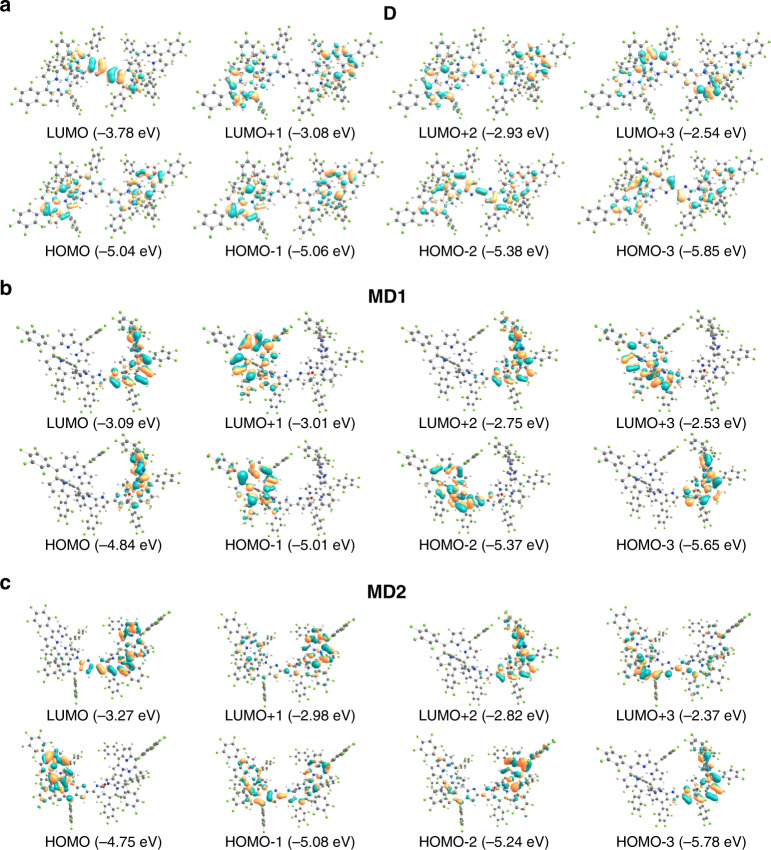
Selected contour plots of the DFT-optimized structures and various orbitals for the compounds of this study. **a**–**c** stands for **D**, **MD1**, and **MD2**, respectively. The corresponding energy values are included in parentheses. The isosurface control value is 0.03 a.u. The occupied molecular orbitals are highlighted with blue and orange colors. C, N, O, H, and F atoms are denoted with balls of gray, red, white, and green colors, respectively.

Notably, the frontier molecular orbitals of **D**, **MD1**, and **MD2** are all remarkably different (Fig. 7a–c). For **D**, the LUMO orbital is mainly distributed over the central multiply fused unit showing a relatively low energy of −3.78 eV. This results in a narrow HOMO–LUMO gap of 1.26 eV, which is ca. 0.74 eV smaller than that of **FHP**. This is taken as evidence of the pronounced electron-deficient character of the two bridging imino-type pyrrolic units (A-A’), an inference that, in turn, explains the susceptibility to nucleophilic attack by MeOH (or its alkoxide anion) to form **MD1/2**. Such an inference is consistent with the positive shifts in the first two reduction waves seen in **D** (vide supra). The HOMO and HOMO-1 of **D** are almost degenerate (orbital energies: −5.04 and −5.06 eV, respectively) with the orbitals extended over the two hexaphyrin macrocycles except for the central multiply fused units. The high symmetry and relatively strong interaction between the two hexaphyrin units may be responsible for the near-degeneracy^54–57^. For **MD1**, the HOMO and LUMO are both delocalized over the methoxy-containing hexaphyrin unit. Specifically, the LUMO is delocalized over the whole hexaphyrin macrocycle, while the HOMO is distributed over the macrocycle except for the fused tetracyclic unit (ABCD rings; see Fig. 3b). No evidence of degenerate orbitals is seen for the HOMO of **MD1**, which may reflect the reduced symmetry of this unsymmetrical dimer relative to **D**. In the case of **MD2**, the LUMO orbital is mainly located on the methoxy-containing hexaphyrin moiety, as well as the pyrrolic unit (A’) of the other hexaphyrin moiety. The HOMO, however, is mainly distributed over the methoxy-free hexaphyrin unit. The obviously different orbital distributions of **MD1** and **MD2** can be rationalized by the fact that the conjugation pathway in **MD1** is disturbed, which results in relatively weak interaction between the two hexaphyrin units, while a fuller conjugated pathway exists in **MD2** (Supplementary Fig. 34).

The energy levels of the LUMO orbitals for **MD1** and **MD2** are dramatically negatively shifted to −3.09 and −3.27 eV, respectively, relative to **FHP** (−2.90 eV). The corresponding HOMO levels are located at −4.84 and −4.75 eV, respectively. This represents a slight positive shift compared to **FHP** (−4.90 eV). As a result of the dramatically lowered LUMO orbitals, the HOMO–LUMO gaps of **D**, **MD1**, and **MD2** are 1.26, 1.75, and 1.48 eV, respectively (Fig. 6c). They are thus much smaller than those of **FHP** (2.00 eV). These reductions in the HOMO–LUMO gaps are fully consistent with the trends inferred from the spectroscopic and electrochemical analyses discussed above.

As mentioned above, the experimental absorption spectra of the three dimers appear to be very simple in the low energy region beyond 600 nm. These features are quite different from those of traditional porphyrin dimers^55–57^. To provide further insight into these observations and understand the dramatically different absorption features for the three dimers, time-dependent (TD) DFT calculations were carried out. At first, the energy of the S_1_ state (nm) for the dimers were calculated by different functionals (B3LYP, PBE0, ω-B97XD, M062X, CAM-B3LYP, BMK, etc.) using the 6-31G(d) basis set and the polarizable continuum model (PCM) model (dichloromethane (DCM) as a solvent) (Supplementary Table 7). Relative to the observed absorption spectra, both the B3LYP^58^ and PBE0^59^ functionals underestimate the energy of the first S_0_ → S_1_ transition for **D** and **MD2**. On the other hand, the long-range corrected CAM-B3LYP^60^ functional slightly overestimates the energy of the S_0_ → S_1_ electronic transition for **MD1**, while it overestimates the energy of the S_0_ → S_1_ electronic transition for dimer **D**. For the ω-B97XD^61^ and M062X^62^ functionals, the difference between the S_0_ → S_1_ electronic transition of **MD1** and **MD2** is not fully revealed. In contrast, the BMK functional, which usually gives improved accuracy for transition state barriers^53^, gave relatively reasonable results for all the three dimers compared to the other functionals employed. For **D**, the exceptionally strong and broad absorption band in the 600–900 nm region can be rationalized in terms of a HOMO-2 → LUMO transition with quite a large oscillator strength (*f* = 1.81). Both the HOMO-2 and LUMO orbitals are localized on the bridging bipyrrolylidene fragment (Fig. 7a), indicating that this band mainly originates from a localized π–π* transition, instead of an intramolecular charge transfer (ICT) from the hexaphyrin core to the central multiply fused linking unit^63–65^. The Q-like band of compound **D** corresponds to the quasidegenerated S_0_ → S_1_ and S_0_ → S_2_ transitions, and the oscillator strengths (*f* = 0.26/0.005) are much smaller than those of the HOMO-2 → LUMO transition (*f* = 1.81) (Supplementary Fig. 35 and Supplementary Table 9). Hence, the lower energy band is observed to be submerged within the strong broad band in the 600–900 nm region. As a result, no clearly resolved Q-like bands could be observed in contrast to other reported dimers characterized by different linking modes^55^.

As can be seen from Fig. 7b, the occupied and unoccupied molecular orbitals of **MD1** are mainly localized on either one of the hexaphyrin units. Therefore, the calculated lowest energy transitions of **MD1** at 817 and 698 nm, mainly corresponding to HOMO-1 → LUMO and HOMO → LUMO+1 excitations, respectively, are both characteristic of weak oscillator strengths (*f* ≈ 0.1) because of the ICT nature associated with the electronic distribution (Supplementary Fig. 36 and Supplementary Table 10). On the other hand, the high energy transitions in the range of 400—600 nm (Soret-like band) demonstrate relatively larger oscillator strengths (*f* = 0.3–0.7). Therefore, absorptions from the low energy transitions are submerged under the higher energy ones. As a result, **MD1** exhibits only a shoulder peak at ca. 693 nm instead of well-resolved Q-like bands as observed for the traditional porphyrin dimers^55–57^. Similar to **MD1**, the lowest energy excitations of **MD2** also correspond to HOMO-1 → LUMO and HOMO → LUMO+1 transitions (Supplementary Fig. 37 and Supplementary Table 11). Notably, the HOMO-1 orbitals are located over both of the hexaphyrin fragments, and the HOMO-1 → LUMO transition is symmetry allowed, leading to a larger oscillator strength (0.37) than that of **MD1**, a finding that serves to rationalize the slightly stronger Q-like band and longer wavelength band edge for **MD2** in comparison with **MD1**.

### Methanol response

**Fig. 8:**
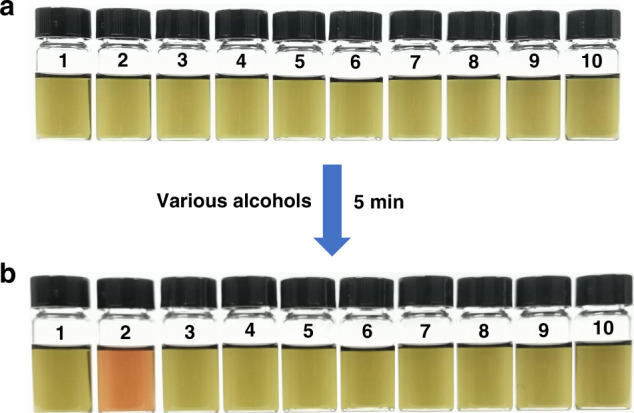
Photographs of the D solutions in response to various alcohols. **a** Vials containing **D** (1.3 × 10^−5^ mol/L) and NaOH_(s)_ (10 mg) in THF (3.0 mL). **b** The same vials as in **a** after adding 3.0 μL of the following alcohols (from left to right and numbered **1**–**10**): none, methanol, ethanol, 1-propanol, 2-propanol, 1-butanol, 2-butanol, phenylcarbinol, ethylene glycol, and glycerol, respectively.

As mentioned above, a dramatic solution color change from light green to brown was observed accompanying with the conversion from **D** to **MD1**/**2**. As a matter of fact, solutions of **D** in THF under basic conditions are very sensitive to methanol, while the response to ethanol and other alcohols is considerably slower. Presumably, this reflects the somewhat lowered nucleophilicity and greater steric hindrance of these higher alcohols. In principle, this reactivity difference could be used to differentiate methanol from other alcohols. As a test of this hypothesis, various alcohols (3.0 μL corresponding to ca. 25 mM after dilution) were added, respectively, into THF solutions of **D** (1.3 × 10^−2^ mM in 3.0 mL) in the presence of excessive NaOH_(s)_ (10 mg). Under these conditions, a discernible color change was found for the methanol sample within 5 min (Fig. 8). However, no obvious color change was observed in the case of any of the other alcohols during the same 5-min time period. Kinetic studies, involving the response of **D** toward methanol and ethanol as a function of time, were carried and provide support for the fact that these two alcohols give rise to remarkably different reaction rates (Supplementary Figs. 22 and 23). Notably, when the amount of added methanol was decreased to 0.50 μL (corresponding to ca. 4.2 mM after dilution), a color change from green to light brown still occurs, although 24 h were required to effect essentially complete conversion (Supplementary Figs. 24 and 25). On this basis, we conclude that methanol may be readily differentiated from other alcohols by simply monitoring whether the distinctive color change is produced during a set time period. Furthermore, the detection limit^66^ of methanol in THF was estimated to be ca. 0.037 mM based on the 24-h reaction timeframe (Supplementary Fig. 26). On this basis, we proposed that **D** may emerge as a promising methanol indicator that can operate in the absence of relatively expensive analytical instrumentation.

## Discussion

Oxidative coupling of an unusual twisted neo-fused hexaphyrin (**FHP**) with a protruding N-confused pyrrole gives rise to the “Twisted–Planar–Twisted” homodimer **D** with a *trans* configuration in high yield, leading us to suggest that introducing a reactive subunit into a twisted porphyrinoid is a promising approach for constructing functional dimers or even larger oligomers. Upon adding methanol to the reaction mixture under basic or acid conditions, regioselective addition of a methoxy moiety to **D** occurs to afford two isomeric heterodimers, **MD1** and **MD2**, both of which contain *cis*-type central linkages. A strong interaction between the two hexaphyrin subunits of **D** is inferred from its spectral and physical properties and is ascribed to the presence of a coplanar central bipyrrolic linking unit and the roughly coplanar arrangement of the appended neo-fused hexaphyrin subunits. Compared with **D**, reduced intramolecular electronic interactions are seen in the case of **MD1** and **MD2**. These latter isomeric species differ somewhat from one another, reflecting differences in their respective conjugation pathways and molecular conformations. The fact that **D** reacts readily and selectively with methanol under readily defined reaction conditions allows for the qualitative differentiation of methanol from ethanol and other alcohols by simple visual means. The associated sensing function could prove useful in detecting methanol in either an industrial or beverage-related setting. Further studies of this possibility are ongoing in our laboratory.

The studies reported here not only illustrate a promising approach to synthesizing porphyrinoid dimers by oxidatively coupling the corresponding monomers through their accessible α-pyrrolic positions but also provide an approach for developing reaction-based chemosensors. The latter potential application takes advantage of the fact that the present dimers allow for biased high reactivity that reflects a synergetic effect of reduced HOMO–LUMO energy gaps and the presence of reactive N-confused units. While further work will be needed to develop the present findings as a potentially generalizable strategy, the approach to sensing detailed here could provide a useful complement to current methods that typically involve the rational design of specific receptors or reactive constructs.

## Methods

### General methods

Commercially available solvents and reagents were purchased from Adamas-Beta and used without further purification unless otherwise mentioned. Thin-layer chromatography was carried out on aluminum sheets coated with silica gel 60 F254 (MERCK). ^1^H NMR (400 MHz) spectra were obtained using a Bruker AM 400 spectrometer. Chemical shifts are reported relative to tetramethylsilane (*δ* = 0) in ppm. ^13^C NMR spectra were recorded at 101 MHz (Bruker AM 400) and the chemical shifts were reported relative to CDCl_3_ (*δ* = 77.00), (CD_3_)_2_CO (*δ* = 29.84), or DMSO-*d*_*6*_ (*δ* = 39.52) in ppm; see Supplementary Figs. 1–12. HRMS analyses were performed using a Waters LCT Premier XE spectrometer or Bruker Solarix XR FTMS instrument for CH_2_Cl_2_ solutions; see Supplementary Figs. 13–18. UV-vis-NIR absorption spectral studies were carried out using a Shimadzu UV2600 spectrophotometer with all spectra being recorded at room temperature.

### Syntheses of compounds

Homodimer **D** was synthesized through a one-pot oxidation of the monomer **FHP** in methanol-free solvents, in yields of ca. 65%. The unsymmetrical dimers **MD1** and **MD2** can be obtained either from **FHP** or **D**, both in the presence of methanol. The monomers **FHPO**_**2**_ and **FHPMO** were obtained by the direct oxidation of **D** and **MD1** (**MD2**), respectively (see Supplementary Methods). The target compounds were fully characterized by single crystal X-ray diffraction analyses, as well as by NMR spectroscopy and HRMS analyses (Supplementary Methods).

### Crystallography

Single crystals of **D** were obtained by the slow recrystallization from the DCM/heptane solution; crystals of **D1** and **FHPMOCl** were obtained by the slow recrystallization from their DCM/octane solutions, and crystals of **D2** and **FHPMO**_**2**_ were obtained by the slow evaporation of their acetonitrile/water solutions. Single crystal X-ray analyses were performed on a SMART APEX equipped with CCD detector (Bruker) using MoKα (graphite, monochromated, *λ* = 0.71073 Å) radiation or CuKα (graphite, monochromated, *λ* = 1.54178 Å) radiation. The structures were solved by the direct method of SHELXS-97/2014/2018 and refined using the SHELXL-97/2014 or Olex2 1.2 program^49,67,68^. The positional parameters and thermal parameters of non-hydrogen atoms were refined anisotropically on *F*^2^ by the full-matrix least-squares method. Hydrogen atoms were placed at calculated positions and refined riding on their corresponding carbon atoms. Detailed crystallographic data are provided in Supplementary Tables 2 and 3.

### Electrochemical measurements

CV and DPV studies were carried out at 298 K using a CHI-730C Electrochemical Workstation. A three-electrode cell consisting of a glassy carbon (working electrode), platinum wire (counter electrode), and Ag/AgCl (reference electrode) attached to a Chi-730D/620E electrochemistry station were used, with the potentials calibrated to the ferrocenium/ferrocene (Fc^+^/Fc) couple. Absolute DCM (CH_2_Cl_2_) was purchased from Adamas-Beta and used as received and TBAClO_4_ was purchased from Fluka Chemika, recrystallized from ethanol, and dried under vacuum at 40 °C for at least 1 week prior to use.

### Theoretical calculation details

All calculations were carried out using the Gaussian 16 suite of programs^49^. The geometries of all molecules under study were optimized using B3LYP^58^ and the 6-31G(d) basis set^50^ for all the atoms directly. No adjustment to the effective core potential was applied. All the optimized geometries were checked as the real minima on the potential energy surface by means of frequency calculations. At the optimized geometries, TD DFT calculations with the hybrid BMK functional^53^ were carried out to simulate the absorption spectra. Solvent effects arising from CH_2_Cl_2_ were accounted for using the PCM^69^.

## Supplementary information

Supplementary Information

## Data Availability

CCDC 1886415-1886417, 1985682, and 1985685 contain the supplementary crystallographic data for this paper. These data can be obtained free of charge from The Cambridge Crystallographic Data Centre via www.ccdc.cam.ac.uk/data_request/cif. All other data supporting the findings of this study are available from the corresponding authors upon request.
