# Supplementary material for: Twisted-Planar-Twisted expanded porphyrinoid dimer as a rudimentary reaction-based methanol indicator
Source: Nat Commun. 2020 Oct 20;11:5289. doi: 10.1038/s41467-020-19118-9 (PMC7576827; doi:10.1038/s41467-020-19118-9)
Supplement: Supplementary file 1 — Supplementary Information [file 41467_2020_19118_MOESM1_ESM.pdf]

Supplementary Information

Twisted-Planar-Twisted expanded porphyrinoid dimer as a rudimentary reaction-based methanol indicator

Qizhao Li[1], Chengjie Li[1], Glib Baryshnikov[2], Yubin Ding[1], Chengxi Zhao[1], Tingting Gu[3], Feng Sha[1], Xu Liang[3], Weihua Zhu[3], Xinyan Wu[1], Hans Ågren[2], Jonathan L. Sessler[4★] & Yongshu Xie[1★]

Supplementary Figures (* solvent and impurities)

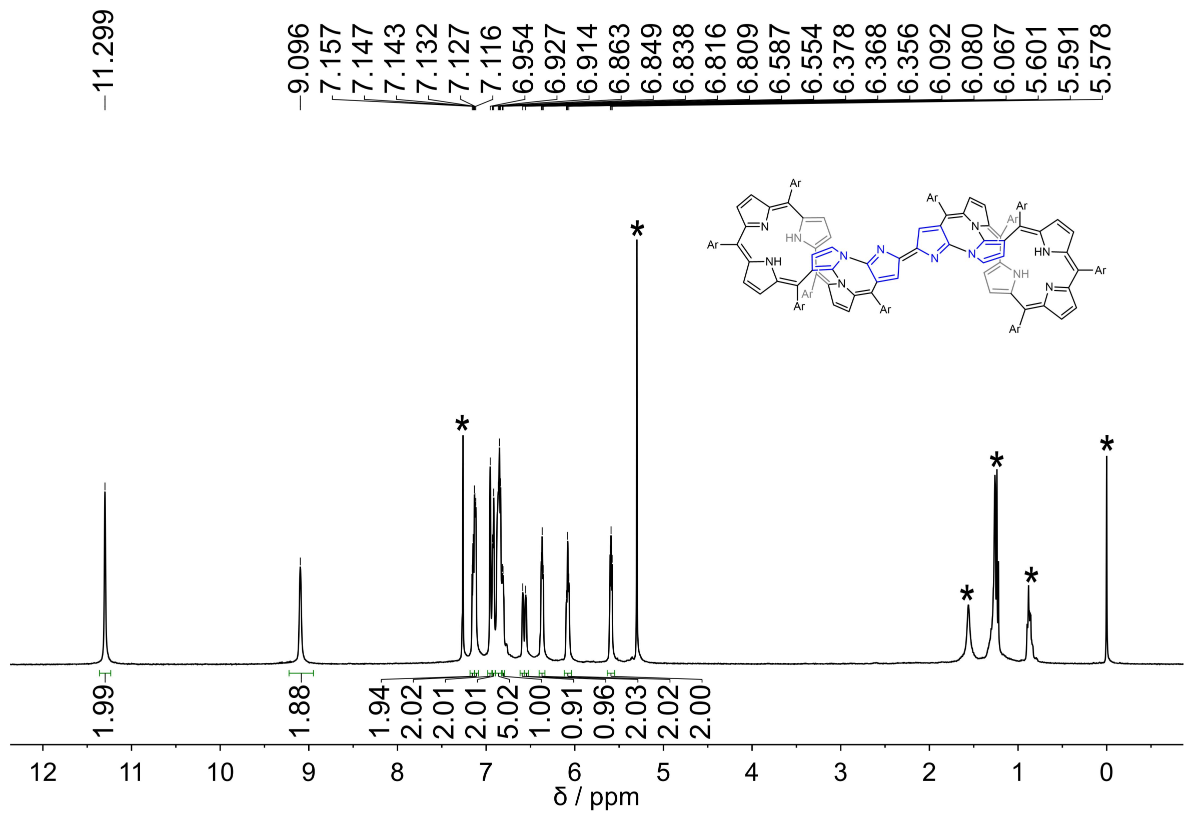

Supplementary Figure 1. ^{1}H NMR spectrum of **D** in $CDCl_3$.

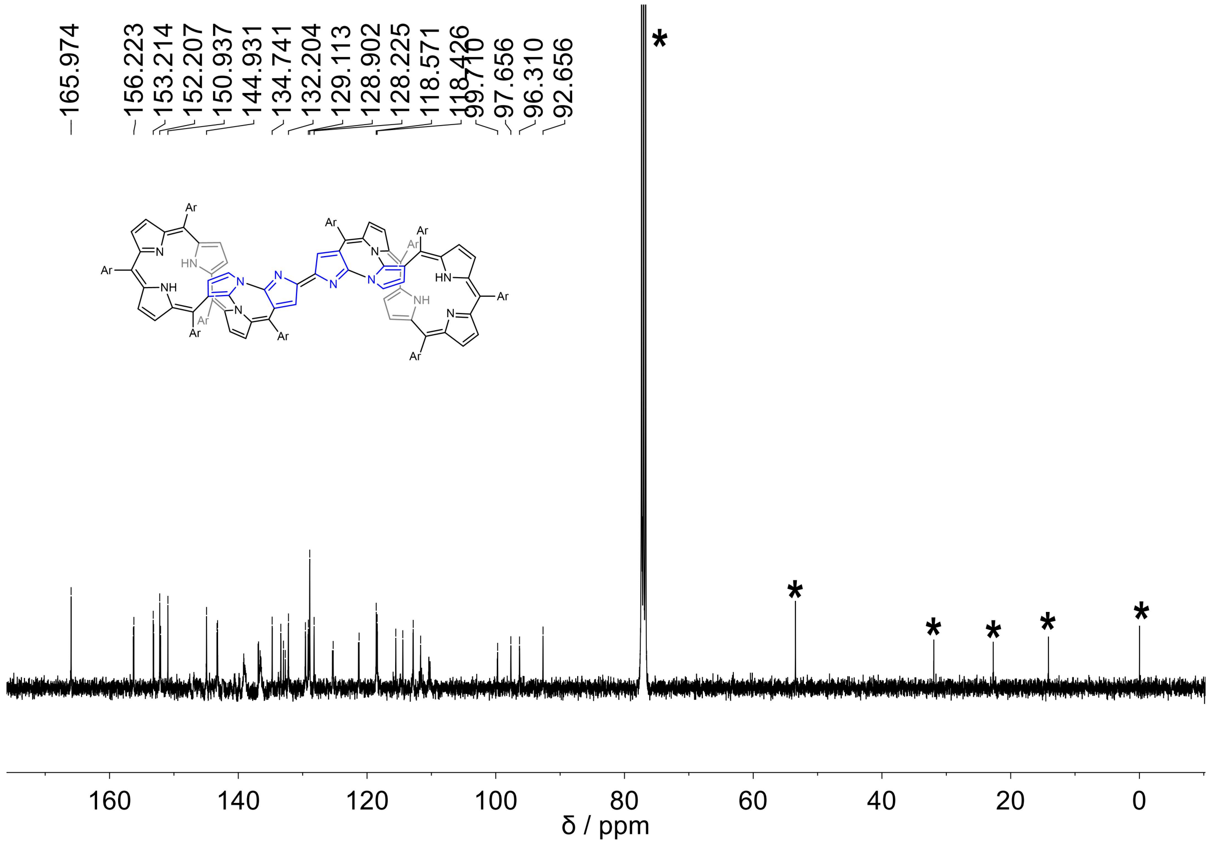

Supplementary Figure 2. ^{13}C NMR spectrum of **D** in $CDCl_3$.

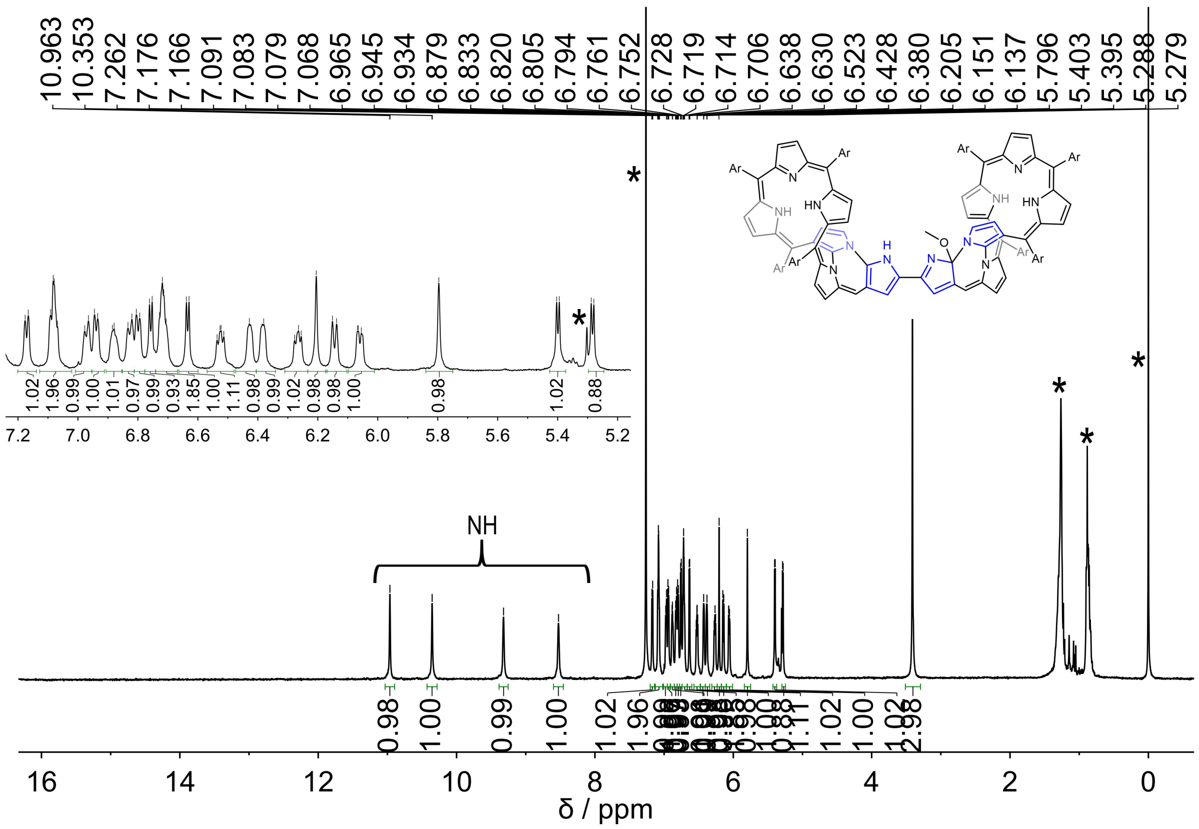

Supplementary Figure 3. ^{1}H NMR spectrum of **MD1** in $CDCl_3$. Note: The outer NH proton signal cannot be observed in $CDCl_3$.

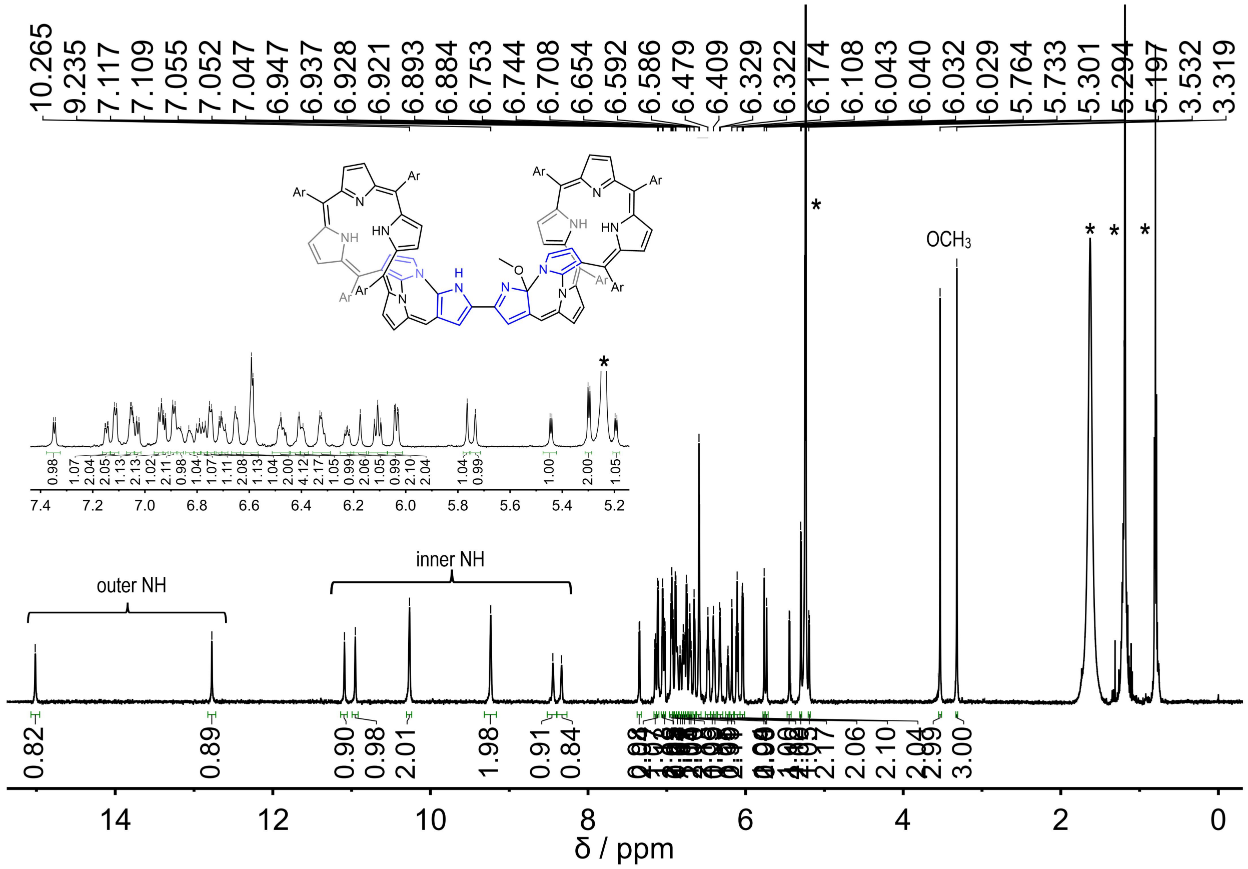

Supplementary Figure 4. ^{1}H NMR spectrum of **MD1** in CD_2Cl_2.(The outer NH can be observed in CD_2Cl_2, while two sets of signals were observed probably due to the presence of two isomers.)

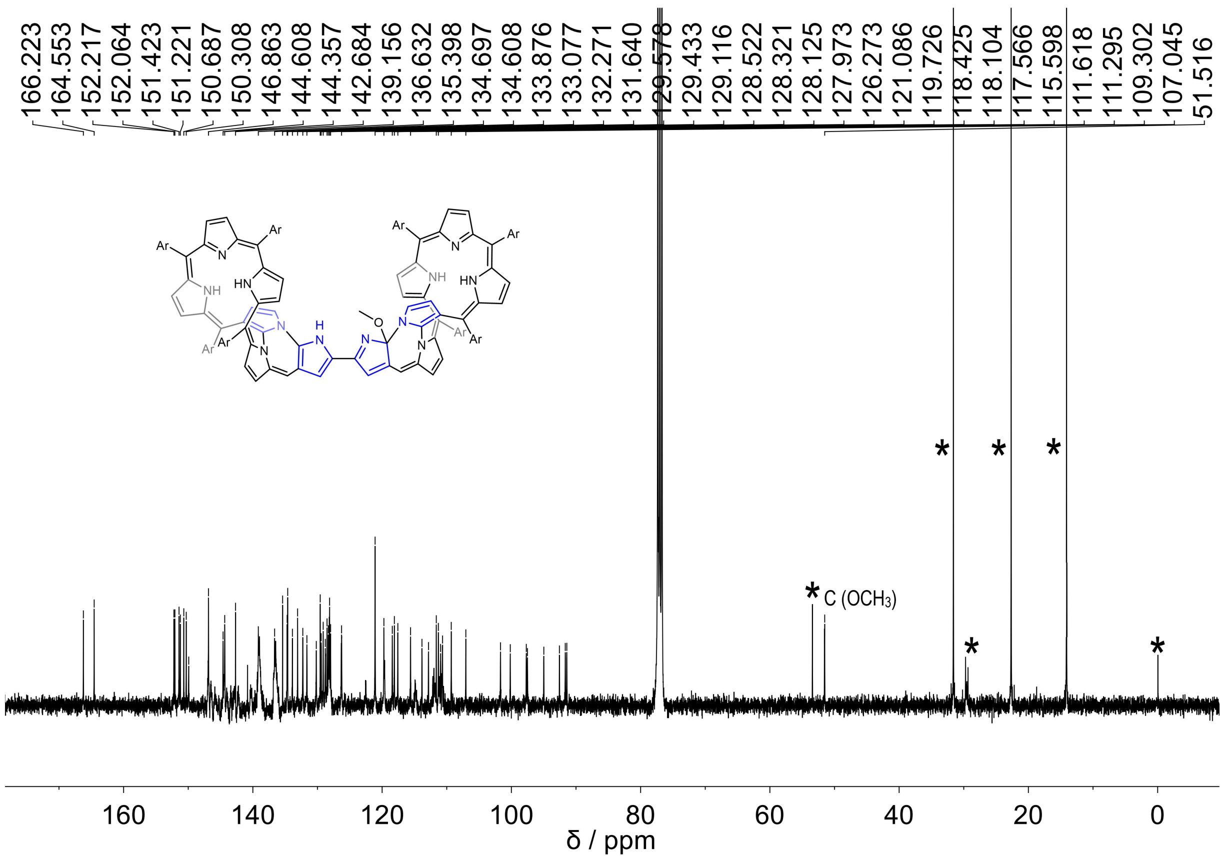

Supplementary Figure 5. ^{13}C NMR spectrum of **MD1** in $CDCl_3$.

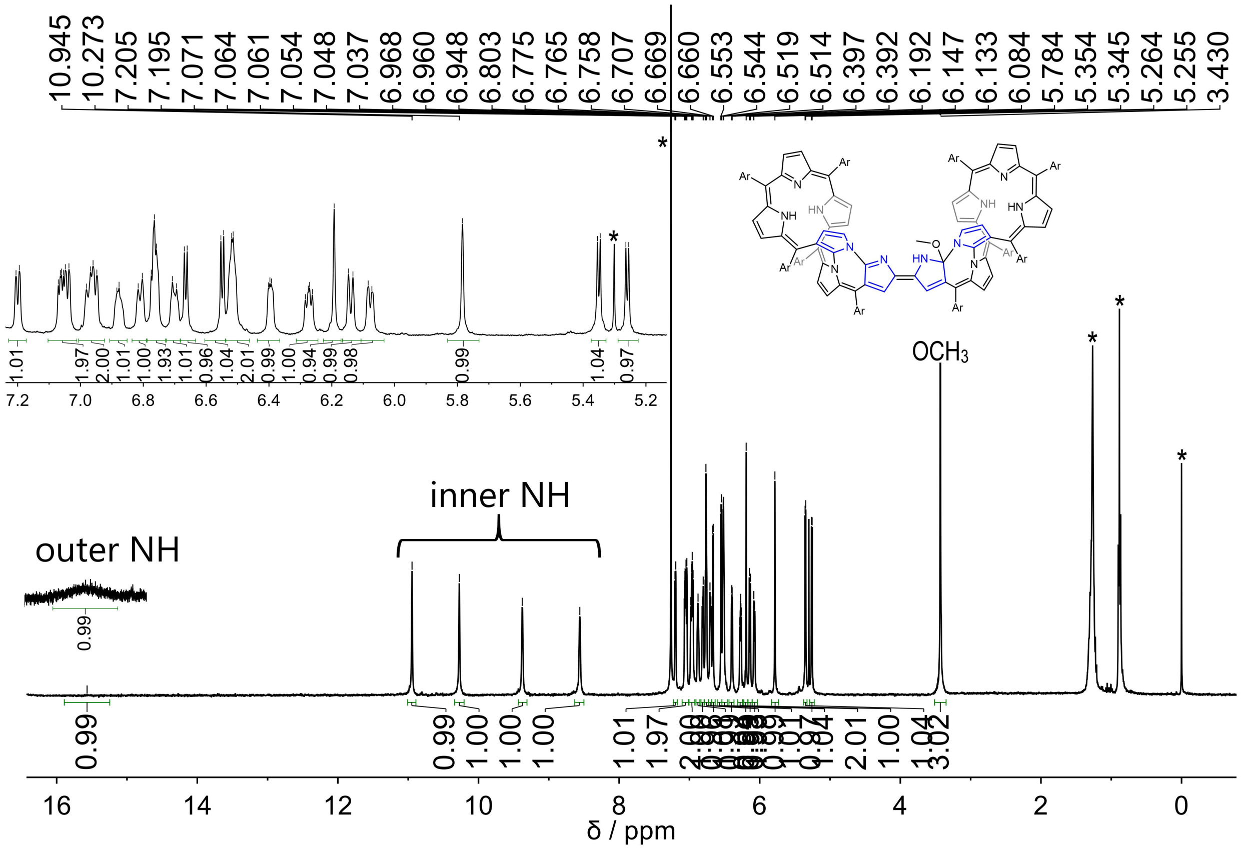

Supplementary Figure 6. ^{1}H NMR spectrum of **MD2** in $CDCl_3$ at 213 K.

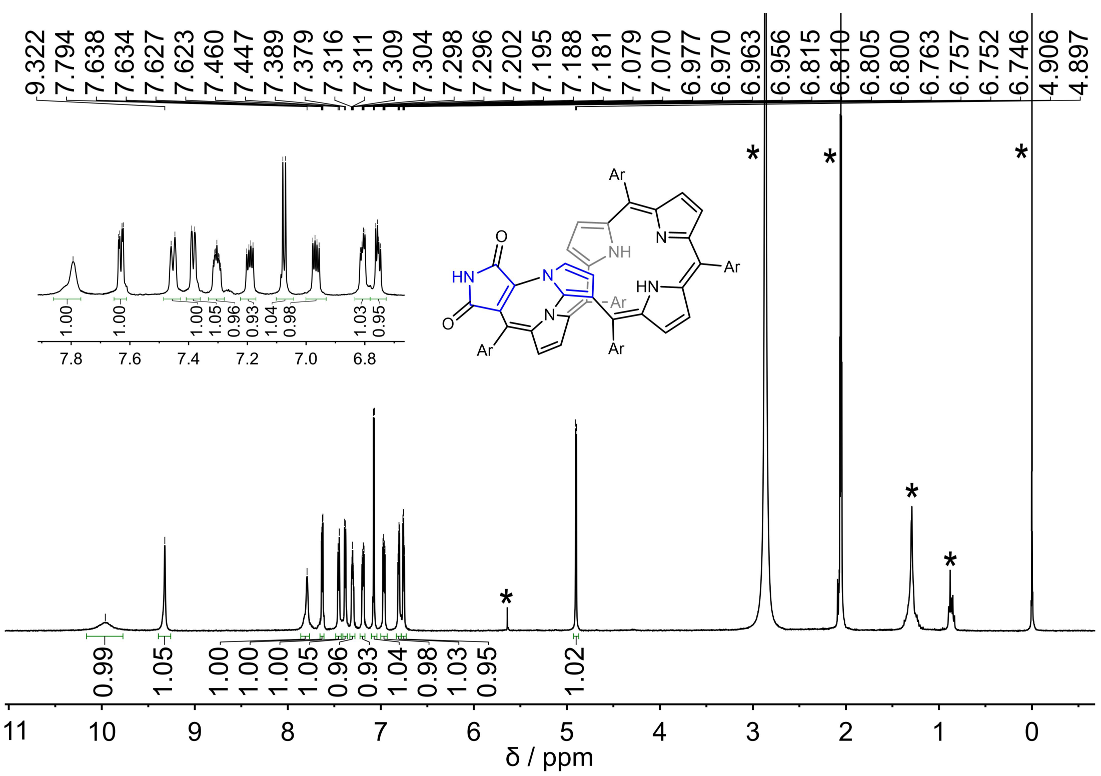

Supplementary Figure 7. ^{1}H NMR spectrum of **FHPO$_2$** in acetone-d_6.

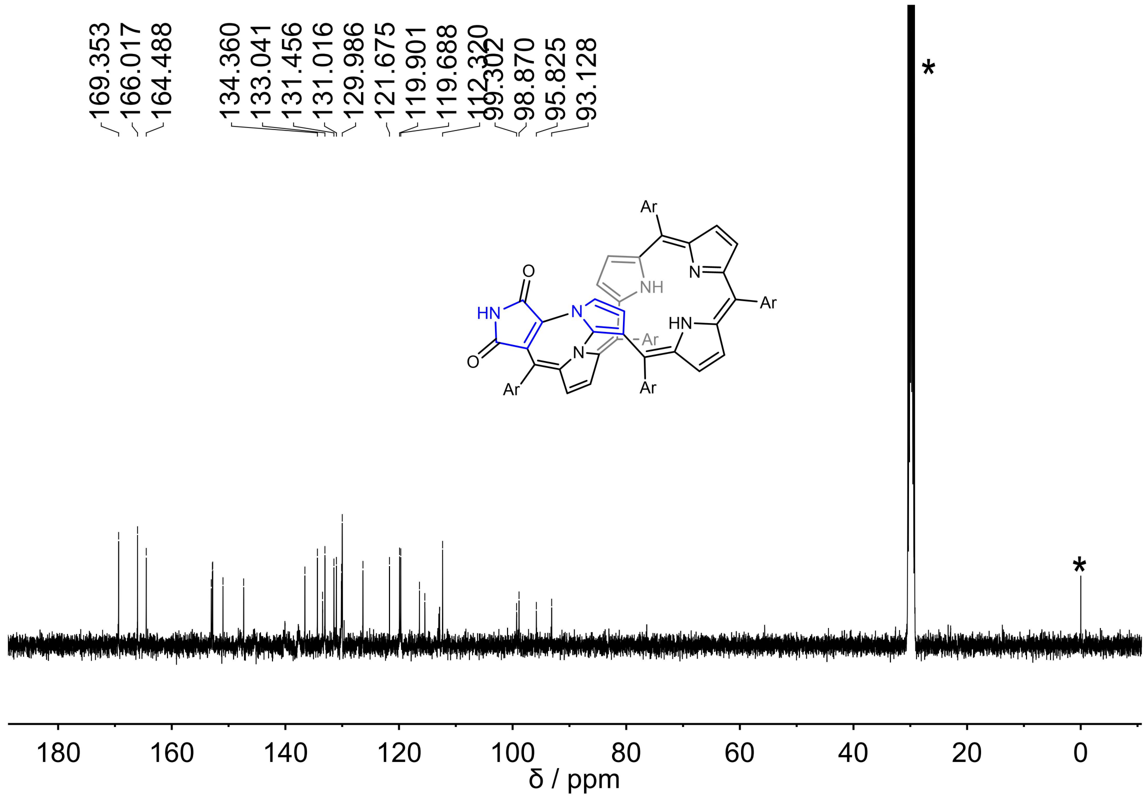

Supplementary Figure 8. ^{13}C NMR spectrum of **FHPO$_2$** in acetone-d_6.

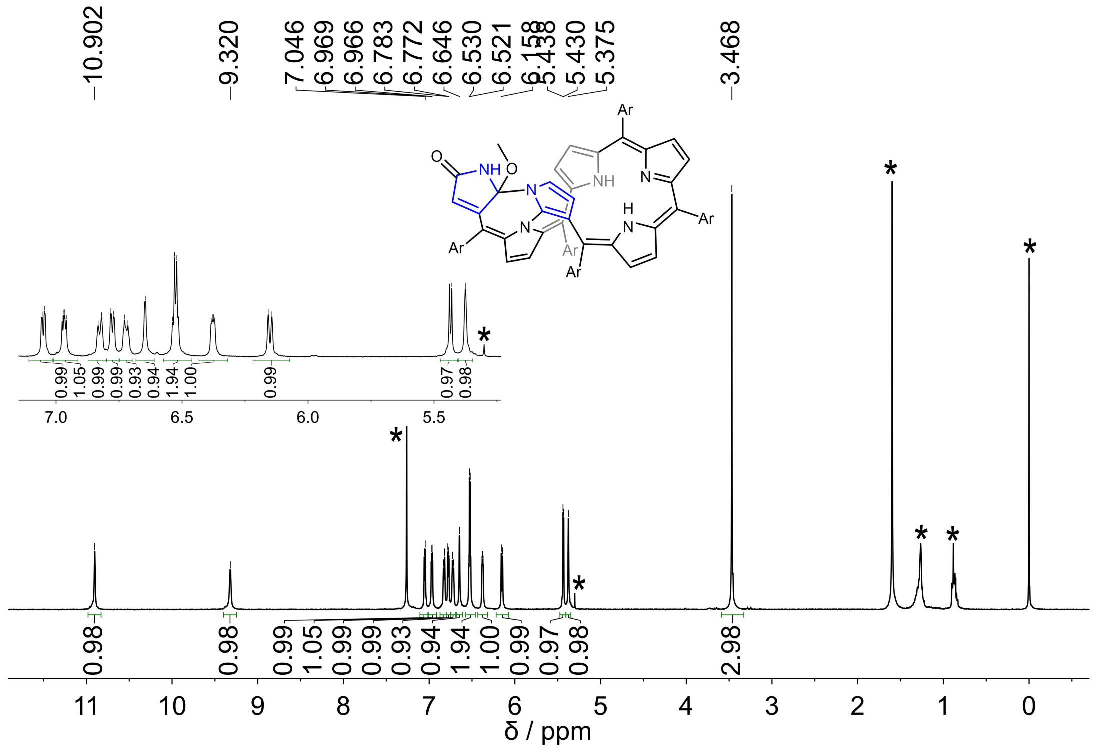

Supplementary Figure 9. ^{1}H NMR spectrum of **FHPMO** in $CDCl_3$.

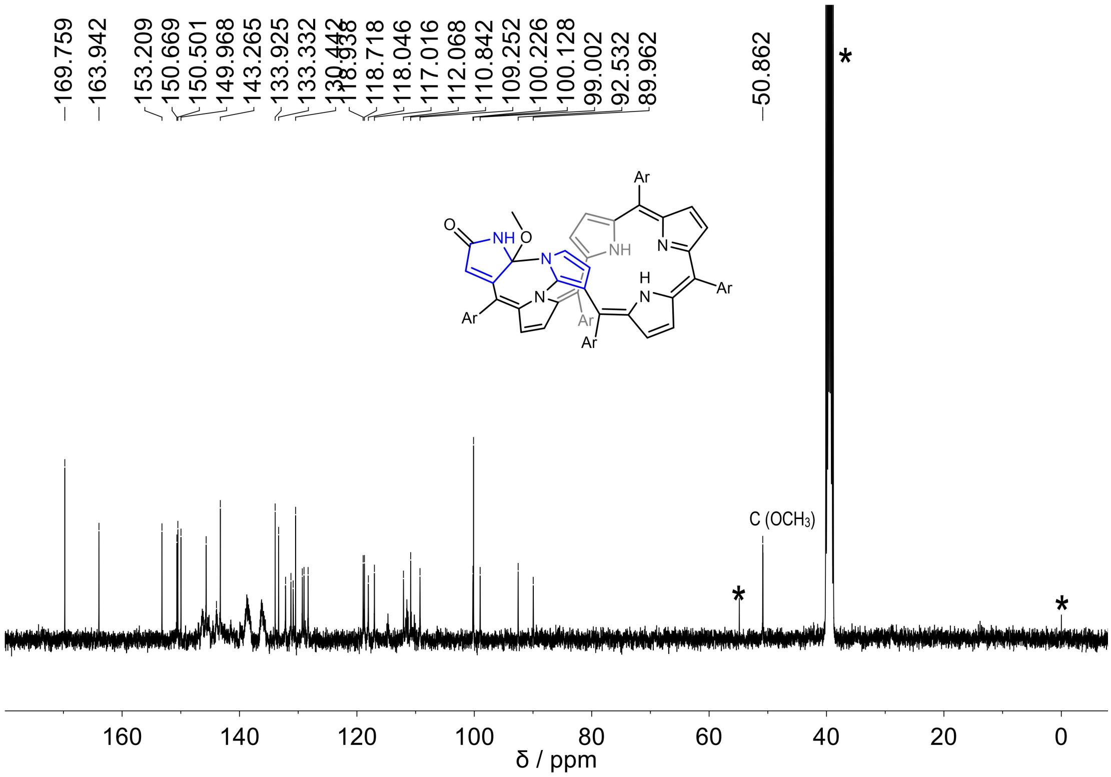

Supplementary Figure 10. ^{13}C NMR spectrum of **FHPMO** in DMSO-d_6.

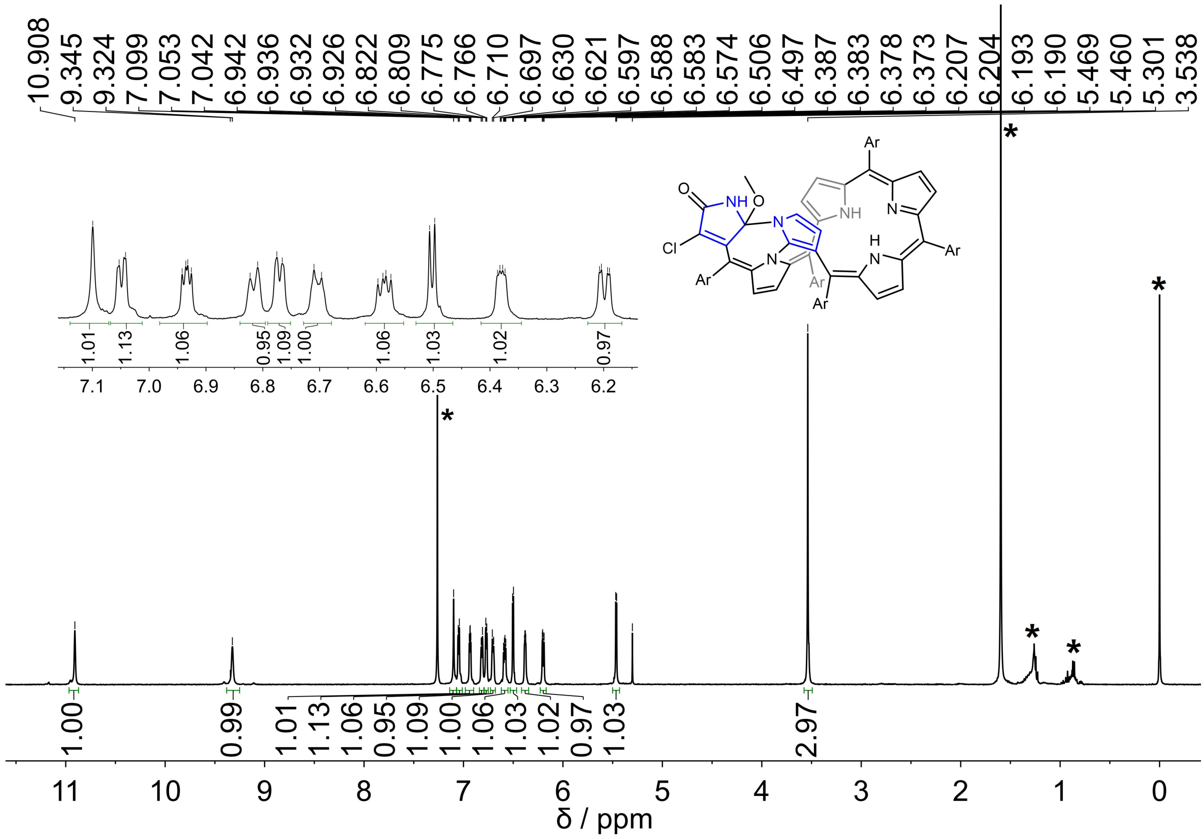

Supplementary Figure 11. ^{1}H NMR spectrum of **FHPMOCl** in $CDCl_3$.

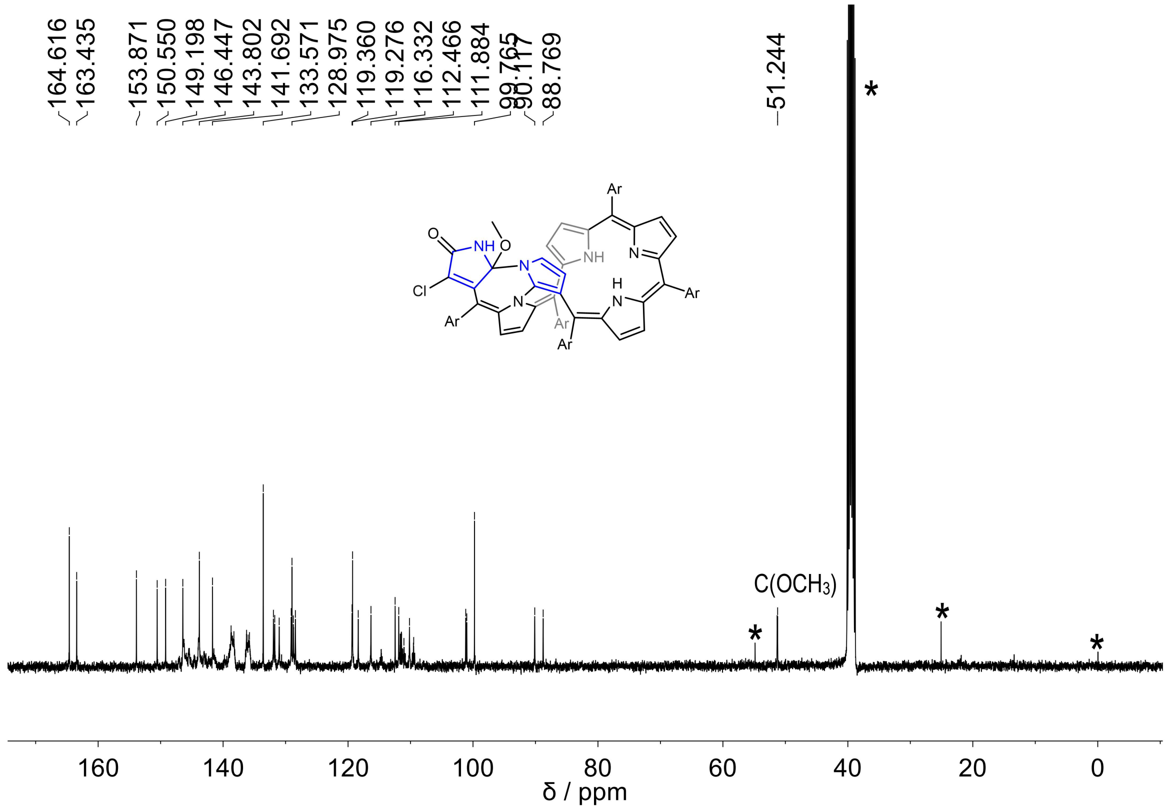

Supplementary Figure 12. ^{13}C NMR spectrum of **FHPMOCl** in DMSO-d_6.

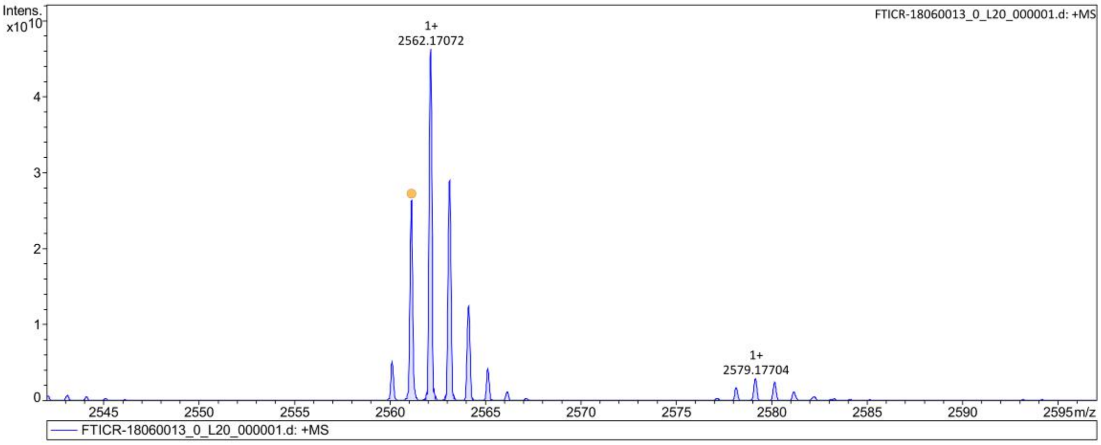

Meas. m/z	#	Ion Formula	Score	m/z	err [ppm]	Mean err [ppm]	mSigma	rdb	e⁻ Conf	N-Rule
2561.168280	1	C118H27F50N12	100.00	2561.167776	-0.2	1.6	75.4	87.0	even	ok

Supplementary Figure 13. HRMS of **D** recorded using a CH_2Cl_2 solution.

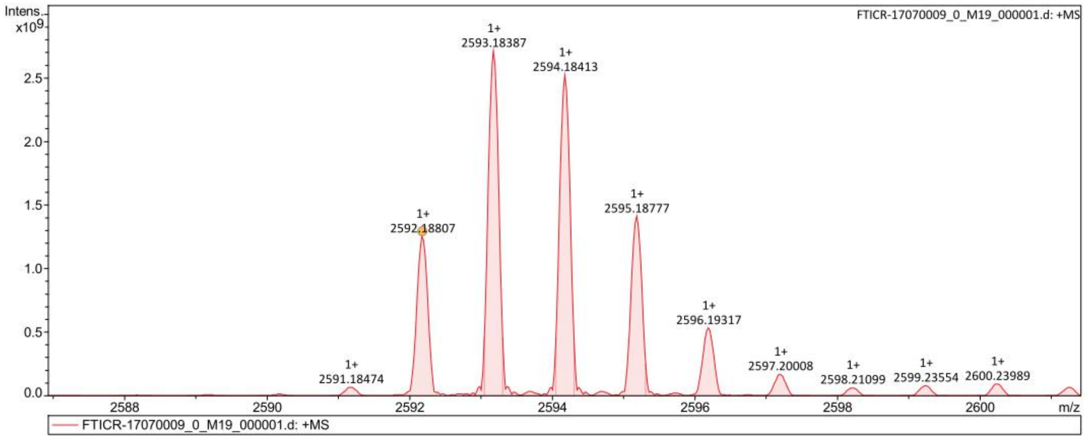

Meas. m/z	#	Ion Formula	Score	m/z	err [ppm]	Mean err [ppm]	mSigma	rdb	e⁻ Conf	N-Rule
2592.188066	1	C119H30F50N12O	100.00	2592.186166	-0.7	1.7	190.1	86.5	odd	ok

Supplementary Figure 14. HRMS of **MD1** recorded using a CH_2Cl_2 solution.

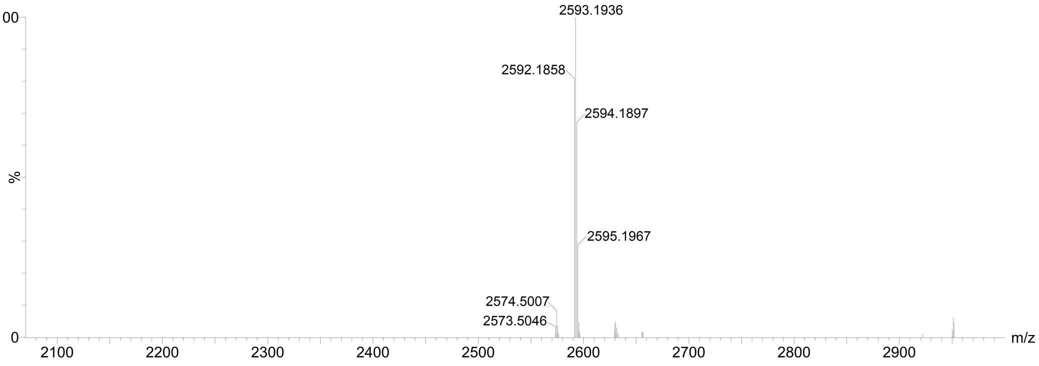

Supplementary Figure 15. HRMS of **MD2** recorded using a CH_2Cl_2 solution.

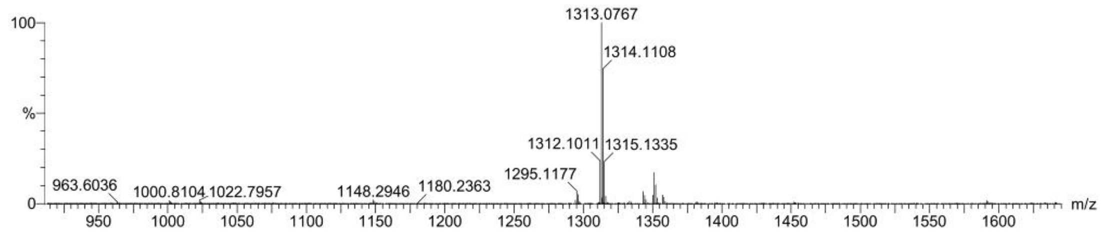

Minimum:				-1.5			
Maximum:		5.0	20.0	50.0			
Mass	Calc. Mass	mDa	PPM	DBE	i-FIT	i-FIT (Norm)	Formula
1313.0767	1313.0779	-1.2	-0.9	43.5	44.3	0.0	C59 H14 N6 O2 F25

Supplementary Figure 16. HRMS of **$FHPO_2$** recorded using a CH_2Cl_2 solution.

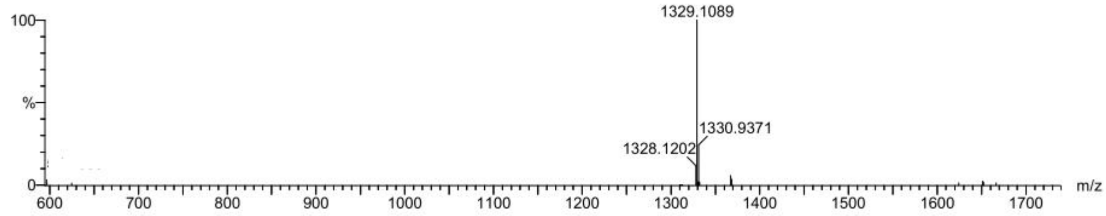

Minimum:				-1.5			
Maximum:		30.0	50.0	100.0			
Mass	Calc. Mass	mDa	PPM	DBE	i-FIT	i-FIT (Norm)	Formula
1329.1089	1329.1092	-0.3	-0.2	42.5	70.2	0.0	C60 H18 N6 O2 F25

Supplementary Figure 17. HRMS of **FHPMO** recorded using a CH_2Cl_2 solution.

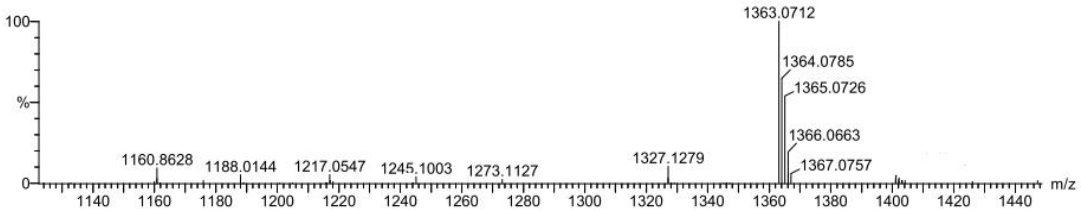

Minimum:				-1.5			
Maximum:		30.0	50.0	100.0			
Mass	Calc. Mass	mDa	PPM	DBE	i-FIT	i-FIT (Norm)	Formula
1363.0712	1363.0702	1.0	0.7	42.5	10.1	0.0	C60 H17 N6 O2 Cl F25

Supplementary Figure 18. HRMS of **FHPMOCl** recorded using a CH_2Cl_2 solution.

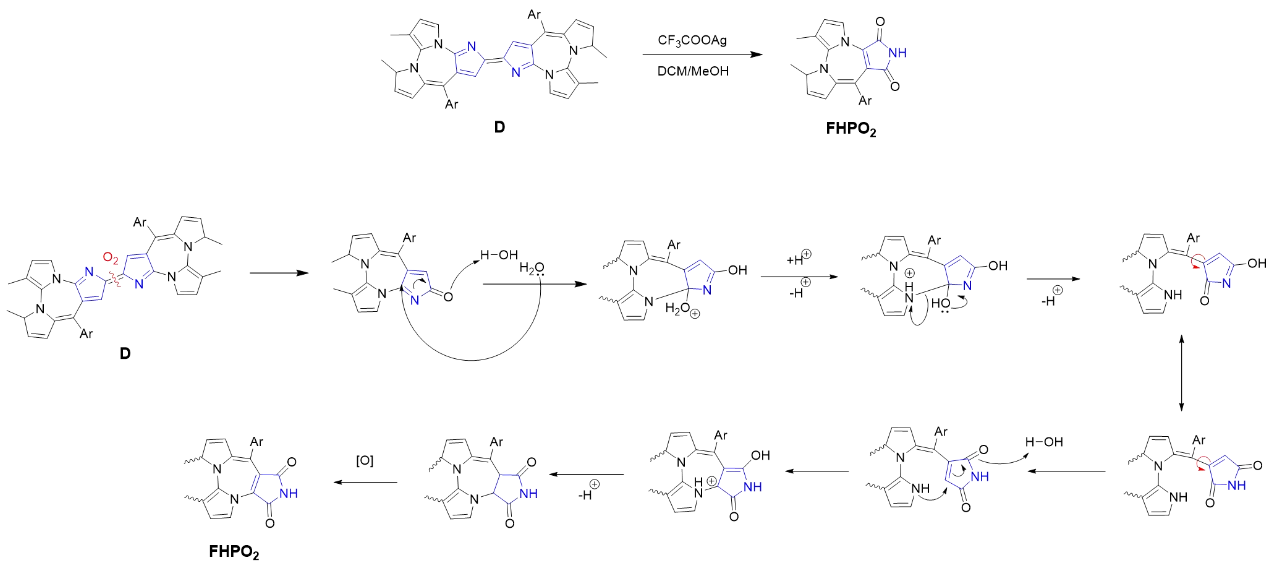

Supplementary Figure 19. A plausible mechanism for the formation of **FHPO₂** from **D** under oxidative conditions.

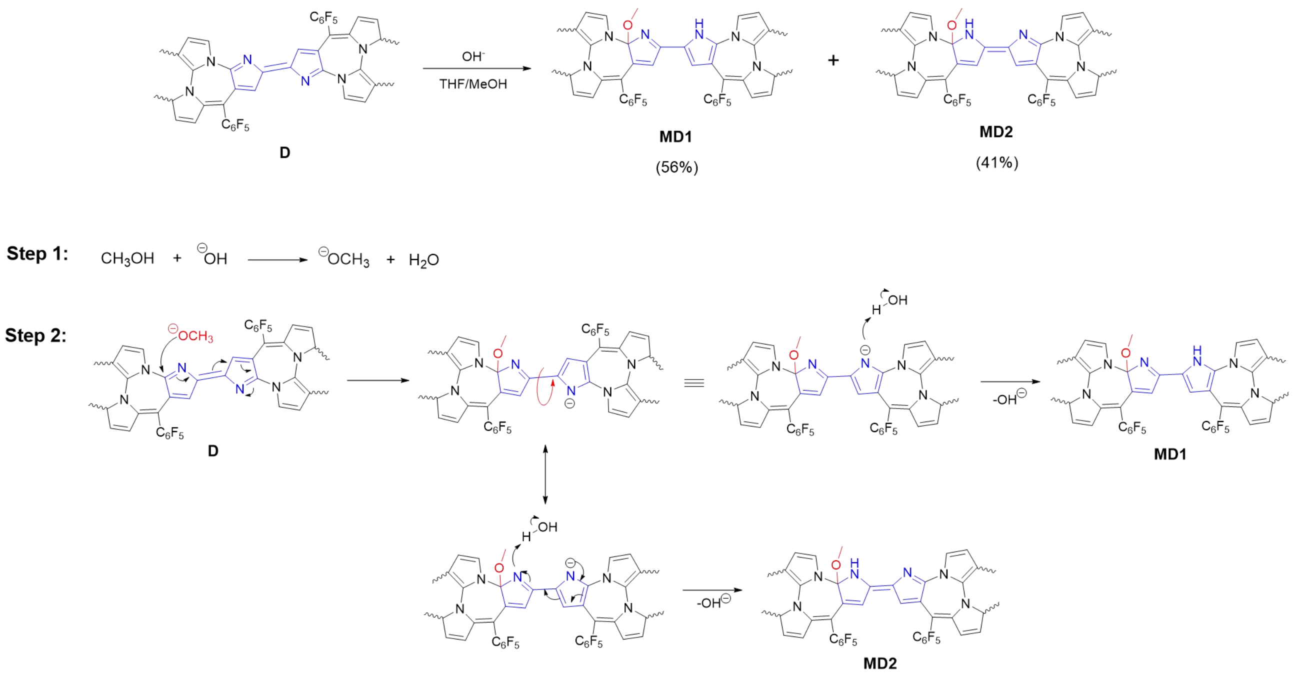

Supplementary Figure 20. A plausible mechanism for the formation of **MD1/2** from **D** under basic conditions.

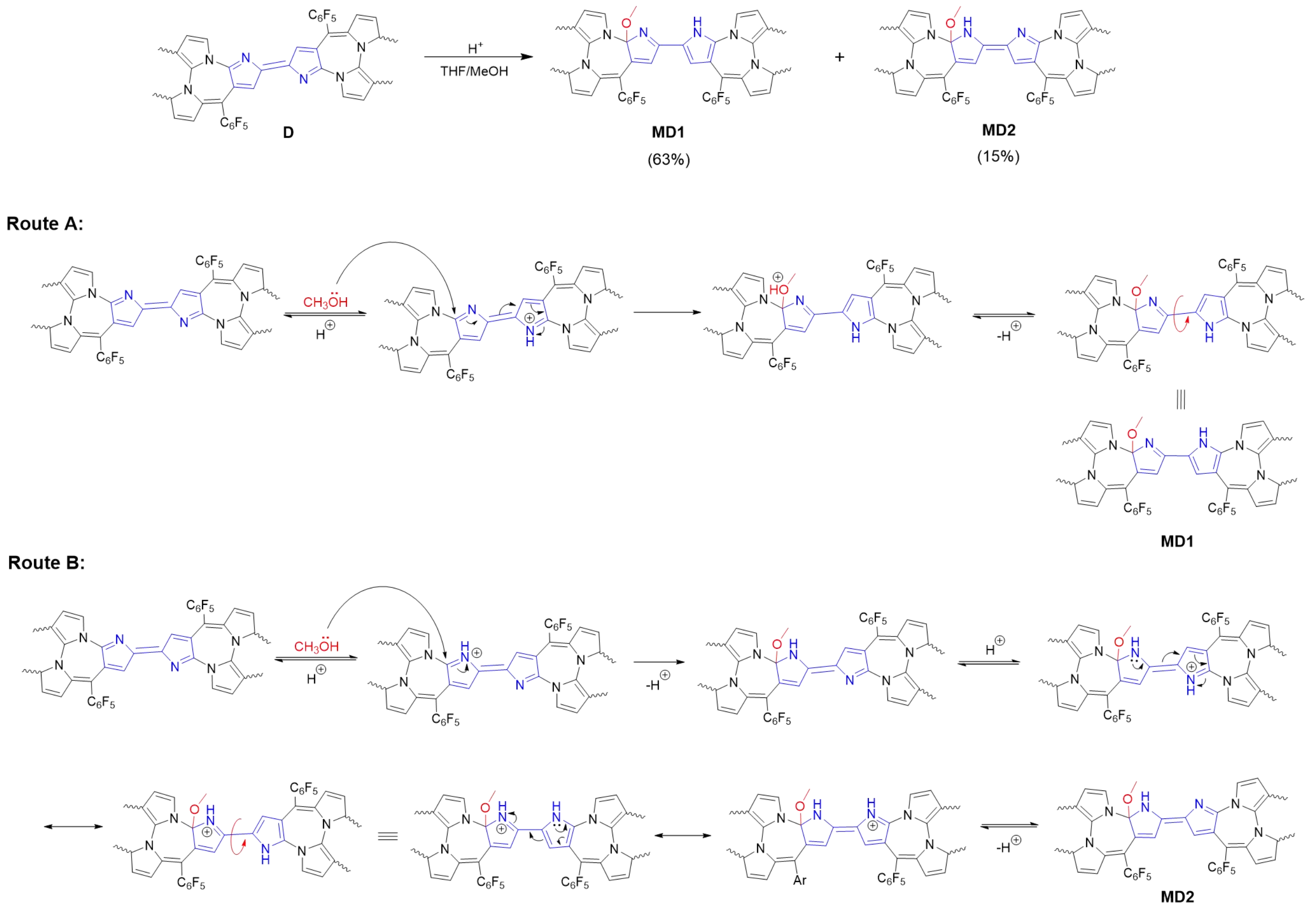

Supplementary Figure 21. A plausible mechanism for the formation of **MD1/2** from **D** under acid conditions

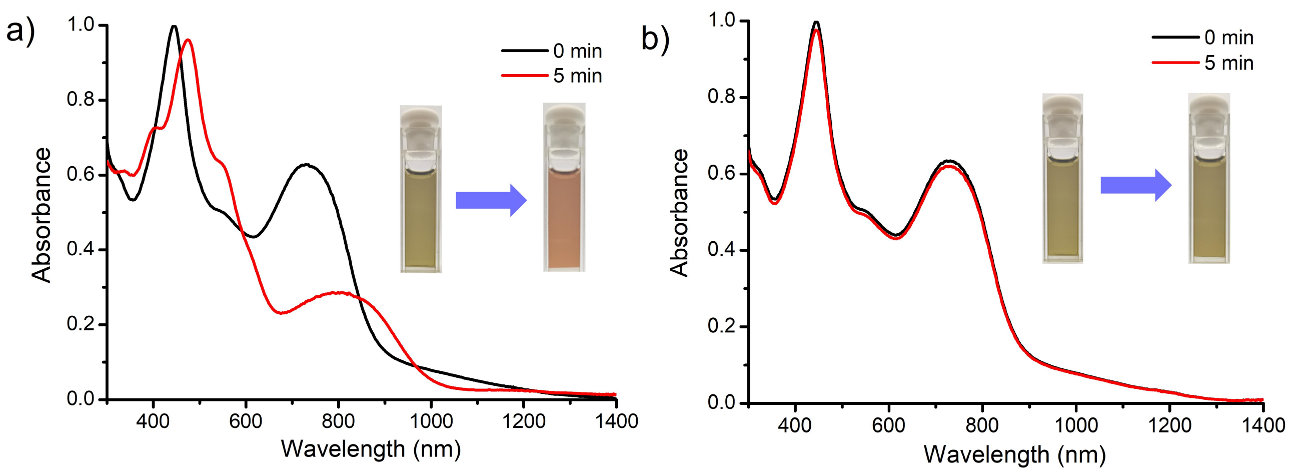

Supplementary Figure 22. Absorption spectral changes seen upon addition of a) methanol (3.0 μL, *ca.* 25 mM after dilution) or b) ethanol (3.0 μL, *ca.* 25 mM after dilution) into solutions of **D** (1.3×10^{-5} M) in THF (3.0 mL) in the presence of excess $NaOH_{(s)}$ (10 mg).

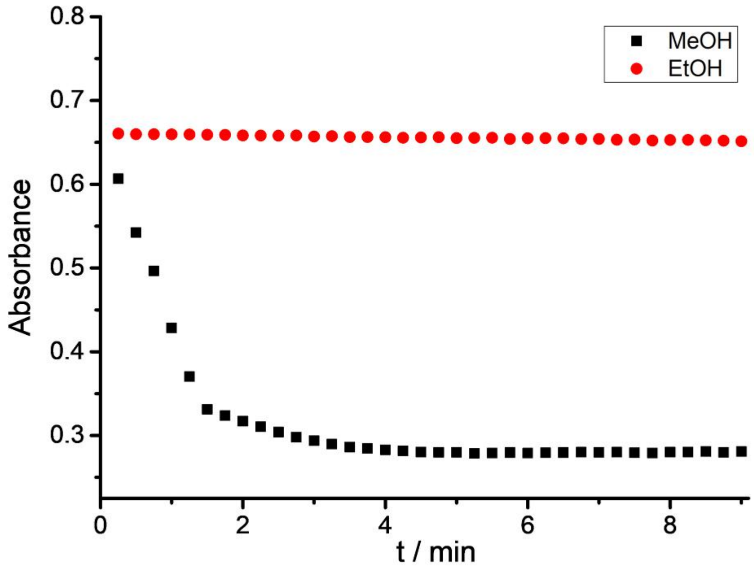

Supplementary Figure 23. Absorption changes at 734 nm seen upon the addition of methanol (3.0 μL, *ca.* 25 mM after dilution) or ethanol (3.0 μL, *ca.* 25 mM after dilution) into solutions of **D** (1.3×10^{-5} M) in THF (3.0 mL) in the presence of excess $NaOH_{(s)}$ (10 mg).

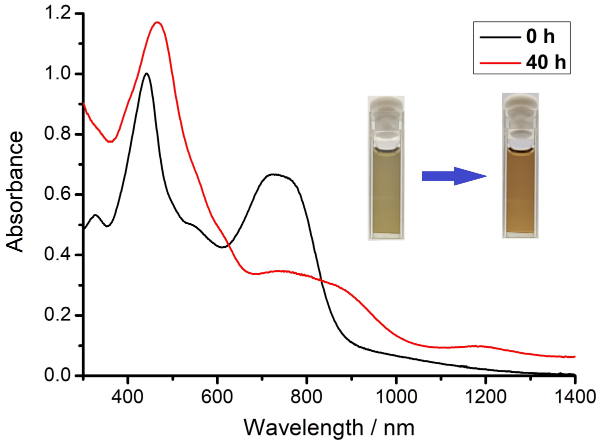

Supplementary Figure 24. Absorption spectral change seen upon the addition of methanol (0.50 μL, *ca.* 4.2 mM after dilution) into a solution of **D** (1.3×10^{-5} M) in THF (3.0 mL) in the presence of excess $NaOH_{(s)}$ (10 mg).

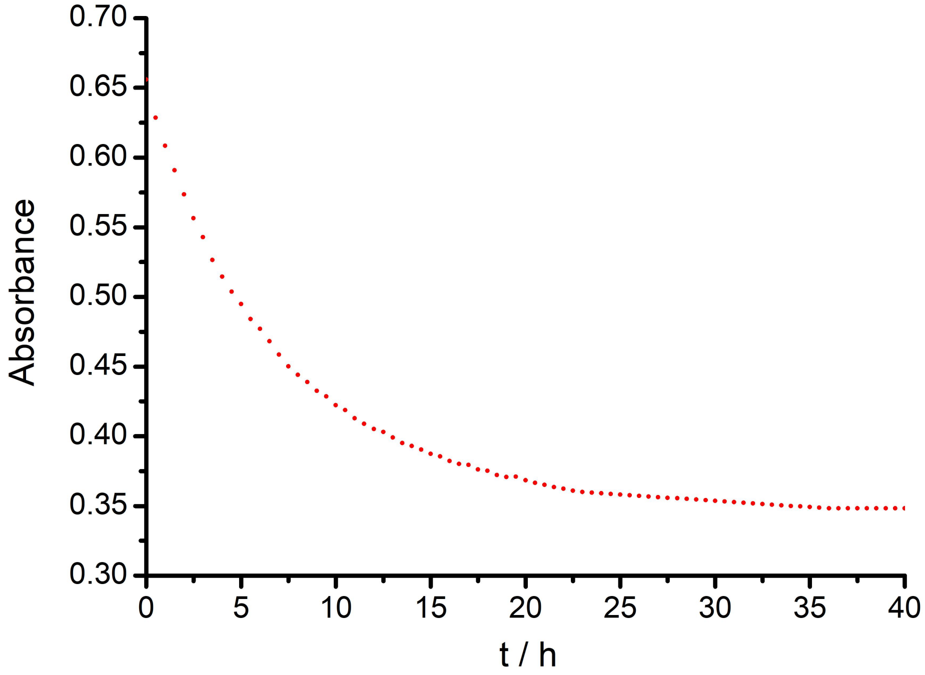

Supplementary Figure 25. Absorption changes at 734 nm seen upon the addition of methanol (0.50 μL, *ca.* 4.2 mM after dilution) into a solution of **D** (1.3×10^{-5} M) in THF (3.0 mL) in the presence of excess $NaOH_{(s)}$ (10 mg).

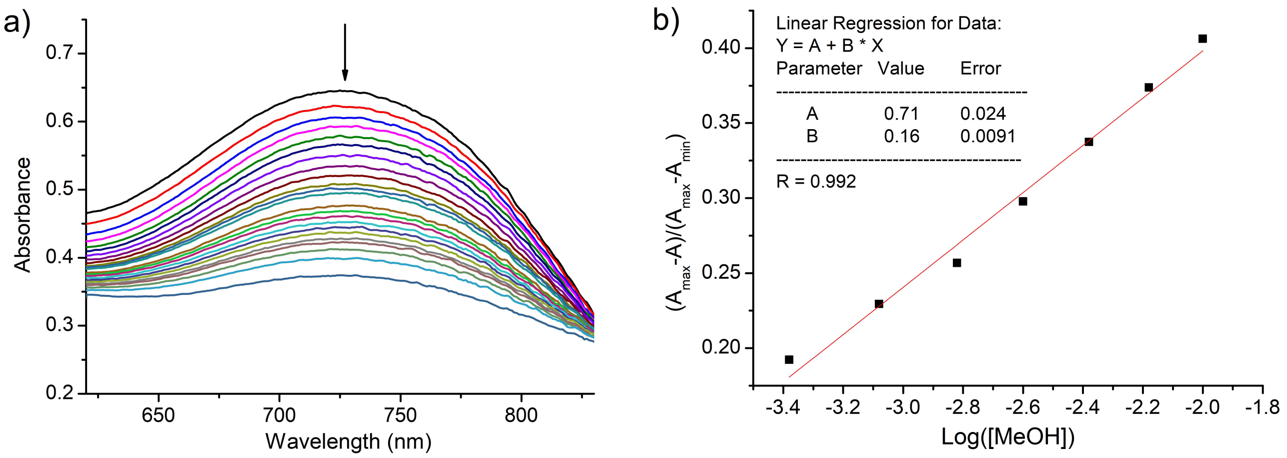

Supplementary Figure 26. a) Partial absorption changes seen upon the gradual addition of methanol into solutions of **D** (1.3×10^{-5} M) in THF (3.0 mL) in the presence of excess $NaOH_{(s)}$ (10 mg) for 24 h. b) Plot of $(A_{max}\text{-}A)/(A_{max}\ \text{-}A_{min})$ *vs.* Log([MeOH]); on this basis the calculated detection limit for MeOH in THF was determined to be *ca.* 3.7×10^{-5} M.

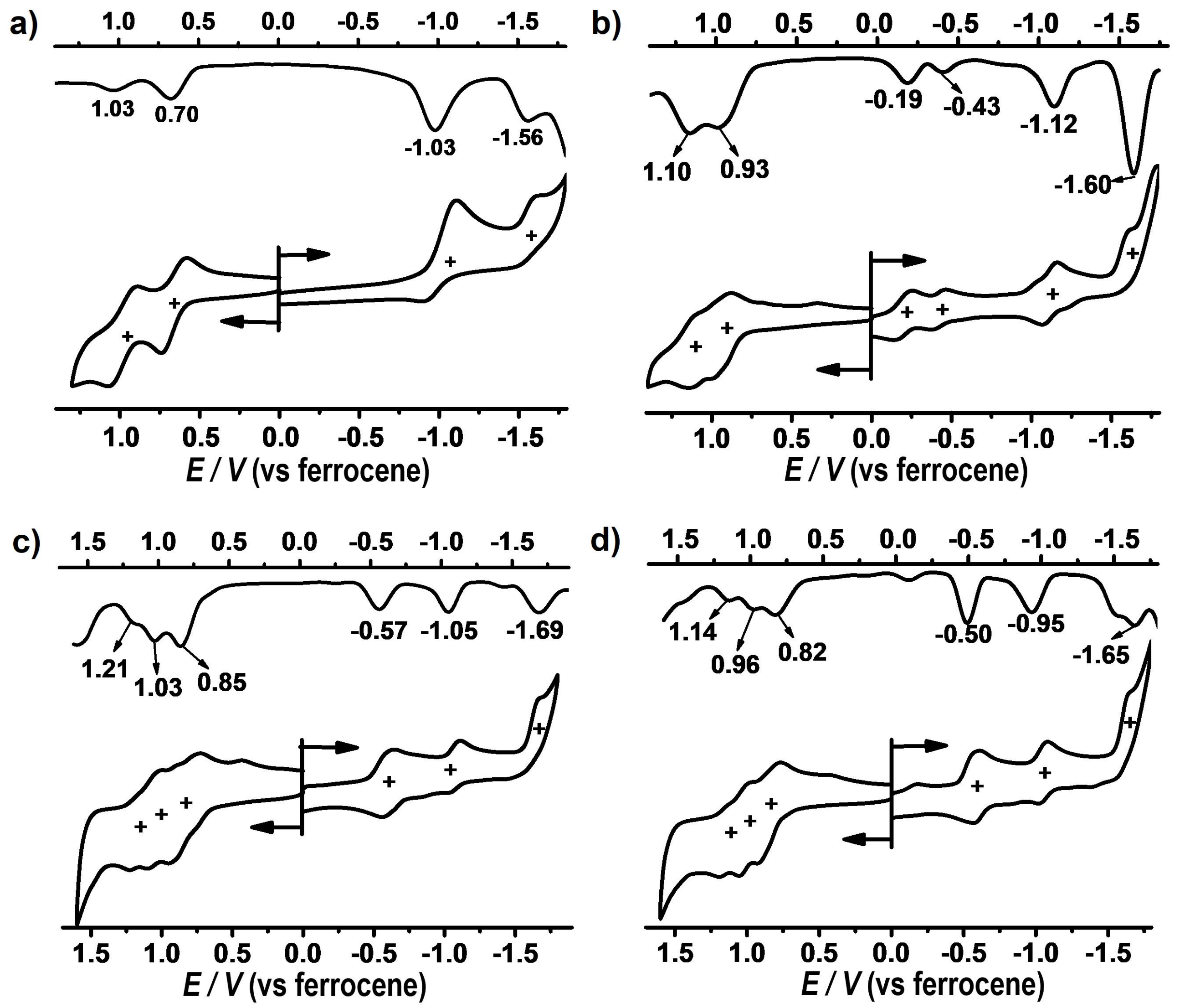

Supplementary Figure 27. Cyclic voltammograms (CV) and differential pulse voltammetry (DPV) traces for a) **FHP**, b) **D**, c) **MD1**, and d) **MD2** in CH_2Cl_2 containing 0.1 M $TBAClO_4$ at a scan rate of 0.10 V/s.

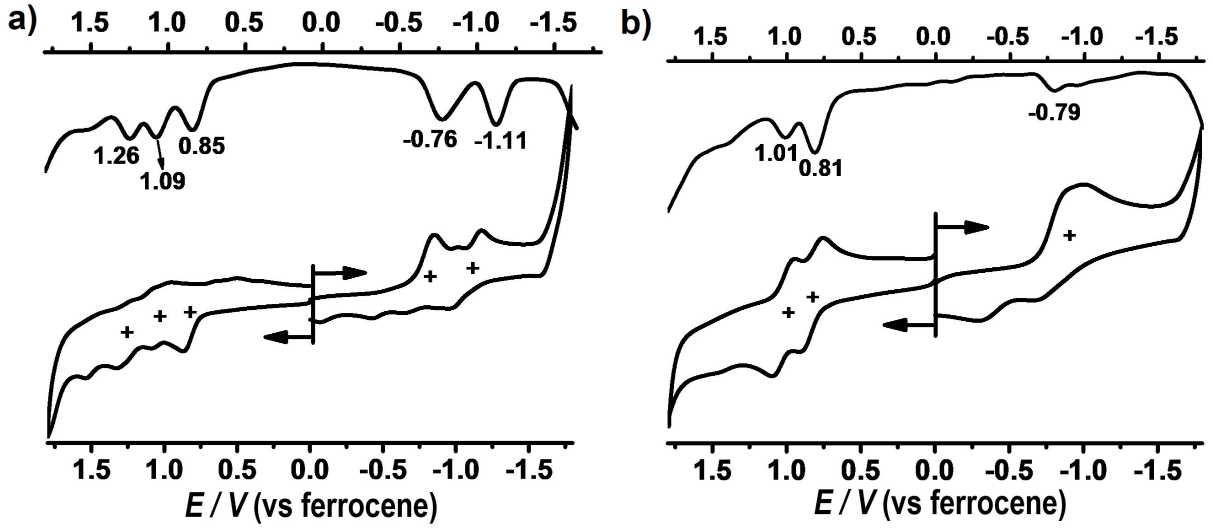

Supplementary Figure 28. CV and DPV traces for a) **FHP**, b) $\mathbf{FHPO_2}$, and c) **FHPMO** in CH_2Cl_2 containing 0.1 M $TBAClO_4$ at a scan rate of 0.10 V/s.

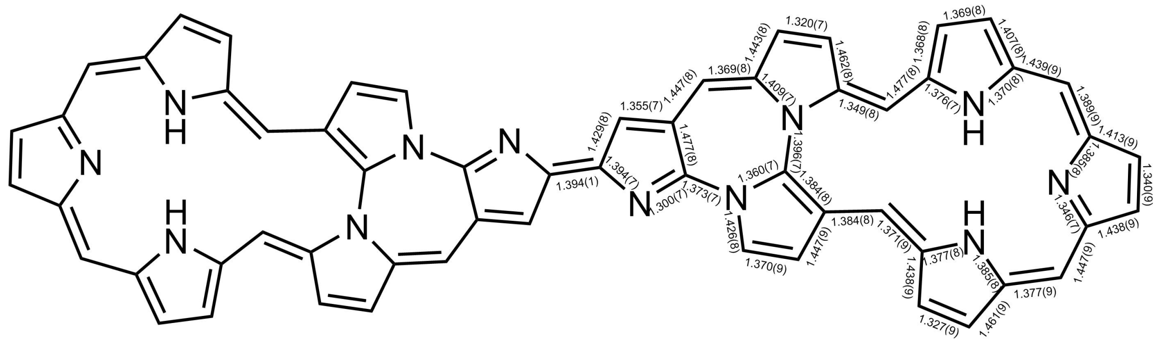

Supplementary Figure 29. Selected bond lengths (Å) for the crystal structure of **D**.

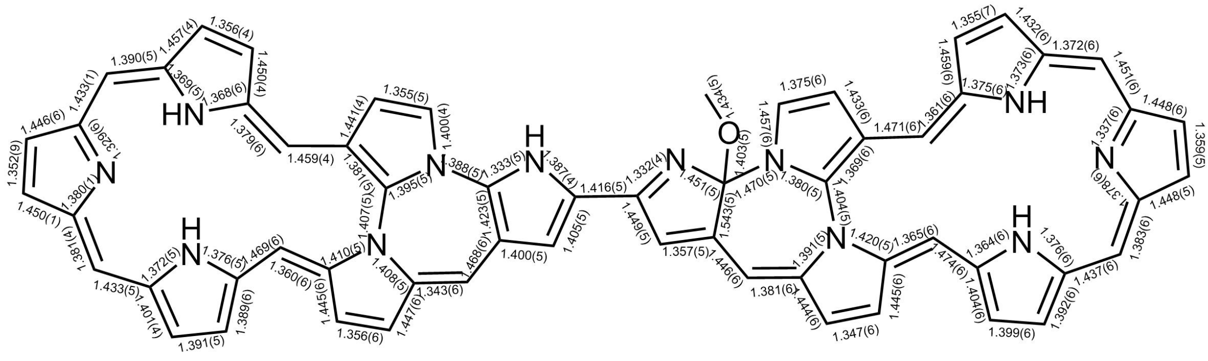

Supplementary Figure 30. Selected bond lengths (Å) for the crystal structure of **MD1**.

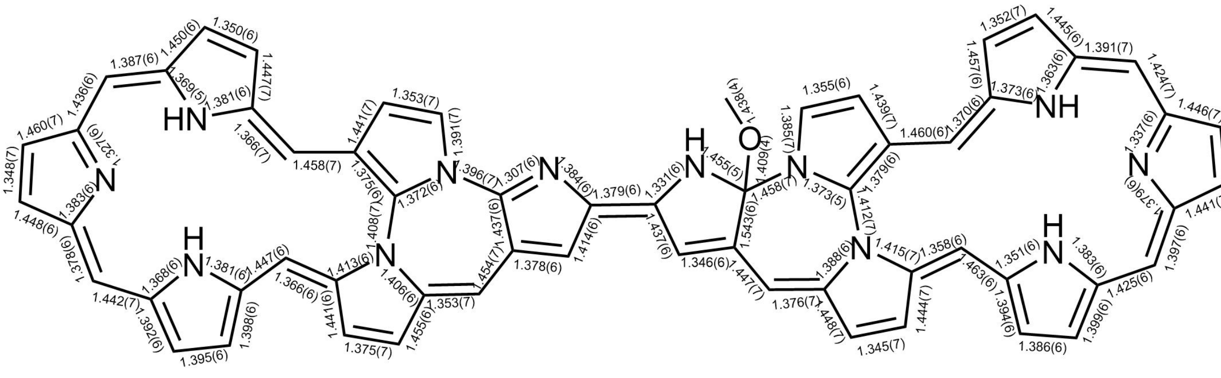

Supplementary Figure 31. Selected bond lengths (Å) for the crystal structure of **MD2**.

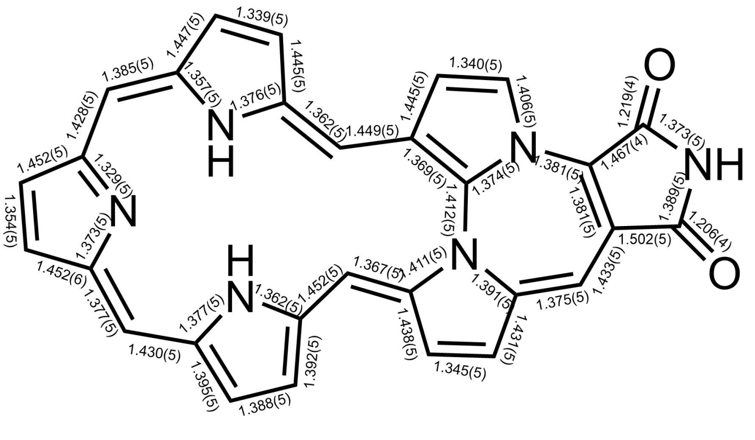

Supplementary Figure 32. Selected bond lengths (Å) for the crystal structures of **FHPO$_2$**.

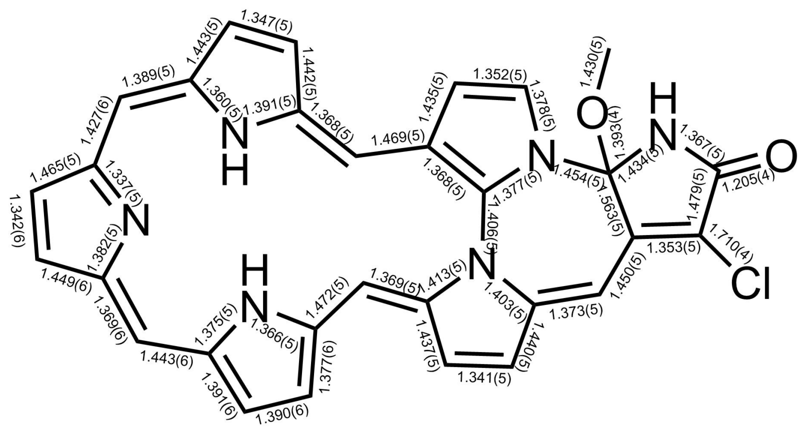

Supplementary Figure 33. Selected bond lengths (Å) for the crystal structures of **FHPMOCl**.

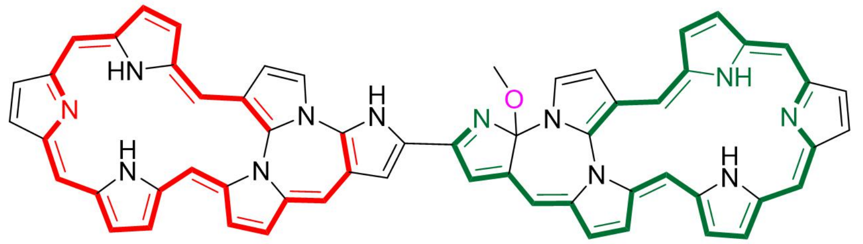

MD1

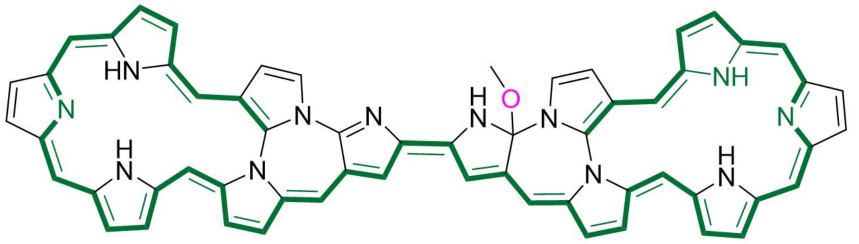

MD2

Supplementary Figure 34. Proposed conjugation pathways within **MD1** and **MD2** (pentafluorophenyl substituents have been omitted for clarity)

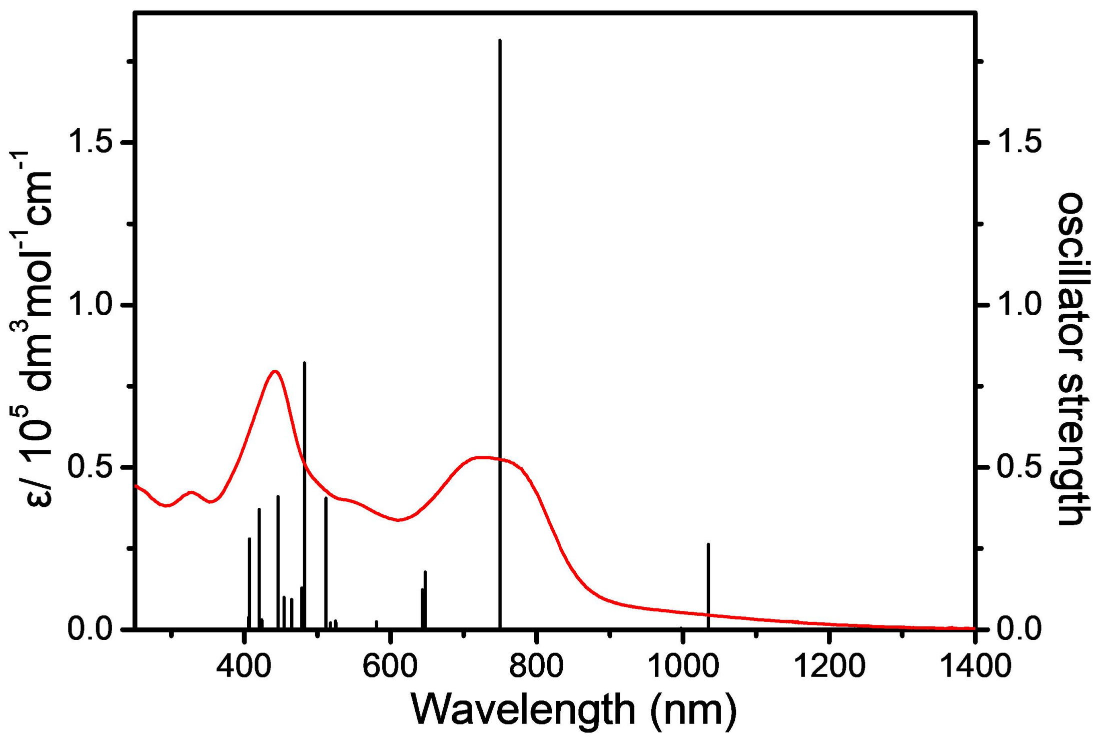

Supplementary Figure 35. TD-DFT-computed vertical energies (bar) of **D** along with the experimental spectra (lines).

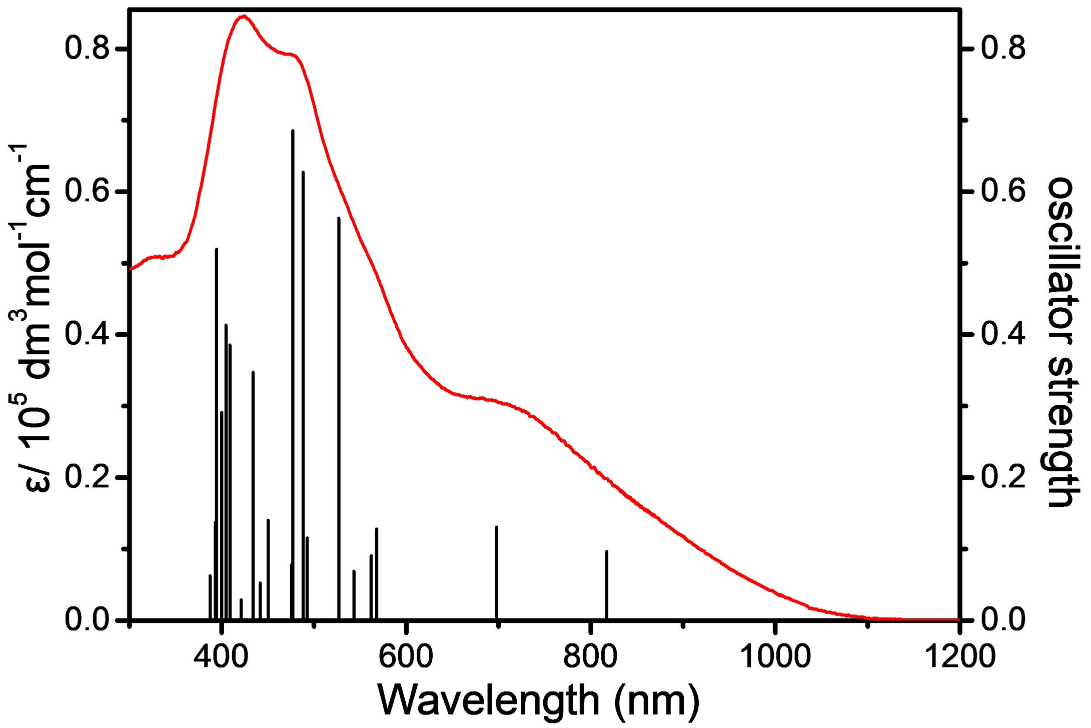

Supplementary Figure 36. TD-DFT-computed vertical energies (bar) of **MD1** along with the experimental spectra (lines).

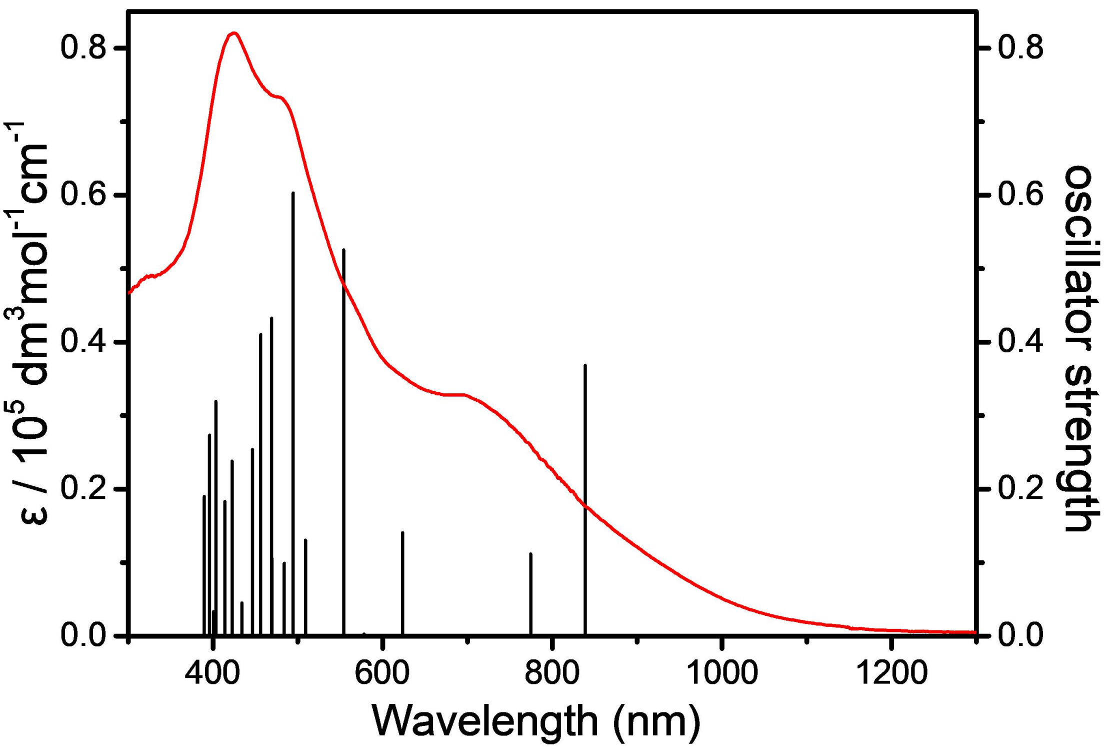

Supplementary Figure 37. TD-DFT-computed vertical energies (bar) of **MD2** along with the experimental spectra (lines).

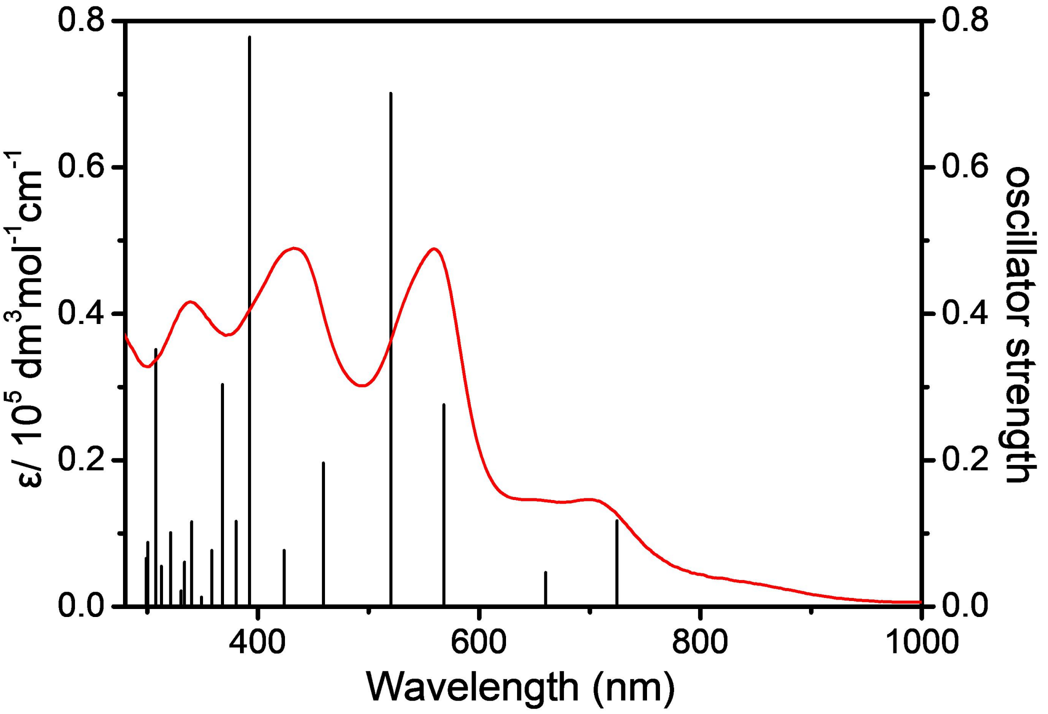

Supplementary Figure 38. TD-DFT-computed vertical energies (bar) of **FHPO$_2$** along with the experimental spectra (lines).

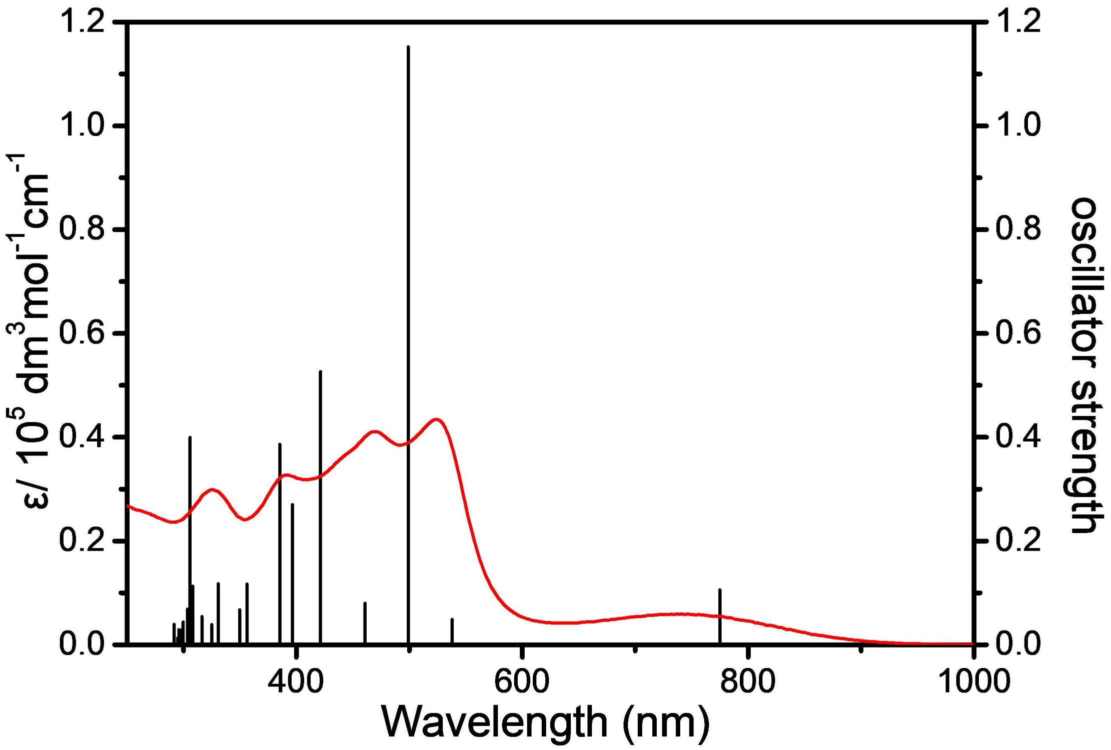

Supplementary Figure 39. TD-DFT-computed vertical energies (bar) of **FHPMO** along with the experimental spectra (lines).

Supplementary Tables

Supplementary Table 1. Electrochemical data for the dimers and monomers considered in this study. [a] Oxidation and reduction potentials (V) were determined using differential pulse voltammetry (DPV) in CH_2Cl_2 containing 0.1 M $TBAClO_4$ as the supporting electrolyte (vs. Ag/AgCl). The potentials were calibrated to the ferrocenium/ferrocene (Fc^+/Fc) couple. [b] Energy gap ($\Delta E = e(E_{ox(1/2)} - E_{red(1/2)})$).

Compounds[a]	$E^{1/2}_{ox1}$	$E^{1/2}_{ox2}$	$E^{1/2}_{ox3}$	$E^{1/2}_{red1}$	$E^{1/2}_{red2}$	$E^{1/2}_{red3}$	$E^{1/2}_{red4}$	ΔE[b]
**FHP**	0.70	1.03	-	-1.03	-1.56	-	-	1.73
**D**	0.93	1.10	-	-0.19	-0.43	-1.12	-1.60	1.12
**MD1**	0.85	1.03	1.21	-0.57	-1.05	-1.69	-	1.42
**MD2**	0.82	0.96	1.14	-0.50	-0.95	-1.65	-	1.32
**$FHPO_2$**	0.85	1.09	1.26	-0.76	-1.11	-	-	1.61
**FHPMO**	0.81	1.01	-	-0.79	-	-	-	1.60

Supplementary Table 2. Crystal Data and Structure Refinements for **D** and **MD1/2**.

Compounds	**D**	**MD1**	**MD2**
Formula	$C_{118}H_{26}F_{50}N_{12}$	$C_{119}H_{30}F_{50}N_{12}O$	$C_{121}H_{33}F_{50}N_{14}O$
Formula weight (g/mol)	2561.51	2593.55	2648.61
Crystal system	tetragonal	orthorhombic	orthorhombic
Temperature (K)	173	120	100
Crystal size (mm^3)	0.30 × 0.18 × 0.15	0.31 × 0.14 × 0.10	0.28 × 0.27 × 0.25
Theta range for data collection (°)	2.73–19.6	2.13–22.7	2.93–75.7
Space group	*P*4/*nnc*	*Pbca*	*Pbca*
a (Å)	25.4826 (11)	23.4847 (7)	13.40730(10)
b (Å)	25.4826(11)	22.3149 (7)	38.9134(3)
c (Å)	47.075(4)	48.5431 (16)	44.9998(3)
α (°)	90.00	90.00	90.00
β (°)	90.00	90.00	90.00
γ (°)	90.00	90.00	90.00
Volume ($Å^3$)	30569(4)	25439.4 (14)	23477.5(3)
Z	8	8	8
ρ_{calc} (g/cm^3)	1.113	1.354	1.499
F(000)	10144	10288	10520
(mm^{-1})	0.11	1.20	1.31
Index ranges	$-24 \leq h \leq 21$<br>$-17 \leq k \leq 25$<br>$-47 \leq l \leq 39$	$-26 \leq h \leq 26$<br>$-21 \leq k \leq 25$<br>$-55 \leq l \leq 51$	$-16 \leq h \leq 15$<br>$-46 \leq k \leq 48$<br>$-50 \leq l \leq 56$
R_1 [I > 2σ(I)]	0.068	0.056	0.077
wR_2 (all data)	0.235	0.155	0.208
GOF	1.03	1.00	1.14
Largest diff. peak / hole (e $Å^{-3}$)	0.23 and -0.25	0.45 and -0.27	0.85 and -0.30
Reflections collected/Unique	49937/8080	127594/20189	152189/24066
CCDC number	1886416	1886417	1985682

Supplementary Table 3. Crystal Data and Structure Refinements for **FHPO₂** and **FHPMOCl**

Compounds	**$FHPO_2$**	**FHPMOCl**
Formula	$C_{59}H_{13}F_{25}N_6O_2$	$C_{60}H_{16}ClF_{25}N_6O_2$
Formula weight (g/mol)	1312.75	1420.35
Crystal system	triclinic	triclinic
Temperature (K)	100	173
Crystal size (mm^3)	0.07 × 0.05 × 0.03	0.41 × 0.33 × 0.13
Theta range for data collection (°)	1.82–31.2	2.89–24.9
Space group	*P-1*	*P-1*
a (Å)	13.9507 (6)	11.7231 (6)
b (Å)	14.4984(5)	16.5466 (9)
c (Å)	16.4369(7)	18.0585 (9)
α (°)	99.697(3)	68.627 (1)
β (°)	107.967(4)	86.037 (2)
γ (°)	116.757(4)	79.301 (2)
Volume ($Å^3$)	2632.5(2)	30569(4)
Z	2	2
ρ_{calc} (g/cm^3)	1.656	1.472
F(000)	1300	1418
(mm^{-1})	0.16	0.18
Index ranges	$-20 \leq h \leq 20$<br>$-20 \leq k \leq 19$<br>$-22 \leq l \leq 23$	$-14 \leq h \leq 14$<br>$-19 \leq k \leq 19$<br>$-21 \leq l \leq 21$
R_1 [$I > 2\sigma(I)$]	0.079	0.068
wR_2 (all data)	0.180	0.186
GOF	1.01	1.01
Largest diff. peak / hole (e $Å^{-3}$)	0.41 and -0.36	0.60 and -0.33
Reflections collected/Unique	51211/15765	33848/11713
CCDC number	1985685	1886415

Supplementary Table 4. Frontier molecular orbital energies for the monomers of this study calculated using the B3LYP/6-31G(d) method[1-3].

Compounds	E_{HOMO} (eV)	E_{LUMO} (eV)	H-L gap (eV)
**$FHPO_2$**	-5.07	-3.13	1.94
**FHPMO**	-4.90	-3.08	1.82

Supplementary Table 5. Relative energies of **D/D-a** based on different basis sets[3-7].

Basis sets	Energy	**D**	**D-a**
B3LYP/6-31G(d)	E_{total} (a.u.)	-10160.5089148	-10160.5023408
	$E_{relative}$ (kJ/mol)	0	+17.3
BMK/6-31G(d)	E_{total} (a.u.)	-10155.7915451	-10155.7848454
	$E_{relative}$ (kJ/mol)	0	+17.6
GD3-B3LYP/6-31G(d)	E_{total} (a.u.)	-10160.8591798	-10160.8469248
	$E_{relative}$ (kJ/mol)	0	+33.1
B3LYP/6-31G(d,p)	E_{total} (a.u.)	-10160.5607041	-10160.5541587
	$E_{relative}$ (kJ/mol)	0	+17.2
B3LYP/6-311G(d,p)	E_{total} (a.u.)	-10163.2296804	-10163.2226136
	$E_{relative}$ (kJ/mol)	0	+18.6

Supplementary Table 6. Relative energies of **MD1/MD1-a/MD2/MD2-a** based on different basis sets[3-7].

Basis sets	Energy	**MD1**	**MD1-a**	**MD2**	**MD2-a**
B3LYP/6-31G(d)	E_{total} (a.u.)	-10276.248242	-10276.2389056	-10276.2474399	-10276.2438643
	$E_{relative}$ (kJ/mol)	0	+24.5	+2.1	+11.4
BMK/6-31G(d)	E_{total} (a.u.)	-10271.4743429	-10271.4687388	-10271.4741846	-10271.4703833
	$E_{relative}$ (kJ/mol)	0	+14.7	+0.4	+10.4
GD3-B3LYP/6-31G(d)	E_{total} (a.u.)	-10276.6158937	-10276.6032838	-10276.6154409	-10276.6057217
	$E_{relative}$ (kJ/mol)	0	+33.1	+1.2	+26.7
B3LYP/6-31G(d,p)	E_{total} (a.u.)	-10276.3077245	-10276.2983779	-10276.3069734	-10276.3033013
	$E_{relative}$ (kJ/mol)	0	+24.5	+2.0	+11.6
B3LYP/6-311G(d,p)	E_{total} (a.u.)	-10279.0088403	-10278.9984074	-10279.0055516	-10279.0050695
	$E_{relative}$ (kJ/mol)	0	+27.4	+8.6	+9.9

Supplementary Table 7. The energy of the S_1 states (nm) for dimers **D**, **MD1**, **MD2** calculated by means of different functionals using 6-31G(d) basis set and the PCM model (DCM as the solvent)[1, 7-11]. Oscillator strengths are presented in the parentheses below.

Compounds	B3LYP	PBE0	ω-B97XD	M062X	CAM-B3LYP	BMK	Exp.
**D**	1464 (0.072)	1328 (0.102)	802 (0.789)	904 (0.413)	854 (0.575)	1034 (0.263)	–[a]
**MD1**	968 (0.074)	757 (0.109)	742 (0.112)	771 (0.102)	920 (0.08)	817 (0.097)	–[b]
**MD2**	1057 (0.079)	956 (0.313)	743 (0.386)	784 (0.381)	764 (0.399)	838 (0.368)	–[c]

[a] structureless absorption in the range *ca.*1000-1300 nm

[b] structureless absorption in the range *ca.* 800-1000 nm

[c] structureless absorption in the range *ca.* 800-1200 nm

Supplementary Table 8. Selected molecular orbitals of **FHPO₂** and **FHPMO** as calculated using the B3LYP/6-31G(d) method.

	**$FHPO_2$**	**FHPMO**
HOMO	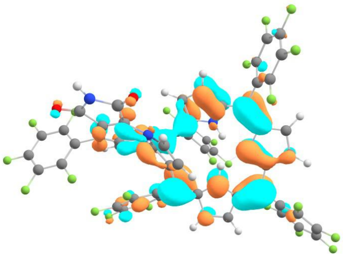	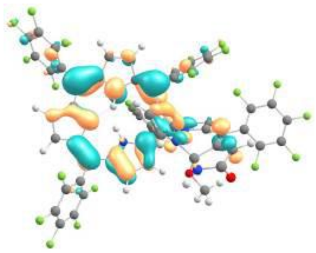
LUMO	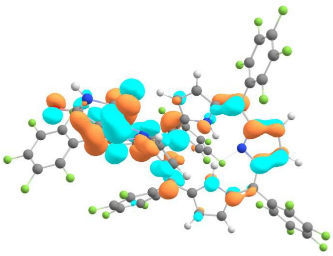	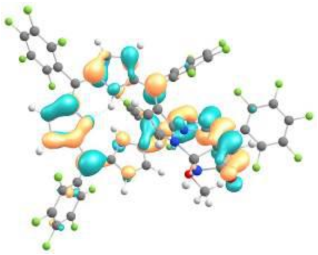

Supplementary Table 9. Excitation energies, oscillator strengths, and molecular orbital assignments for the series of low-lying excited states for **D** calculated using the BMK/6-31G(d)[7] method within the PCM model (CH_2Cl_2 as the solvent)[12].

Excited state	Wavelength	Oscillator strength	MO compositions
S1	1034.7	0.2636	H→L(91%) H-1→L+1(5%)
S2	997.2	0.0050	H-1→L(90%) H→L+1(6%)
S3	749.3	1.8154	H-2→L(93%)
S4	647.6	0.1778	H→L+1(54%) H-1→L+2(36%)
S5	643.4	0.1227	H-1→L+1(47%) H→L+2(40%)
S6	580.5	0.0252	H-3→L(82%)
S7	525.3	0.0215	H-4→L(86%)
S8	524.4	0.0271	H-5→L(79%) H-2→L+1(8%)
S9	517.7	0.0218	H-2→L+1(61%) H-5→L(10%) H-3→L+2(9%) H-1→L+4(6%)
S10	511.5	0.4056	H-2→L+2(30%) H-1→L+3(19%) H→L+4(17%) H→L+2(12%) H-3→L+1(7%) H-1→L+1(6%)
S11	482.1	0.8220	H-6→L(43%) H-2→L+2(15%) H→L+4(10%) H-1→L+3(9%) H-7→L(7%)
S12	478.3	0.1288	H-1→L+2(38%) H→L+3(23%) H→L+1(21%) H-1→L+4(10%)
S13	464.5	0.0932	H→L+2(40%) H-1→L+1(31%) H-2→L+2(14%) H-3→L+1(6%)
S14	454.2	0.1003	H→L+3(24%) H-1→L+2(20%) H-1→L+4(19%) H→L+1(15%) H-2→L+1(12%)
S15	445.7	0.4104	H-2→L+2(20%) H-6→L(19%) H-7→L(18%) H-1→L+3(15%) H→L+4(9%) H-5→L+1(5%)
S16	426.2	0.0025	H-8→L(56%) H-4→L+1(9%) H-5→L+2(6%)
S17	423.7	0.0304	H-2→L+3(35%) H-8→L(20%) H-4→L+1(14%) H-5→L+2(12%)
S18	420.3	0.3708	H-2→L+4(20%) H-4→L+2(20%) H-5→L+1(17%) H-3→L+1(12%) H→L+4(5%)
S19	407	0.2798	H-7→L(34%) H-6→L(14%) H-1→L+5(10%) H-1→L+3(10%) H→L+6(6%) H-2→L+4(6%)
S20	405.6	0.0379	H-2→L+3(35%) H→L+5(12%) H-1→L+6(11%) H-4→L+1(10%) H-3→L+2(9%) H-8→L(7%) H-5→L+2(5%)

Supplementary Table 10. Excitation energies, oscillator strength and molecular orbital assignment for the series of low-lying excited states for **MD1** calculated using the BMK/6-31G(d) method within the PCM model (CH_2Cl_2 as the solvent).

Excited state	Wavelength	Oscillator strength	MO compositions
S1	817.3	0.0965	H-1→L(51%) H→L(45%)
S2	697.8	0.1305	H→L+1(49%) H-1→L+1(40%)
S3	567.8	0.1282	H-2→L+1(64%) H-2→L(8%) H-1→L+2(7%) H→L+3(6%)
S4	562	0.0907	H-1→L+2(34%) H→L+2(31%) H-3→L(16%) H-2→L+1(5%)
S5	543.2	0.069	H→L(47%) H-1→L(42%)
S6	526.8	0.5629	H-3→L(52%) H-2→L(10%) H→L+3(8%) H-1→L+3(7%)
S7	492.7	0.1155	H-1→L+1(42%) H→L+1(32%) H-2→L(9%) H→L+3(5%)
S8	488.3	0.6274	H-2→L(28%) H-3→L(19%) H→L+3(10%) H-1→L+3(9%) H-1→L+1(8%) H→L+1(7%) H-4→L(7%)
S9	477.1	0.6857	H-2→L(31%) H→L+3(21%) H-2→L+1(20%) H-1→L+3(17%)
S10	475.7	0.0781	H-4→L(75%) H-2→L(7%)
S11	450.3	0.1404	H-2→L+3(82%) H-5→L+1(8%)
S12	441.6	0.0526	H→L+2(45%) H-1→L+2(42%)
S13	434	0.3476	H-3→L+2(46%) H-1→L+4(12%) H→L+4(11%) H-2→L+2(7%)
S14	421.1	0.029	H-5→L+1(47%) H→L+5(16%) H-1→L+5(13%)
S15	408.8	0.3856	H-4→L+2(36%) H-3→L+2(12%) H→L+4(12%) H-1→L+4(10%) H-2→L+2(8%)
S16	404.5	0.4135	H-2→L+2(23%) H-5→L+1(16%) H→L+5(13%) H-3→L+2(10%) H-1→L+5(9%)
S17	400.1	0.2916	H-2→L+2(51%) H→L+5(10%) H-5→L+1(8%) H-1→L+5(7%)
S18	394.5	0.5198	H-4→L+2(36%) H-1→L+4(13%) H→L+4(11%) H-1→L+3(11%) H-3→L+1(8%) H→L+3(7%)
S19	393.2	0.1372	H-1→L+3(43%) H→L+3(35%) H-4→L+2(7%)
S20	387.4	0.0625	H-3→L+1(75%) H-3→L+2(12%)

Supplementary Table 11. Excitation energies, oscillator strength and molecular orbital assignment for the series of low-lying excited states for **MD2** calculated using the BMK/6-31G(d) method within the PCM model (CH_2Cl_2 as the solvent).

Excited State	Wavelength	Oscillator strength	MO compositions
S1	838.8	0.3684	H-1→L(78%) H-2→L(11%)
S2	774.5	0.1118	H→L+1(50%) H→L+1(41%)
S3	654.7	0.9295	H-2→L(60%) H-1→L+1(9%) H-1→L(8%) H→L+1(6%)
S4	623.5	0.1405	H→L(46%) H→L+1(27%) H→L+2(9%)
S5	578.1	0.0027	H-1→L+1(38%) H-1→L+2(24%) H-2→L+2(13%) H-2→L(10%) H-3→L(7%)
S6	553.9	0.5256	H-2→L+1(34%) H-1→L+2(33%) H→L+3(12%)
S7	508.9	0.1307	H-3→L(59%) H-2→L(8%) H-1→L+1(8%) H-5→L(7%)
S8	494.5	0.6031	H→L+2(36%) H→L+3(18%) H-2→L+1(9%) H-3→L(8%) H-2→L+2(6%) H→L+1(6%)
S9	484	0.0991	H-5→L(52%) H-1→L+2(11%) H→L+3(7%) H-6→L(6%)
S10	469.4	0.4327	H→L+2(28%) H-2→L+1(15%) H→L+3(13%) H→L+1(6%) H-2→L+2(6%) H→L+4(6%)
S11	469.1	0.106	H-2→L+1(13%) H-1→L+2(12%) H-4→L+1(12%) H-1→L+1(8%) H-5→L(8%) H→L+3(8%) H→L+2(5%) H-4→L(5%)
S12	456.2	0.4103	H-1→L+1(17%) H-2→L+2(16%) H-1→L+3(14%) H-4→L+1(12%) H-2→L+1(8%) H-1→L+2(7%) H-2→L+3(6%) H-4→L(6%)
S13	446.5	0.2541	H-2→L+2(28%) H→L+3(16%) H-4→L+1(7%) H-4→L(6%) H-1→L+1(6%) H-3→L+1(5%)
S14	434.1	0.0451	H-1→L+3(31%) H-4→L(18%) H-2→L+3(11%) H-4→L+1(10%)
S15	422.4	0.2381	H-1→L+4(20%) H-2→L+3(12%) H-2→L+2(11%) H-5→L+2(10%) H-3→L+1(8%)
S16	414	0.183	H-4→L(23%) H→L+5(22%) H→L+4(12%) H→L+6(6%)
S17	403.3	0.3191	H-4→L(24%) H-4→L+1(21%) H-6→L(10%) H-4→L+2(8%) H-3→L+1(6%) H-5→L+1(5%)
S18	400.7	0.0334	H-3→L+1(34%) H-3→L+2(11%) H-1→L+3(8%) H→L+5(8%) H-1→L+4(6%) H→L+4(6%)
S19	396	0.2735	H-6→L(39%) H-5→L+2(10%) H-4→L+1(6%) H→L+6(6%)
S20	389.7	0.1897	H-5→L+2(27%) H-3→L+2(13%) H-1→L+4(11%) H-5→L+1(6%) H-6→L+1(6%) H-6→L(5%)

Supplementary Table 12. Excitation energies, oscillator strength and molecular orbital assignment for the series of low-lying excited states for **$FHPO_2$** calculated using the BMK/6-31G(d) method within the PCM model (CH_2Cl_2 as the solvent).

Excited State	Wavelength	Oscillator strength	MO compositions
1	724.6	0.1173	H→L(82%) H-1→L+1(9%)
2	657	0.0465	H→L+1(60%) H-1→L(31%) H→L(6%)
3	568	0.2758	H-1→L(44%) H-1→L+1(25%) H→L+1(21%)
4	519.9	0.7013	H-1→L+1(59%) H-1→L(16%) H→L+1(9%) H→L(6%) H-2→L(6%)
5	459.2	0.1962	H-2→L(78%) H-2→L+1(5%)
6	423.7	0.0769	H-2→L+1(60%) H→L+2(27%)
7	392.5	0.7781	H→L+2(58%) H-2→L+1(27%)
8	380.2	0.1165	H→L+3(88%)
9	368.1	0.3034	H-1→L+2(59%) H-3→L(15%) H-1→L+3(11%)
10	358.2	0.0767	H-3→L(57%) H-1→L+2(10%) H-1→L+3(8%) H→L+2(6%)
11	348.9	0.0131	H-3→L+1(27%) H-4→L(24%) H-1→L+3(15%) H-3→L(12%) H-1→L+2(8%)
12	340.1	0.1158	H-1→L+3(48%) H-3→L+1(19%) H-1→L+2(12%)
13	333.7	0.0608	H-4→L+1(18%) H-5→L(14%) H-11→L(9%) H-3→L+1(8%) H-19→L(8%) H-10→L(8%)
14	330.5	0.0215	H-3→L+1(18%) H-4→L+1(10%) H-11→L(8%) H-19→L(7%) H-5→L(7%)
15	321.1	0.1012	H-4→L(37%) H-5→L(30%) H-3→L+1(6%)
16	312.6	0.0552	H-12→L(13%) H-4→L+1(12%) H-14→L(12%) H-14→L+1(10%) H-12→L+1(7%) H-10→L(7%) H-5→L(6%) H-18→L+1(5%)
17	307.8	0.3512	H-4→L+1(37%) H-5→L+1(12%) H-5→L(9%) H-4→L(6%) H-3→L+1(5%)
18	300.4	0.0877	H-2→L+2(63%) H-2→L+3(16%)
19	299.6	0.0009	H-6→L(80%) H-6→L+1(14%)
20	299	0.0657	H-5→L+1(37%) H-12→L(10%) H-9→L(8%) H-5→L(7%) H-4→L(5%)

Supplementary Table 13. Excitation energies, oscillator strength and molecular orbital assignment for the series of low-lying excited states for **MFHPO** calculated using the BMK/6-31G(d) method within the PCM model (CH_2Cl_2 as the solvent).

Excited state	Wavelength	Oscillator strength	MO compositions
S1	774.8	0.1059	H→L(98%)
S2	537.9	0.0488	H→L+1(72%) H-1→L(23%)
S3	499	1.1522	H-1→L(70%) H→L+1(23%)
S4	460.8	0.0802	H-2→L(87%)
S5	421.4	0.5264	H→L+2(55%) H-1→L+1(36%)
S6	396.5	0.27	H-1→L+1(53%) H→L+2(22%) H-2→L+1(13%)
S7	385.2	0.3862	H-2→L+1(75%) H→L+2(13%)
S8	356.2	0.1173	H-3→L(78%)
S9	349.7	0.0676	H-4→L(60%) H-3→L(10%) H-1→L+2(9%)
S10	330.6	0.1177	H-1→L+2(75%) H-4→L(14%)
S11	325	0.039	H-5→L(34%) H-7→L(12%) H-9→L(10%) H-8→L(6%) H-20→L(6%)
S12	316.4	0.0546	H→L+3(69%) H-1→L+3(10%)
S13	308	0.113	H-8→L(16%) H-3→L+1(12%) H-5→L(11%) H-9→L(10%) H-4→L(9%) H-7→L(9%) H-13→L(6%)
S14	305.8	0.3997	H-3→L+1(69%) H-5→L(10%)
S15	303.5	0.0689	H-2→L+2(85%)
S16	299.7	0.0438	H-7→L(52%) H-5→L(22%) H-6→L(6%)
S17	297.8	0.0283	H→L+4(53%) H→L+5(15%)
S18	295.9	0.029	H-16→L(21%) H-8→L(16%) H→L+5(16%) H-9→L(8%) H-20→L(8%)
S19	294.8	0.0135	H→L+5(49%) H→L+4(29%)
S20	291.6	0.0393	H-13→L(31%) H-12→L(26%) H-11→L(9%)

Supplementary Methods

Synthesis of D. To a solution of **FHP**[13] (151 mg, 0.12 mmol) in chloroform (50 mL) was added tetrachlorobenzoquinone (145 mg, 0.59 mmol). After heating at reflux for 24 hours under N_2, the reaction was quenched with water, and the organic layer was separated, washed twice with brine and dried over anhydrous Na_2SO_4. Removal of the volatiles followed by silica gel column chromatography (CH_2Cl_2 / hexanes = 1/9 (v/v)) afforded **D** as a light green solid (98 mg, 65%). ^1H NMR ($CDCl_3$, 400 MHz, ppm): δ = 5.59 (t, J = 4.4 Hz, 2H), 6.08 (t, J = 4.8 Hz, 2H), 6.37 (t, J = 4.4 Hz, 2H), 6.57 (d, J = 13.2 Hz, 2H), 6.80-6.89 (m, 6H), 6.92 (d, J = 5.2 Hz, 2H), 6.95 (s, 2H), 7.11 (d, J = 4.4 Hz, 2H), 7.15 (dd, J =5.6, 4.4 Hz, 2H), 9.10 (br s, 2H, NH), 11.30 (br s, 2H, NH).^{13}C NMR ($CDCl_3$, 101 MHz, ppm): δ = 165.97, 156.30, 156.22, 153.21, 153.15, 152.21, 152.08, 150.94, 144.93, 143.31, 143.21, 137.78 (d, $^1J_{CF}$ = 237 Hz*), 134.74, 133.39, 132.98, 132.70, 132.20, 129.57, 129.11, 128.90, 128.23, 125.36, 121.33, 118.51, 115.53, 114.44, 112.82 (t, $^2J_{CF}$ = 26 Hz*), 111.67 (t, $^2J_{CF}$ = 17 Hz*), 99.71, 97.66, 96.31, 92.66. *These signals showed further long-range coupling with the fluorine atoms and are considered less accurate. UV/Vis/NIR [CH_2Cl_2, λ_{max}(nm) ($\varepsilon \times 10^{-5}$/mol^{-1}dm^{3}cm^{-1})]: 442 (0.79), 727 (0.53); HRMS: *m/z*; $[M+H]^+$, calcd for $C_{118}H_{27}F_{50}N_{12}$: 2561.1678; found: 2561.1683.

Synthesis of MD1/2.

Route 1. To a solution of **FHP** (415 mg, 0.32 mmol) in chloroform (100 mL) and methanol (25 mL) was added tetrachlorobenzoquinone (395 mg, 1.6 mmol). The solution was heated with stirring at reflux for 48 hours under N_2 atmosphere and the volatiles were evaporated off in vacuo. The residue was passed through a silica gel column (hexanes/CH_2Cl_2= 4/1, eluent) to afford after removal of solvent a first brown solid **MD2** (8.5 mg, 2%) and a second brown solid **MD1** (76 mg, 18%).

Route 2. To a solution of **D** (92 mg, 0.036 mmol) in THF (40 mL) and methanol (10 mL) was added cesium carbonate (580 mg, 1.78 mmol). After stirring for 0.5 h, the reaction mixture was washed twice with brine and extracted with CH_2Cl_2. The organic layer was evaporated and separated by silica gel column chromatography (hexanes/CH_2Cl_2 = 4/1, eluent) to give **MD1** (52 mg, 56%) and **MD2** (38 mg, 41%). When TFA (280 μL, 3.7 mmol) was added instead of cesium carbonate under otherwise identical reaction and workup conditions, **MD1** (58 mg, 63%) and **MD2** (14 mg, 15%) were again obtained. **MD1**: ^1H NMR (CD_2Cl_2, 400 MHz, ppm): δ = 3.32 (s, 3H, OCH_3), 3.53 (s, 3H, OCH_3), 5.19 (d, J = 2.8 Hz, 1H), 5.29 (d, J = 2.8 Hz, 2H), 5.44 (d, J = 2.8 Hz, 1H), 5.73 (s, 1H), 5.76 (d, J = 3.2 Hz, 1H), 6.03 (dd, J = 4.4, 1.2 Hz, 2H), 6.11 (t, J = 4.2 Hz, 2H), 6.17 (s, 1H), 6.23 (dd, J = 4.0, 2.8 Hz, 1H), 6.39 (s, 1H), 6.41 (s, 1H), 6.45-6.51 (m, 2H), 6.59 (d, J = 2.4 Hz, 1H), 6.65 (s, 1H), 6.70 (d, J = 4.4 Hz, 1H), 6.72 (d, J = 2.8 Hz, 1H), 6.75 (d, J = 3.6 Hz, 1H), 6.77 (d, J = 4.4 Hz, 1H), 6.80 (d, J = 4.0 Hz, 1H), 6.83 (s, 1H), 6.86 (s, 1H), 6.88 (d, J = 3.6 Hz, 2H), 6.92 (d, J = 2.8 Hz, 1H), 6.94 (d, J = 4.4 Hz, 1H), 7.02 (d, J = 3.2 Hz, 1H), 7.04-7.07 (m, 2H), 7.11 (d, J = 3.2 Hz, 1H), 7.15 (d, J = 3.6, 1.2 Hz, 1H), 7.34 (dd, J = 3.2 Hz, 1H), 8.34 (s, 1H, NH), 8.45 (s, 1H, NH), 9.24 (s, 2H, NH), 10.27 (s, 2H, NH), 10.95 (s, 1H, NH), 11.09 (s, 1H, NH), 12.77 (s, 1H, NH), 15.02 (s, 1H, NH). ^1H NMR ($CDCl_3$, 400 MHz, ppm): δ = 3.41 (s, 3H), 5.28 (d, J = 3.6 Hz, 1H), 5.40 (d, J = 3.6 Hz, 1H), 5.80 (s, 1H), 6.06 (d, J = 4.0 Hz, 1H), 6.14 (d, J = 5.6 Hz, 1H), 6.21 (s, 1H), 6.26 (dd, J = 5.2 Hz, 3.6 Hz, 1H), 6.38 (dd, J = 3.2 Hz, 1.2 Hz, 1H), 6.43 (dd, J = 3.2 Hz, 1.2 Hz, 1H), 6.54 (dd, J = 5.2 Hz, 4.0 Hz, 1H), 6.64 (d, J = 3.2 Hz, 1H), 6.69-6.74 (m, 2H), 6.75 (d, J = 3.6 Hz, 1H), 6.80 (d, J = 4.4 Hz, 1H), 6.82 (s, J = 5.2 Hz, 1H), 6.86-6.91 (m, 1H), 6.94 (d, J = 4.4 Hz, 1H), 6.97 (d, J = 4.8 Hz, 1H), 7.05-7.11 (m, 2H), 7.17 (d, J = 4.4 Hz, 1H), 8.53 (s, 1H, NH), 9.32 (s, 1H, NH), 10.35 (s, 1H, NH), 10.96 (s, 1H, NH). ^{13}C NMR ($CDCl_3$, 101 MHz, ppm): δ = 166.22, 164.55, 152.22,

152.06, 151.42, 151.22, 150.69, 150.31, 149.92, 146.86, 144.61, 144.36, 142.68, 140.83, 137.77 (d, ${}^{1}J_{CF}$ = 252 Hz*), 135.40, 134.70, 134.61, 133.88, 133.08, 132.27, 131.64, 130.19, 129.58, 129.43, 129.12, 128.79, 128.52, 128.32, 128.13, 127.97, 126.27, 121.09, 119.73, 118.42, 118.10, 117.57, 115.60, 114.84 (t, ${}^{2}J_{CF}$ = 17 Hz*), 113.83, 112.82, 112.00 (d, ${}^{2}J_{CF}$ = 18 Hz*), 111.62, 111.30, 110.96, 110.65, 109.30, 107.04, 101.68, 100.17, 97.69, 97.52, 94.98, 92.56, 91.66, 91.42, 51.53. *These signals showed further long-range coupling with the fluorine atoms and are considered less accurate. UV/Vis/NIR [CH_2Cl_2, λ_{max}(nm) ($\varepsilon \times 10^{-5}$/mol^{-1}dm^{3}cm^{-1})]: 423 (0.84), 479 (0.79), 693 (0.31); HRMS: *m/z*; $[M]^+$, calcd for $C_{119}H_{30}F_{50}N_{12}O$: 2592.1862; found: 2592.1880. **MD2**: ^{1}H NMR ($CDCl_3$, 400 MHz, ppm): δ = 3.43 (s, 3H), 5.26 (d, *J* = 3.6 Hz, 1H), 5.35 (d, *J* = 3.6 Hz, 1H), 5.78 (s, 1H), 6.08 (d, *J* = 4.8 Hz, 1H), 6.14 (d, *J* = 5.6 Hz, 1H), 6.19 (s, 1H), 6.27 (dd, *J* = 5.6 Hz, 3.6 Hz, 1H), 6.39 (dd, *J* = 4.4 Hz, 2.4 Hz, 1H), 6.51 (m, 2H), 6.55 (d, *J* = 3.6 Hz, 1H), 6.67 (d, *J* = 3.6 Hz, 1H), 6.73 (d, *J* =5.2 Hz, 1H), 6.77 (m, 2H), 6.81 (d, *J* = 5.2 Hz, 1H), 6.87 (s, 1H), 6.94 (d, *J* = 4.8 Hz, 1H), 6.97 (d, *J* = 5.2 Hz, 1H), 7.05 (m, 2H), 7.20 (d, *J* = 3.6 Hz, 1H), 8.56 (s, 1H, NH), 9.38 (s, 1H, NH), 10.27 (s, 1H, NH), 10.95 (s, 1H, NH), 15.58 (s, 1H, outer NH). UV/Vis/NIR [CH_2Cl_2, λ_{max}(nm) ($\varepsilon \times 10^{-5}$/mol^{-1}dm^{3}cm^{-1})]: 422 (0.82), 480 (0.74), 696 (0.33); HRMS: *m/z*; $[M+H]^+$, calcd for $C_{119}H_{30}F_{50}N_{12}O$: 2593.1945; found: 2593.1936.

Synthesis of FHPO$_2$. To a solution of **D** (156 mg, 0.061 mmol) in CH_2Cl_2 (40 mL) and methanol (5 mL) was added silver trifluoroacetate (88 mg, 0.40 mmol). After stirring for 1 h, the reaction mixture was washed twice with brine. The residue was first passed through a flash silica gel column (CH_2Cl_2, eluent) and the obtained eluent was evaporated and separated carefully by silica gel column chromatography (hexanes/CH_2Cl_2 = 1/1, eluent) to give **FHPO$_2$** as a violet solid (12.5 mg, 8%). ^{1}H NMR ($(CD_3)_2CO$, 400 MHz, ppm): δ = 4.90 (d, *J* = 3.6 Hz, 1H), 6.75 (dd, *J* = 3.6 Hz, 2.4 Hz, 1H), 6.81 (dd, *J* = 3.6 Hz, 2.4 Hz, 1H), 6.96 (dd, *J* = 5.6 Hz, 2.8 Hz, 1H), 7.07 (d, *J* = 3.6 Hz, 1H), 7.19 (dd, *J* = 5.6 Hz, 2.8 Hz, 1H), 7.30 (m, 1H), 7.38 (d, *J* = 4.0 Hz, 1H), 7.45 (d, *J* = 5.2 Hz, 1H), 7.63 (dd, *J* = 4.4 Hz, 1.6 Hz, 1H), 7.79 (s, NH, 1H), 9.32 (s, NH, 1H), 9.96 (s, NH, 1H). ^{13}C NMR ($(CD_3)_2CO$, 101 MHz, ppm): δ = 169.35, 166.02, 164.49, 153.04, 152.81 (t, ${}^{2}J_{CF}$ = 25 Hz*), 152.79, 150.98, 147.34, 136.56, 134.36, 133.46, 133.04, 131.46, 131.02, 130.10 (t, ${}^{2}J_{CF}$ = 21 Hz*), 126.34, 121.67, 119.90, 119.69, 116.40, 115.46, 112.32, 99.59 (d, ${}^{2}J_{CF}$ = 43 Hz*), 95.83, 93.13. *These signals showed further long-range coupling with the fluorine atoms and are considered to be less accurate. HRMS: *m/z*; $[M+H]^+$, calcd for $C_{59}H_{14}F_{25}N_6O_2$: 1313.0779; found: 1313.0767.

Synthesis of FHPMO. To a solution of **MD1** (145 mg, 0.056 mmol) in CH_2Cl_2 (40 mL) and methanol (5 mL) was added silver trifluoroacetate (102 mg, 0.46 mmol). After heating at reflux for 30 min, the reaction mixture was washed twice with brine. Following evaporative removal of the volatiles, the residue was separated by silica gel column chromatography (hexanes/CH_2Cl_2 = 1/3, eluent) to give **FHPMO** as a red solid (137 mg, 92%). ^{1}H NMR ($CDCl_3$, 400 MHz, ppm): δ = 3.47 (s, 3H, OCH_3), 5.38 (s, 1H), 5.43 (d, *J* = 3.2 Hz, 1H), 6.15 (d, *J* = 5.6 Hz, 1H), 6.38 (dd, *J* = 3.6, 1.6 Hz, 1H), 6.52 (d, *J* =3.2 Hz, 2H), 6.65 (s, 1H, outer NH), 6.72 (d, *J* = 5.2 Hz, 1H), 6.78 (d, *J* = 4.4 Hz, 1H), 6.82 (d, *J* = 5.2 Hz, 1H), 6.97 (dd, *J* = 4.9, 2.8 Hz, 1H), 7.05 (dd, *J* = 4.4, 0.8 Hz, 1H), 9.32 (s, 1H, inner NH), 10.90 (s, 1H, inner NH). ^{13}C NMR (DMSO-d_6, 101 MHz, ppm): δ = 169.76, 163.94, 153.21, 150.67, 150.50, 149.97, 145.68, 143.27, 137.32 (d, ${}^{1}J_{CF}$ = 246 Hz*), 133.92, 133.33, 132.15, 131.24, 130.86, 130.44, 129.30, 129.01, 128.31, 118.94, 118.72, 118.05, 117.02, 114.71 (t, ${}^{2}J_{CF}$ = 21 Hz*), 112.07, 111.49 (t, ${}^{2}J_{CF}$ = 18 Hz*), 110.84, 109.25, 100.23, 100.13, 99.00, 92.53, 89.96, 50.86. *These signals showed further long-range coupling with the fluorine atoms and are considered less accurate. UV/Vis/NIR [CH_2Cl_2, λ_{max}(nm) ($\varepsilon \times 10^{-5}$/mol^{-1}dm^{3}cm^{-1})]: 325 (0.30), 392 (0.33), 468 (0.41), 524 (0.43), 762 (0.069); HRMS: *m/z*; $[M+H]^+$, calcd for $C_{60}H_{18}F_{25}N_6O_2$:

1329.1092; found: 1329.1089.

Synthesis of FHPMOCl. To a solution of **FHPMO** (110 mg, 0.082 mmol) in chloroform (40 mL) and methanol (10 mL) was added anhydrous $FeCl_3$ (350 mg, 2.2 mmol). After heating at reflux for 3 hours, the reaction mixture was washed thrice with brine. After evaporative removal of the volatiles, the residue was separated by silica gel column chromatography (hexanes/CH_2Cl_2 = 1/3, eluent) to give **FHPMOCl** as a red solid (88 mg, 78%). ^1H NMR ($CDCl_3$, 400 MHz, ppm): δ = 3.54 (s, 3H, OCH_3), 5.46 (d, J = 3.6 Hz, 1H, βH), 6.20 (dd, J = 5.6, 1.6 Hz, 1H), 6.40 (dd, J = 4.0, 2.0 Hz, 1H), 6.51 (d, J =3.6 Hz, 1H), 6.59 (dd, J = 5.6, 3.6 Hz, 1H), 6.70 (d, J = 5.6 Hz, 1H), 6.77 (d, J = 3.6 Hz, 1H), 6.81 (d, J = 5.2 Hz, 1H), 6.94 (dd, J = 4.0, 2.4 Hz, 1H), 7.05 (dd, J = 4.8, 1.2 Hz, 1H), 7.37 (s, 1H, outer NH), 9.31 (s, 1H, inner NH), 10.91 (s, 1H, inner NH). ^{13}C NMR (DMSO-d_6, 101 MHz, ppm): δ = 164.62, 163.44, 153.87, 150.55, 149.20, 146.45, 143.80, 141.69, 138.7, 137.31 (d, $^1J_{CF}$ = 262 Hz*), 133.57, 131.94, 131.73, 131.02, 129.10, 128.98, 128.75, 128.43, 119.36, 119.28, 118.37, 116.33, 114.69 (t, $^2J_{CF}$ = 17 Hz*), 112.47, 111.88, 111.63, 111.47, 110.05 (t, $^2J_{CF}$ = 20 Hz*), 101.17, 109.53 (t, $^2J_{CF}$ = 18 Hz*), 99.77, 90.12, 88.77, 51.31. *These signals showed further long-range coupling with the fluorine atoms and are considered less accurate. UV/Vis/NIR [CH_2Cl_2, λ_{max}(nm) (ε × 10^{-5}/$mol^{-1}dm^3cm^{-1}$)]: 328 (0.35), 389 (0.39), 472 (0.46), 531 (0.56), 740 (0.059); HRMS: *m/z*; $[M+H]^+$, calcd for $C_{60}H_{18}F_{25}N_6O_2$: 1363.0702; found: 1363.0712.

Cartesian coordinates for the optimized structures (1—H, 6—C, 7—N, 8—O, 9—F, 17—Cl).

D

9	15.658083171	-0.757960042	-1.687617136
6	14.621334081	-1.567701099	-1.932593155
6	14.823886092	-2.771819185	-2.601601206
6	13.742303994	-3.613743247	-2.848433224
6	12.468900911	-3.237412221	-2.429870192
6	12.226716910	-2.037581138	-1.748571142
6	13.339941003	-1.221378075	-1.510420123
9	13.192493965	-0.057684992	-0.859200080
6	10.859850806	-1.665242109	-1.293648108
6	10.237747757	-0.539794032	-1.835933146
6	10.759369783	0.506190046	-2.695536210
1	11.747821872	0.510334047	-3.132021243
6	9.807438704	1.471928114	-2.820913218
1	9.879697751	2.383000180	-3.398198262
6	8.618280639	1.046344085	-2.103968170
7	8.935913673	-0.186808005	-1.569602132
1	8.413786653	-0.715150042	-0.869658077
6	7.386487557	1.659513125	-1.976201160
6	7.251708545	3.080912230	-2.373764190
6	8.093028615	4.081030301	-1.858274153
9	9.029415695	3.774889282	-0.944047084
6	7.985299610	5.416356372	-2.233257179
9	8.800575639	6.336246457	-1.702256142
6	7.008803541	5.799398413	-3.148546242
9	6.898585498	7.078014527	-3.517334270
6	6.149938441	4.839903354	-3.678831281
6	6.273486459	3.509729257	-3.287771252
9	5.441076411	2.622858192	-3.847894295
9	5.228822395	5.197797379	-4.578091344
6	6.185826448	0.912690073	-1.598708129
6	5.817080448	-0.389186022	-2.110774167
1	6.437778479	-1.011902065	-2.739231212
6	4.527396348	-0.647048042	-1.758736145
7	4.055221316	0.430597035	-1.010000091
6	5.083126389	1.352984102	-0.874158079
7	4.937763378	2.448019183	-0.006785017
6	6.003524463	2.911200213	0.808591044
6	5.857170440	4.351278317	0.859218045
1	6.513786482	5.010606367	1.407809082
6	4.696327358	4.701444344	0.241176001
1	4.258447324	5.689514440	0.205250998
6	4.046491309	3.508034255	-0.251318034
6	2.744019218	3.334245244	-0.664704067

6	1.928195157	4.557725333	-0.918379083
6	2.130500170	5.329706402	-2.067856167
9	3.068240240	4.972187363	-2.956815228
6	1.381602119	6.473766448	-2.333444185
9	1.600832130	7.187496538	-3.441957262
6	0.394727046	6.871278470	-1.434492120
9	-0.329968007	7.964886598	-1.677234138
6	0.166887031	6.125291428	-0.280088037
6	0.933897089	4.989490358	-0.033626019
9	0.695402068	4.301338310	1.091841062
9	-0.773973036	6.510305449	0.588439026
6	2.095717175	2.048373152	-0.748174068
6	2.707589217	0.686074054	-0.793134073
7	1.834283156	-0.283360018	-0.819119073
6	0.592477066	0.356745027	-0.802441073
6	0.743056077	1.784154132	-0.786573074
1	-0.076292984	2.486633178	-0.776774071
6	-0.592564021	-0.356786026	-0.802450074
6	-0.743138031	-1.784197127	-0.786573074
1	0.076211031	-2.486678179	-0.776736071
6	-2.095798123	-2.048415148	-0.748198071
6	-2.707675171	-0.686122050	-0.793173073
7	-1.834371110	0.283315020	-0.819157073
7	-4.055308270	-0.430662034	-1.010035091
6	-4.527507301	0.646973041	-1.758770145
1	-3.849963256	1.443698100	-2.019295164
6	-5.817190411	0.389089024	-2.110793170
1	-6.437902465	1.011788070	-2.739252215
6	-6.185922441	-0.912779070	-1.598694133
6	-5.083206342	-1.353053102	-0.874155079
7	-4.937816327	-2.448072180	-0.006766017
6	-4.046546264	-3.508087257	-0.251300034
6	-2.744094171	-3.334290240	-0.664733066
6	-1.928241111	-4.557750331	-0.918424084
6	-2.130487126	-5.329689363	-2.067938163
6	-1.381546068	-6.473716440	-2.333546184
9	-1.600724083	-7.187415521	-3.442088265
6	-0.394684999	-6.871229468	-1.434578122
9	0.330050054	-7.964807592	-1.677339138
6	-0.166893983	-6.125273436	-0.280145036
9	0.773964087	-6.510279466	0.588387024
6	-0.933944038	-4.989501360	-0.033667019
9	-0.695477020	-4.301350312	1.091808062
9	-3.068213193	-4.972163360	-2.956909231
6	-4.696365311	-4.701490340	0.241234001
1	-4.258479276	-5.689559420	0.205318998
6	-5.857184410	-4.351312317	0.859316044

6	-6.003543407	-2.911234212	0.808665044
6	-6.845469490	-2.091591153	1.519184092
6	-6.536271435	-0.679970053	1.738982106
6	-5.326419365	-0.108319011	2.179952139
1	-4.405886293	-0.652537049	2.336399153
6	-5.556788372	1.246248088	2.431422157
1	-4.835062324	1.970817141	2.781816185
6	-6.898320486	1.524725107	2.106417134
6	-7.605407549	2.775891194	2.064008133
6	-7.090330493	3.886717272	2.900136191
6	-6.760713446	5.136816363	2.353772154
9	-6.892191502	5.358363361	1.039874057
6	-6.267271431	6.179001438	3.134296208
9	-5.955304403	7.353328516	2.575975168
6	-6.085102427	5.990955445	4.501960306
9	-5.613786371	6.984182511	5.257821361
6	-6.398786424	4.762281338	5.078548349
6	-6.892255480	3.733890264	4.281585293
9	-7.204935521	2.578755179	4.882488337
9	-6.237379425	4.582694323	6.393892434
6	-8.720347606	2.960479209	1.251139072
7	-9.084316617	2.014786140	0.316591006
6	-10.217297702	2.436732165	-0.265708035
6	-10.859942777	1.665136109	-1.293553110
6	-10.237854703	0.539676030	-1.835831147
7	-8.936021625	0.186681007	-1.569499127
1	-8.413894605	0.715005047	-0.869541082
6	-8.618382611	-1.046456080	-2.103901166
6	-7.386581486	-1.659616124	-1.976170160
6	-7.251792473	-3.080998227	-2.373802190
6	-6.273593405	-3.509760256	-3.287860255
9	-5.441212357	-2.622856192	-3.847973296
6	-6.150055389	-4.839912355	-3.679002280
9	-5.228975349	-5.197755378	-4.578319345
6	-7.008898475	-5.799442441	-3.148743244
9	-6.898685460	-7.078037494	-3.517611268
6	-7.985364540	-5.416458398	-2.233399178
6	-8.093091534	-4.081153298	-1.858342152
9	-9.029454642	-3.775073277	-0.944071083
9	-8.800619614	-6.336383461	-1.702427140
6	-9.807543693	-1.472028112	-2.820845219
1	-9.879801682	-2.383087182	-3.398151260
6	-10.759482763	-0.506302045	-2.695435212
1	-11.747936809	-0.510445046	-3.131915244
6	-12.226815866	2.037483139	-1.748449140
6	-12.468976901	3.237242223	-2.429884194
9	-11.451959824	4.070863282	-2.695604210

6	-13.742381995	3.613588251	-2.848425223
9	-13.930241982	4.768508331	-3.497395268
6	-14.823995050	2.771749186	-2.601437203
6	-14.621470026	1.567708100	-1.932283157
6	-13.340073933	1.221371079	-1.510135124
9	-13.192660921	0.057760994	-0.858756079
9	-15.658250127	0.758054040	-1.687148136
9	-16.049125155	3.117289213	-3.005457233
6	-10.634203753	3.711971261	0.314584006
6	-9.690075657	4.045868283	1.238058075
1	-9.675742679	4.921633346	1.873257118
1	-11.512534797	4.282146300	0.044248987
7	-7.466381521	0.324563018	1.729227110
1	-8.390730559	0.284501014	1.313628079
6	-8.018070552	-2.677573199	2.239106143
6	-7.854313530	-3.515596261	3.348904226
9	-6.622099434	-3.844954281	3.766918253
6	-8.936987638	-4.044133296	4.047842272
6	-10.233526721	-3.721979278	3.655542249
6	-10.435269727	-2.878421217	2.565622169
6	-9.334609624	-2.377177177	1.877963118
9	-9.568700653	-1.553113117	0.839350043
9	-11.676102797	-2.563759195	2.184282142
9	-11.277856805	-4.217140315	4.321823296
9	-8.737325623	-4.848373354	5.097153348
1	-6.513777434	-5.010627365	1.407950087
6	6.845490499	2.091592159	1.519107091
6	8.018116590	2.677632202	2.238936145
6	9.334641692	2.377166180	1.877801121
6	10.435326772	2.878463218	2.565382166
9	11.676144866	2.563723195	2.184058141
6	10.233623772	3.722160277	3.655203247
9	11.277977828	4.217378312	4.321405293
6	8.937098660	4.044395302	4.047484274
9	8.737473634	4.848771359	5.096697351
6	7.854399574	3.515797259	3.348631224
9	6.622200507	3.845247281	3.766617252
9	9.568691709	1.552961121	0.839291045
6	6.536325517	0.679981054	1.739004110
7	7.466450578	-0.324541016	1.729269106
1	8.390770617	-0.284495013	1.313602080
6	6.898421506	-1.524691102	2.106546136
6	7.605513597	-2.775857194	2.064158134
6	8.720400629	-2.960484204	1.251223074
7	9.084313654	-2.014831136	0.316613006
6	10.217254785	-2.436803165	-0.265747036
6	10.634187772	-3.712024258	0.314563006

1	11.512497860	-4.282214299	0.044188987
6	9.690120743	-4.045879284	1.238117073
1	9.675826713	-4.921619344	1.873351116
6	7.090482525	-3.886647273	2.900361192
6	6.892473501	-3.733757260	4.281814294
9	7.205173545	-2.578593177	4.882649334
6	6.399050483	-4.762115335	5.078848351
9	6.237706457	-4.582470322	6.394191419
6	6.085348442	-5.990819446	4.502332307
6	6.267453468	-6.178926453	3.134669209
6	6.760849497	-5.136774362	2.354073155
9	6.892266540	-5.358381353	1.040178059
9	5.955469453	-7.353282531	2.576417168
9	5.614077419	-6.984015503	5.258262362
6	5.556902410	-1.246212083	2.431604157
6	5.326503398	0.108338013	2.180073140
1	4.405972342	0.652553051	2.336542153
1	4.835204371	-1.970769139	2.782078186
1	3.849841302	-1.443770100	-2.019243162
9	11.451911828	-4.071119282	-2.695427210
9	13.930189011	-4.768731332	-3.497273266
9	16.049015192	-3.117350211	-3.005633232

MD1

9	11.430249798	-4.741117339	5.044662362
6	10.102937736	-4.859073350	4.920550353
6	9.400162673	-5.714510392	5.764791438
9	10.046323708	-6.412111477	6.702822460
6	8.019893567	-5.838478418	5.627035382
9	7.338851550	-6.654067455	6.440393461
6	7.357375505	-5.098871365	4.651000337
6	8.030513573	-4.233227303	3.779134273
6	9.418812678	-4.142243297	3.941663286
9	10.137343729	-3.339370241	3.142024228
6	7.303229507	-3.468023248	2.730455199
6	7.268102510	-2.073135150	2.796801199
6	7.999759590	-1.154520086	3.648216261
1	8.642949612	-1.449309104	4.464848322
6	7.758517578	0.113376008	3.214641232
1	8.150196586	1.028619076	3.636001262
6	6.807999508	0.055703004	2.118741153
7	6.524176454	-1.287094094	1.951086143
1	6.031468408	-1.723615122	1.170914087
6	6.238427461	1.075825078	1.382255097
6	6.859183489	2.420970176	1.448578104

6	8.233001607	2.611304189	1.214990085
9	9.017016648	1.572638112	0.882083064
6	8.841166656	3.860625278	1.282158093
9	10.151101755	3.989747289	1.037728074
6	8.076219564	4.983544359	1.581347115
9	8.646427630	6.189524445	1.647504119
6	6.709868508	4.841460347	1.808426132
6	6.119593460	3.582033258	1.738046124
9	4.806681347	3.502057251	1.984437142
9	5.977675424	5.920698433	2.109274151
6	4.992312360	0.895579063	0.631636045
6	4.621138331	1.497621109	-0.571118039
7	5.394277404	2.208035157	-1.501947106
6	6.664440494	1.770176126	-1.946106140
6	7.036003516	0.455903033	-2.109651153
6	8.474264627	0.130011009	-2.368850170
6	9.245896657	-0.576869042	-1.441944105
6	10.570187752	-0.929449069	-1.682197122
9	11.270033819	-1.606156114	-0.767883058
6	11.166683809	-0.563802041	-2.886712207
9	12.437770895	-0.887655062	-3.130285224
6	10.429954776	0.139317010	-3.836208278
9	10.995951808	0.488029035	-4.996287360
6	9.101653681	0.466291033	-3.573900259
9	8.426223588	1.138974080	-4.519007323
9	8.697224624	-0.962972072	-0.274517020
6	6.072274430	-0.637850047	-2.185293156
7	6.273188426	-1.884252136	-1.646267120
1	6.910840502	-2.114858151	-0.892513063
6	5.270672378	-2.749694200	-2.031884148
6	5.144742369	-4.088852292	-1.519205111
6	5.688179432	-4.454825319	-0.291539021
7	6.201148458	-3.511760255	0.572704041
6	6.693407497	-4.166489299	1.636364118
6	6.515403489	-5.608473379	1.466071106
1	6.824331505	-6.378008438	2.160431155
6	5.870190423	-5.782352397	0.280572020
1	5.582467386	-6.719225473	-0.177568013
6	4.393429317	-5.075692364	-2.331035169
6	4.714046340	-5.313359376	-3.677428263
9	5.730884406	-4.659216338	-4.253752305
6	4.026745288	-6.243238475	-4.451252320
9	4.371618314	-6.450393482	-5.727027392
6	2.985809215	-6.975840508	-3.885955278
6	2.638680190	-6.769010469	-2.553251185
6	3.329926242	-5.823693417	-1.799681129
9	2.935567211	-5.634751421	-0.533558038

9	1.642183120	-7.474624515	-2.010293145
9	2.328047170	-7.877618549	-4.617297334
6	4.437677320	-2.031473146	-2.910340210
6	4.915593354	-0.721698049	-2.984342214
1	4.497525325	0.094933007	-3.551904255
1	3.563089258	-2.429251176	-3.406021243
6	7.383074522	2.977528215	-2.299096163
1	8.390320605	2.994898215	-2.688494195
6	6.546570495	4.043019293	-2.181415155
1	6.768431496	5.067386366	-2.444384174
6	5.255973379	3.579626258	-1.716180123
6	4.076448293	4.280227307	-1.521441107
6	4.111200294	5.771644390	-1.543308113
6	4.331794313	6.487038478	-2.724616194
9	4.537126325	5.825946442	-3.877491279
6	4.351933312	7.878922547	-2.758566200
6	4.125330295	8.595866596	-1.585852112
6	3.883361278	7.916544563	-0.393772029
6	3.880474277	6.523793489	-0.386088028
9	3.626530260	5.898150402	0.771185055
9	3.658091264	8.603127595	0.730556053
9	4.135260296	9.930380708	-1.605684114
9	4.573483332	8.528258593	-3.906752283
6	2.801225200	3.639806264	-1.355335099
6	2.515238182	2.147592156	-1.711797123
7	3.277672238	1.293530091	-0.792551057
6	2.777489198	0.530253040	0.243416017
6	3.803152274	0.239057017	1.104887080
1	3.718968270	-0.328845023	2.020717148
1	1.726746126	0.296441021	0.281005020
8	2.922797212	1.792512126	-3.012108218
6	2.347772167	2.558362185	-4.070163292
1	2.660397190	2.066002147	-4.993234362
1	2.720642194	3.589801257	-4.069820295
1	1.253908093	2.556918182	-4.011384288
7	1.093046078	1.947929140	-1.503213110
6	0.613637044	3.094166221	-1.086963080
6	1.601777114	4.163053298	-0.978864069
1	1.393085101	5.173201374	-0.653165045
6	-0.790954058	3.218804232	-0.784912055
7	-1.627682118	2.148966154	-1.048665077
1	-1.288074094	1.279036090	-1.437771104
6	-2.886895208	2.444615176	-0.639377046
6	-2.897496210	3.736023271	-0.072059005
6	-1.556629113	4.209902302	-0.193790014
1	-1.182310083	5.157221369	0.163776012
6	-4.021686290	4.403256319	0.571724042

6	-4.006319290	5.887520449	0.710594053
6	-3.148908226	6.555406485	1.590887117
9	-2.293219163	5.854480420	2.351839170
6	-3.138249225	7.943060576	1.706704125
9	-2.304385168	8.547003638	2.560004185
6	-4.014109287	8.702687638	0.934280065
9	-4.017087290	10.032848738	1.040653075
6	-4.884315354	8.070918584	0.048927004
6	-4.869545350	6.681795496	-0.053728004
9	-5.709889405	6.106829440	-0.924302068
9	-5.720249425	8.799264656	-0.697228052
6	-5.058468364	3.703735268	1.136312081
7	-5.176138371	2.296721163	1.009168071
6	-5.165105369	1.702013122	-0.269822019
7	-3.963140283	1.622375116	-0.945107069
6	-4.167376301	0.940692069	-2.144668155
1	-3.367686240	0.834760060	-2.861841205
6	-5.483510394	0.592441040	-2.211401158
6	-6.160657455	1.112541079	-1.046585077
6	-7.615148538	1.128774083	-0.874544061
6	-8.293371576	2.386064174	-0.487304035
6	-7.993593564	3.617850260	-1.096101076
9	-7.075658524	3.683413263	-2.069061152
6	-8.648967627	4.798009344	-0.754782052
9	-8.363651606	5.939852427	-1.388348102
6	-9.628392693	4.785956342	0.234742017
6	-9.944759724	3.589376259	0.871609064
6	-9.281578655	2.420972177	0.512357037
9	-9.604343704	1.304858093	1.188182088
9	-10.869295773	3.572249258	1.840090130
9	-10.261513742	5.913795422	0.568300043
6	-8.382513600	0.022151002	-1.192440086
7	-7.873975587	-1.253618088	-1.344689095
1	-6.962373476	-1.587962116	-1.035003075
6	-8.862050637	-2.168303159	-1.620668117
6	-8.650480598	-3.549665253	-1.658001117
6	-7.405680551	-4.111089294	-1.208748085
7	-6.506901463	-3.376417242	-0.533393040
6	-5.404970383	-4.172964302	-0.312958022
6	-4.296187309	-3.698463265	0.386942028
6	-3.034863221	-4.482419325	0.403471029
6	-2.439908176	-4.897480352	1.603556118
6	-1.248781090	-5.617457380	1.629419116
9	-0.720399052	-6.010646447	2.793145203
6	-0.609613043	-5.939831419	0.433582031
9	0.529055038	-6.632095487	0.451354032
6	-1.168882083	-5.535783422	-0.776374056

9	-0.552751042	-5.820466441	-1.930274141
6	-2.363957170	-4.821275346	-0.780098056
9	-2.850100206	-4.435348321	-1.967663144
9	-3.028181220	-4.621769332	2.774272202
6	-4.296876309	-2.433056177	1.056533075
6	-3.219096232	-1.602210113	1.428711102
1	-2.173002157	-1.861404135	1.341705097
6	-3.747068270	-0.395573028	1.888372135
1	-3.199659229	0.459424033	2.259084161
6	-5.153635373	-0.489498035	1.842378134
7	-5.449597382	-1.736187124	1.364494099
1	-6.360047474	-2.072837149	1.065241078
6	-6.137363443	0.472307034	2.341922171
6	-6.027488413	1.805463131	2.032024147
6	-6.585207472	2.978389213	2.674957190
6	-6.022940452	4.097476294	2.137279153
1	-6.167550465	5.114014369	2.478524177
1	-7.258113548	2.946949210	3.519708254
6	-7.167045530	0.008276001	3.319318239
6	-7.111838532	0.370821027	4.671122334
9	-6.137731433	1.183711085	5.110424368
6	-8.048209577	-0.074565005	5.601033411
9	-7.962475560	0.302167022	6.881538476
6	-9.071185666	-0.926907065	5.194452375
6	-9.151242669	-1.319959097	3.860745280
6	-8.210400605	-0.846866059	2.951831215
9	-8.321296577	-1.252754089	1.672623121
9	-10.132590712	-2.134504153	3.461435250
9	-9.970285700	-1.365162101	6.077965414
6	-5.636927398	-5.486748377	-0.891490063
1	-4.953739355	-6.325475478	-0.864163064
6	-6.889440521	-5.457761370	-1.429174101
1	-7.398021557	-6.258435441	-1.949827140
6	-9.729201700	-4.441937318	-2.141026156
6	-10.191758708	-5.523931386	-1.375219098
9	-9.678814712	-5.752533418	-0.158114011
6	-11.189712809	-6.383921464	-1.824603133
9	-11.604609839	-7.399551514	-1.058632078
6	-11.773279848	-6.172494462	-3.071313220
6	-11.346879828	-5.105638367	-3.857877276
6	-10.337452741	-4.266936306	-3.393845245
9	-9.946310742	-3.266823236	-4.199248303
9	-11.896271859	-4.902851353	-5.061451363
9	-12.737277915	-6.986682503	-3.508898253
6	-10.081148704	-1.398252099	-1.758068127
6	-9.801848687	-0.096112007	-1.467590104
1	-10.494103759	0.733969051	-1.482162105

1	-11.045652770	-1.812709129	-2.015095142
1	-5.961112441	0.072784005	-3.029808218
9	6.026675438	-5.239239376	4.556547326

MD2

9	-9.565978671	-4.038817033	7.616337494
6	-9.763415549	-4.287752991	6.316526720
6	-10.759024072	-5.178642168	5.923877307
9	-11.525061163	-5.776011901	6.839861323
6	-10.952381417	-5.440968618	4.569756130
9	-11.915938414	-6.285760831	4.185718045
6	-10.153562906	-4.805624120	3.623482495
6	-9.134629361	-3.907506747	3.978830235
6	-8.966827097	-3.675567104	5.353013273
9	-8.006450613	-2.842108370	5.782726155
6	-8.276833570	-3.260305202	2.960157681
6	-8.148988527	-1.868527464	2.945417428
6	-8.928583722	-0.857128330	3.632697715
1	-9.703961691	-1.066139981	4.355833205
6	-8.546527591	0.364999133	3.173287797
1	-8.933671867	1.325003088	3.484262401
6	-7.463477343	0.186277330	2.220894715
7	-7.250622092	-1.180973159	2.165022138
1	-6.702469764	-1.681620721	1.469624314
6	-6.748001656	1.131481121	1.518387024
6	-7.240150944	2.532236525	1.540247564
6	-8.524453914	2.880503037	1.095360884
9	-9.335930345	1.946719277	0.569603623
6	-9.008093344	4.183896638	1.158013671
9	-10.235249775	4.473299823	0.707364977
6	-8.201730142	5.193570062	1.676023492
9	-8.653451981	6.447971756	1.737566425
6	-6.920594668	4.887478130	2.127087644
6	-6.453244742	3.580304161	2.043863985
9	-5.219557708	3.332090413	2.502151719
9	-6.143075435	5.852892942	2.626686827
6	-5.445295488	0.845844995	0.902622962
6	-4.352281262	0.181810513	1.556019866
1	-4.396983309	-0.298019042	2.523478096
6	-3.225404814	0.351357802	0.796365451
7	-3.570668087	1.040351608	-0.349648921
6	-4.917729007	1.329982820	-0.297296132
7	-5.537951250	2.001254384	-1.365404533
6	-6.804910012	1.629025879	-1.881529508
6	-7.268616129	0.337035319	-1.984456658
6	-8.704278027	0.096582388	-2.335162143

6	-9.596688996	-0.475651388	-1.424536492
9	-9.170005919	-0.802789769	-0.191306337
6	-10.922035959	-0.750831702	-1.746252652
9	-11.740710576	-1.297044691	-0.843402049
6	-11.393678094	-0.445233242	-3.020828773
9	-12.663204478	-0.696281862	-3.343306156
6	-10.533304151	0.121901357	-3.957389169
6	-9.208017620	0.373860768	-3.611059440
9	-8.410144537	0.914484031	-4.546221623
9	-10.978476099	0.412048453	-5.184105388
6	-6.385242079	-0.821261045	-1.932293995
7	-6.694143583	-2.009050261	-1.323262142
1	-7.381110692	-2.162860925	-0.589598914
6	-5.740495271	-2.965977334	-1.606599760
6	-5.737188865	-4.264083210	-0.990472784
6	-6.428781143	-4.525906492	0.184232178
7	-7.046884141	-3.512946544	0.890048854
6	-7.606689058	-4.063061437	1.974596435
6	-7.371690014	-5.506869019	1.987568130
1	-7.701272506	-6.198801504	2.751319001
6	-6.621403196	-5.787997872	0.887386568
1	-6.246852915	-6.754734219	0.578385981
6	-4.948147560	-5.350629595	-1.641002129
6	-5.582179461	-6.419866411	-2.283543401
9	-6.920326904	-6.477810949	-2.326003472
6	-4.865303121	-7.447844071	-2.891313843
9	-5.504691666	-8.454568332	-3.495959224
6	-3.473021634	-7.420664998	-2.872026528
6	-2.810600014	-6.368791705	-2.243739074
6	-3.549638260	-5.357188958	-1.635794876
9	-2.876871749	-4.363085278	-1.035634320
9	-1.474158448	-6.340052564	-2.218806687
9	-2.776177193	-8.398601859	-3.453840789
6	-4.827253361	-2.368714500	-2.499275227
6	-5.207834744	-1.038070044	-2.675260721
1	-4.721933520	-0.294786113	-3.286793619
1	-3.967984843	-2.854639325	-2.937674697
6	-7.375430557	2.844453769	-2.418691312
1	-8.340982235	2.903838690	-2.898436743
6	-6.444242220	3.834031343	-2.353470131
1	-6.533512464	4.831115260	-2.761190745
6	-5.250545867	3.320000936	-1.722319759
6	-4.022283710	3.946778012	-1.540103162
6	-3.934832931	5.414070485	-1.780042461
6	-4.721453054	6.333703707	-1.072592187
9	-5.593850200	5.897576033	-0.150939173
6	-4.647101073	7.707896324	-1.284248804

9	-5.419244092	8.542707301	-0.583491446
6	-3.753663379	8.210482786	-2.226401000
9	-3.669067517	9.523868703	-2.438631520
6	-2.952491495	7.330826312	-2.951165832
6	-3.055993078	5.961159485	-2.727913508
9	-2.277775427	5.158434476	-3.472871596
9	-2.103416323	7.803310051	-3.868510352
6	-2.818962570	3.250447190	-1.183574890
6	-2.657812408	1.711908795	-1.286957731
7	-1.288128662	1.530363385	-0.892996937
6	-0.654944985	2.717403756	-0.632939520
6	-1.612989110	3.771745837	-0.792163620
1	-1.390658146	4.815221389	-0.618068740
6	0.690881254	2.776140796	-0.293768025
6	1.494538741	3.916856335	-0.002330877
1	1.173876130	4.950077528	0.005688647
6	2.779189645	3.447592524	0.238084589
6	2.648813008	1.993943478	0.089626146
7	1.433803528	1.601048553	-0.219965839
7	3.636698596	1.068456529	0.440343336
6	3.375761951	-0.125342856	1.108589733
1	2.367274836	-0.504779259	1.137863591
6	4.526831806	-0.543433543	1.706387968
1	4.635420946	-1.417265326	2.333021791
6	5.558007120	0.436166961	1.449785544
6	4.972356271	1.380188533	0.608814398
7	5.533820069	2.470592551	-0.085908610
6	5.236717471	3.803055598	0.280285171
6	3.958596646	4.242555428	0.511469103
6	3.757732154	5.648241578	0.969537711
6	3.467810042	5.918896955	2.312195041
9	3.395946041	4.913050038	3.191436085
6	3.256248275	7.215408997	2.773604449
9	2.983950564	7.444846970	4.062408061
6	3.334076881	8.282624568	1.880691583
9	3.133974198	9.530502945	2.311956366
6	3.624304064	8.047258963	0.539194781
6	3.828855307	6.740446433	0.100408621
9	4.107269978	6.547518198	-1.197406827
9	3.700042420	9.072283285	-0.317955632
6	6.464648036	4.547013494	0.131659778
1	6.563349171	5.606747203	0.324167315
6	7.412739987	3.730862259	-0.405149153
6	6.845805677	2.414744480	-0.618894658
6	7.366269758	1.333575653	-1.287192892
6	6.514067529	0.252419348	-1.778880690
6	5.318569814	0.345471750	-2.519652822

1	4.809193341	1.269403143	-2.753875653
6	4.963047863	-0.941677746	-2.927185128
1	4.097588331	-1.219945798	-3.512436470
6	5.912918946	-1.841081210	-2.403175623
7	6.854143711	-1.072702663	-1.746115643
1	7.570390039	-1.494609804	-1.165419364
6	5.942911971	-3.275138453	-2.419723393
6	6.629895591	-4.010522830	-1.454520913
7	7.150779468	-3.405151413	-0.332868842
6	7.790169045	-4.350709669	0.376897180
6	8.440707381	-4.042741353	1.618608459
6	8.295485322	-2.786506632	2.212735225
7	7.414727365	-1.832441333	1.763514436
6	7.581144667	-0.629179737	2.420223708
6	6.840522583	0.508431330	2.148604795
6	7.285014962	1.810444922	2.694417122
6	6.405813051	2.691933339	3.351404869
9	5.125194969	2.354656901	3.550396931
6	6.812861906	3.932864032	3.833311053
9	5.937320473	4.737961585	4.447841021
6	8.138226914	4.333389391	3.690430556
9	8.538410435	5.519501326	4.157683409
6	9.040757551	3.490849949	3.047830286
6	8.609852750	2.263676420	2.554730474
9	9.515005725	1.524536995	1.891502716
9	10.311365572	3.878793849	2.879175533
6	8.609847882	-0.872964604	3.412881269
1	8.948127560	-0.143690575	4.135602880
6	9.008203678	-2.173071003	3.315819393
1	9.744755066	-2.672135437	3.929145736
1	6.897913178	-2.002734028	0.899733226
6	9.321606681	-5.056045007	2.259713215
6	8.807247559	-6.254639917	2.771035744
9	7.490397595	-6.501151741	2.699530276
6	9.616188966	-7.223271195	3.359096154
9	9.083693141	-8.354909228	3.836171706
6	10.987133386	-7.002898029	3.464418005
6	11.534351134	-5.820529625	2.973797188
6	10.705767832	-4.873998641	2.375626052
9	11.277908982	-3.755309380	1.905797688
9	12.852362431	-5.606520397	3.066942840
9	11.772851532	-7.920959277	4.034400784
6	7.704977488	-5.635952177	-0.311739624
6	6.963545762	-5.426344699	-1.435886471
1	6.703802549	-6.153206428	-2.194264960
1	8.133223120	-6.571363454	0.022109740
6	5.186837853	-3.967028286	-3.491808453

6	4.203061919	-4.926683634	-3.207253738
9	3.904764550	-5.236885257	-1.939394374
6	3.485127664	-5.572128691	-4.210897537
9	2.547285262	-6.474007805	-3.899728259
6	3.734707393	-5.264053731	-5.545539546
9	3.050505928	-5.876855252	-6.514568818
6	4.701533806	-4.314072918	-5.865867521
6	5.410140762	-3.682273267	-4.848291930
9	6.347778658	-2.795502968	-5.205263075
9	4.952268020	-4.024755841	-7.147968743
6	8.812079794	1.325640424	-1.667468162
6	9.720423020	0.414887448	-1.119429183
9	9.298572230	-0.479269178	-0.205339640
6	11.059564046	0.369479105	-1.494624976
9	11.890720896	-0.520398718	-0.944196741
6	11.533370886	1.269913043	-2.445549295
6	10.661280430	2.194088114	-3.014473640
6	9.322437912	2.205103917	-2.630678643
9	8.516526469	3.108246579	-3.210836627
9	11.109982678	3.057522792	-3.932686456
9	12.816992276	1.245688113	-2.811348832
1	8.403645394	4.023927387	-0.720361311
1	-0.776668464	0.664461513	-0.993271173
8	-2.947620546	1.168375703	-2.551746189
6	-2.200728706	1.702231133	-3.650863793
1	-2.417043665	2.762410509	-3.809684829
1	-1.123223908	1.567850550	-3.503306829
1	-2.519499308	1.128242831	-4.522733413
1	-2.199723386	0.085573756	0.993644376
9	-10.393206591	-5.069959649	2.331096607

$FHPO_2$

9	5.134771675	-3.088643727	-1.920557833
6	4.006302319	-3.189601205	-1.209530934
6	3.926078460	-4.103306827	-0.162768624
9	4.972139726	-4.875522788	0.139359541
6	2.748287880	-4.197540494	0.574589288
9	2.671474606	-5.052607758	1.601292731
6	1.668113694	-3.380788251	0.258903452
6	1.699182511	-2.456828375	-0.802156857
6	2.907565823	-2.392858826	-1.521193203
9	3.038632794	-1.555987840	-2.558434191
6	0.542848099	-1.590880471	-1.122981328
6	0.773942936	-0.188143214	-1.434542249
6	1.722433067	0.680474105	-0.901228321
7	2.491215085	0.603871544	0.282146272

6	3.874280379	0.366259036	0.249876965
6	4.723816439	1.031619791	-0.618642320
6	6.163381342	0.640044275	-0.636856786
6	6.679320852	-0.091467403	-1.710069407
9	5.871478493	-0.448289209	-2.718631686
6	8.017905131	-0.466466389	-1.770122044
9	8.482672672	-1.170227754	-2.808081781
6	8.876115004	-0.107708182	-0.733244930
9	10.161636828	-0.466367608	-0.775348727
6	8.390450571	0.616734488	0.352831696
6	7.048579149	0.983465688	0.386700745
9	6.609429767	1.687047752	1.441451140
9	9.214496813	0.958298960	1.350218691
6	4.270840772	2.129052352	-1.421878810
6	5.215066803	3.186466428	-1.945200956
8	6.422760323	3.245019440	-1.848691546
7	4.419435209	4.133250348	-2.592678912
6	3.069734843	3.794454976	-2.573909693
6	3.004627170	2.517200641	-1.810250560
7	1.822695561	1.798053390	-1.704427044
6	0.856203714	1.698333726	-2.719197440
6	0.194645137	0.522623655	-2.559631602
1	-0.591850346	0.146705386	-3.198251903
1	0.783073667	2.475555058	-3.461927983
8	2.163885756	4.422406354	-3.089700203
1	4.789712786	4.949877705	-3.059150493
6	4.174699289	-0.461429263	1.384936703
1	5.165769376	-0.803793841	1.647702587
6	3.034270854	-0.629936640	2.119271976
1	2.959774588	-1.125210064	3.076322718
6	1.951735517	0.074153464	1.480496869
6	0.665255104	0.312505231	1.918720620
6	0.129075729	-0.464190244	3.079328736
6	-0.925024909	-1.372325610	2.948202757
9	-1.484031673	-1.573641019	1.739476718
6	-1.454399578	-2.071379609	4.028437951
9	-2.464656531	-2.927381389	3.854344161
6	-0.914764448	-1.877549307	5.297718378
6	0.136855405	-0.981251685	5.470397223
6	0.635692744	-0.284178332	4.372593349
9	1.645163203	0.573838295	4.585278237
9	0.654891803	-0.786660028	6.687153522
9	-1.404924310	-2.545049022	6.343325684
6	-0.138960882	1.386370472	1.366569395
6	0.239194669	2.722384277	1.097048568
1	1.242649095	3.110170054	1.199267129
6	-0.908236866	3.435723210	0.761314407

6	-1.995671781	2.534436997	0.760617560
7	-1.494299728	1.317814300	1.178076765
1	-2.042753517	0.467841493	1.105543588
6	-3.349675359	2.720947212	0.335056319
6	-4.126399148	1.653926452	-0.126106153
7	-3.568033742	0.427897701	-0.392704063
6	-4.560950229	-0.388985001	-0.796530422
6	-4.306498915	-1.741365648	-1.186823975
6	-2.997880340	-2.222323123	-1.315660966
6	-2.496317931	-3.568969290	-1.483258487
6	-1.134403942	-3.531886898	-1.394484034
1	-0.455586328	-4.368477896	-1.483123462
6	-0.722028870	-2.154013822	-1.232460470
7	-1.887883604	-1.417825471	-1.232871762
1	-2.009244421	-0.437048000	-0.972477164
1	-3.112142171	-4.444549787	-1.630959048
6	-5.446880480	-2.668162438	-1.425366363
6	-5.700940330	-3.762603400	-0.589265463
9	-4.902321879	-4.004199818	0.460982521
6	-6.766387443	-4.633033791	-0.806493200
6	-7.626591679	-4.413533211	-1.879202716
6	-7.409240908	-3.330724294	-2.727713135
6	-6.328970640	-2.482971631	-2.497413794
9	-6.147522958	-1.457355403	-3.342411227
9	-8.228828356	-3.119752300	-3.763704740
9	-8.654615113	-5.237947247	-2.094132083
9	-6.974173176	-5.668784609	0.014356726
6	-5.835722727	0.323612883	-0.768571215
1	-6.802761288	-0.083716895	-1.030423237
6	-5.559218936	1.598722798	-0.377658681
1	-6.262540163	2.408864189	-0.237641248
6	-3.896944157	4.098225614	0.353257979
6	-4.459087720	4.691506522	-0.788142963
9	-4.500763752	4.023902283	-1.947778277
6	-4.968434316	5.987242737	-0.774509214
9	-5.488091799	6.521572479	-1.884533282
6	-4.921837799	6.735284680	0.399243369
6	-4.367263963	6.180949127	1.550614331
6	-3.865980060	4.883351605	1.516957584
9	-3.364915299	4.382247834	2.653554447
9	-4.332078349	6.891958347	2.682593265
9	-5.407472968	7.977503401	0.421516055
1	-0.966657775	4.483622743	0.502510293
9	0.581692730	-3.477807036	1.043514860

FHPMO

9	-5.484500000	6.434142000	-2.396964000
6	-5.014705000	5.942650000	-1.245122000
6	-5.021951000	6.733568000	-0.099120000
9	-5.509570000	7.974861000	-0.144598000
6	-4.518932000	6.223338000	1.095472000
9	-4.534719000	6.976070000	2.200868000
6	-4.014897000	4.926740000	1.132053000
6	-3.992943000	4.099702000	-0.001844000
6	-4.503457000	4.648636000	-1.187870000
9	-4.493105000	3.938146000	-2.323249000
6	-3.439836000	2.724673000	0.055923000
6	-4.198077000	1.637032000	-0.361740000
6	-5.613128000	1.564537000	-0.700349000
1	-6.316240000	2.386662000	-0.684436000
6	-5.873263000	0.262155000	-0.998211000
1	-6.820352000	-0.164619000	-1.300757000
6	-4.605751000	-0.456209000	-0.869800000
7	-3.635431000	0.383612000	-0.484613000
6	-4.336356000	-1.842664000	-1.128799000
6	-5.458135000	-2.795665000	-1.293770000
6	-6.480579000	-2.908383000	-0.338613000
9	-6.441632000	-2.164538000	0.776486000
6	-7.552496000	-3.782955000	-0.491630000
9	-8.502823000	-3.861062000	0.447146000
6	-7.623635000	-4.596394000	-1.619850000
9	-8.643051000	-5.445494000	-1.772777000
6	-6.624052000	-4.520549000	-2.586247000
6	-5.569539000	-3.627282000	-2.418932000
9	-4.642162000	-3.576419000	-3.387712000
9	-6.691219000	-5.292759000	-3.677213000
6	-3.020319000	-2.310691000	-1.189606000
7	-1.910519000	-1.501290000	-1.213954000
1	-2.019865000	-0.497145000	-1.083627000
6	-0.740880000	-2.232801000	-1.088988000
6	-1.161601000	-3.623116000	-1.051963000
6	-2.517676000	-3.670218000	-1.150129000
1	-3.138411000	-4.554823000	-1.158819000
1	-0.484923000	-4.464466000	-1.002263000
6	0.529292000	-1.700846000	-1.043176000
6	0.837307000	-0.333055000	-1.479277000
6	0.401342000	0.259358000	-2.712809000
1	-0.308598000	-0.175336000	-3.402230000
6	1.116587000	1.409387000	-2.916504000
1	1.150168000	2.076456000	-3.761945000
7	1.944108000	1.609137000	-1.828658000
6	1.766923000	0.564700000	-0.947749000
7	2.464755000	0.540253000	0.271204000

6	1.864224000	0.166633000	1.499886000
6	0.573413000	0.454723000	1.874396000
6	-0.232405000	1.478782000	1.218581000
7	-1.581693000	1.377047000	0.994360000
6	-2.087660000	2.570092000	0.522365000
6	-1.018891000	3.486325000	0.518640000
1	-1.086533000	4.523324000	0.220484000
6	0.131397000	2.806145000	0.923259000
1	1.127972000	3.206156000	1.019531000
1	-2.123456000	0.520213000	0.934129000
6	0.010098000	-0.178312000	3.109187000
6	-1.028341000	-1.111574000	3.047068000
9	-1.527933000	-1.472701000	1.850837000
6	-1.597782000	-1.676126000	4.184067000
9	-2.588622000	-2.565797000	4.077670000
6	-1.121115000	-1.310246000	5.440717000
9	-1.651337000	-1.847252000	6.540818000
6	-0.089942000	-0.380275000	5.543683000
6	0.451579000	0.178324000	4.388352000
9	1.439719000	1.075743000	4.532905000
9	0.368014000	-0.021299000	6.747526000
6	2.936608000	-0.388687000	2.299937000
1	2.813368000	-0.766410000	3.304094000
6	4.112162000	-0.217354000	1.641645000
6	3.853174000	0.409756000	0.361585000
6	4.761025000	0.862568000	-0.581059000
6	4.415533000	1.734178000	-1.682492000
6	3.085031000	2.534588000	-1.750307000
8	2.835786000	3.348722000	-0.627267000
6	3.851999000	4.301470000	-0.299818000
1	3.454534000	4.870059000	0.542994000
1	4.046645000	4.984074000	-1.135207000
1	4.785309000	3.813561000	-0.005997000
7	3.227815000	3.273557000	-2.969064000
1	2.659264000	4.086324000	-3.163752000
6	4.482685000	3.102433000	-3.567923000
8	4.876993000	3.703558000	-4.548529000
6	5.169691000	2.079834000	-2.754336000
6	6.191256000	0.475999000	-0.429655000
6	7.211531000	1.430573000	-0.297589000
9	6.914156000	2.740256000	-0.271234000
6	8.553263000	1.082006000	-0.177112000
6	8.918990000	-0.262176000	-0.171068000
6	7.936525000	-1.241367000	-0.288282000
6	6.602727000	-0.863900000	-0.419706000
9	5.692975000	-1.844165000	-0.534678000
9	8.276674000	-2.533596000	-0.283328000

9	10.201065000	-0.607765000	-0.046120000
9	9.488377000	2.028209000	-0.047639000
1	5.100642000	-0.440589000	2.017750000
6	1.650260000	-2.613570000	-0.701956000
6	1.693291000	-3.325784000	0.505894000
9	0.733803000	-3.150208000	1.430863000
6	2.718177000	-4.216428000	0.810886000
9	2.727333000	-4.862766000	1.983792000
6	3.747736000	-4.420697000	-0.103413000
6	3.740109000	-3.732237000	-1.313560000
6	2.712484000	-2.837539000	-1.592334000
9	2.740197000	-2.209291000	-2.774435000
9	4.724913000	-3.927330000	-2.195652000
9	4.737674000	-5.270334000	0.179632000
9	-3.563441000	4.468934000	2.306663000
1	6.149450000	1.707027000	-3.020319000

D-a

9	12.986563000	-5.834784000	-1.776647000
6	11.666324000	-6.004244000	-1.914193000
6	11.174108000	-7.153345000	-2.527196000
6	9.798982000	-7.327722000	-2.662101000
6	8.932111000	-6.346341000	-2.188995000
6	9.389214000	-5.180373000	-1.561273000
6	10.777651000	-5.044473000	-1.435404000
9	11.294973000	-3.958659000	-0.840885000
6	8.442966000	-4.154126000	-1.046162000
6	8.437428000	-2.878393000	-1.612173000
6	9.347365000	-2.260623000	-2.558399000
1	10.162399000	-2.769375000	-3.052859000
6	9.011371000	-0.946484000	-2.680129000
1	9.489982000	-0.210311000	-3.310623000
6	7.832600000	-0.692601000	-1.870763000
7	7.521475000	-1.905510000	-1.288498000
1	6.860358000	-2.080788000	-0.531003000
6	7.097608000	0.465892000	-1.703647000
6	7.674280000	1.749681000	-2.168147000
6	8.946559000	2.184840000	-1.760565000
9	9.667011000	1.455264000	-0.891637000
6	9.504759000	3.381199000	-2.199224000
9	10.715077000	3.760677000	-1.770213000
6	8.789601000	4.197569000	-3.071025000
9	9.317094000	5.346795000	-3.500300000
6	7.521959000	3.806175000	-3.494413000
6	6.981166000	2.606312000	-3.041115000

9	5.770827000	2.263769000	-3.500308000
9	6.842485000	4.572902000	-4.352338000
6	5.718320000	0.443376000	-1.215546000
6	4.700711000	-0.498208000	-1.630073000
1	4.867879000	-1.362333000	-2.257246000
6	3.490791000	-0.055930000	-1.189067000
7	3.692198000	1.126256000	-0.477405000
6	5.052343000	1.398251000	-0.453992000
7	5.551686000	2.428238000	0.360341000
6	6.766094000	2.296481000	1.083765000
6	7.373666000	3.611148000	1.066376000
1	8.315390000	3.852648000	1.537172000
6	6.504175000	4.494296000	0.504169000
1	6.625320000	5.567124000	0.443616000
6	5.302088000	3.790188000	0.117802000
6	4.061433000	4.297893000	-0.200888000
6	3.962389000	5.761791000	-0.472576000
6	4.447344000	6.303257000	-1.668797000
9	5.007197000	5.502673000	-2.586508000
6	4.367979000	7.664738000	-1.952760000
9	4.840581000	8.146966000	-3.105918000
6	3.786605000	8.526126000	-1.025395000
9	3.702996000	9.832004000	-1.285409000
6	3.292790000	8.020606000	0.175252000
6	3.391520000	6.656848000	0.438965000
9	2.914039000	6.207236000	1.609039000
9	2.740408000	8.846388000	1.070478000
6	2.848006000	3.517437000	-0.173785000
6	2.680494000	2.033796000	-0.193212000
7	1.439708000	1.639171000	-0.119499000
6	0.691650000	2.814088000	-0.051249000
6	1.548880000	3.971236000	-0.107282000
1	1.222856000	5.000391000	-0.074402000
6	-0.691563000	2.814094000	0.051184000
6	-1.548781000	3.971249000	0.107248000
1	-1.222745000	5.000401000	0.074410000
6	-2.847912000	3.517462000	0.173732000
6	-2.680416000	2.033819000	0.193126000
7	-1.439635000	1.639183000	0.119403000
7	-3.692129000	1.126274000	0.477290000
6	-3.490724000	-0.055940000	1.188907000
1	-2.483544000	-0.400601000	1.359260000
6	-4.700638000	-0.498206000	1.629940000
1	-4.867800000	-1.362346000	2.257093000
6	-5.718241000	0.443420000	1.215486000
6	-5.052270000	1.398295000	0.453926000
7	-5.551626000	2.428301000	-0.360375000

6	-5.301995000	3.790246000	-0.117844000
6	-4.061326000	4.297932000	0.200831000
6	-3.962256000	5.761832000	0.472491000
6	-4.447267000	6.303350000	1.668666000
6	-4.367881000	7.664838000	1.952589000
9	-4.840541000	8.147116000	3.105703000
6	-3.786421000	8.526179000	1.025235000
9	-3.702787000	9.832063000	1.285216000
6	-3.292543000	8.020607000	-0.175363000
9	-2.740079000	8.846345000	-1.070579000
6	-3.391301000	6.656844000	-0.439042000
9	-2.913761000	6.207182000	-1.609071000
9	-5.007202000	5.502813000	2.586368000
6	-6.504073000	4.494378000	-0.504193000
1	-6.625196000	5.567208000	-0.443637000
6	-7.373598000	3.611246000	-1.066374000
6	-6.766049000	2.296568000	-1.083777000
6	-7.128709000	1.174194000	-1.786767000
6	-6.164589000	0.121648000	-2.100879000
6	-4.869800000	0.255089000	-2.639957000
1	-4.367042000	1.195220000	-2.817095000
6	-4.399817000	-1.022551000	-2.952660000
1	-3.439011000	-1.271066000	-3.381228000
6	-5.385446000	-1.951939000	-2.568824000
6	-5.354279000	-3.389522000	-2.555378000
6	-4.414364000	-4.066564000	-3.480718000
6	-3.455386000	-4.984815000	-3.025472000
9	-3.353918000	-5.268406000	-1.721103000
6	-2.564358000	-5.614552000	-3.890624000
9	-1.657974000	-6.476749000	-3.418434000
6	-2.609687000	-5.333013000	-5.253619000
9	-1.760452000	-5.932316000	-6.090097000
6	-3.546480000	-4.424518000	-5.741012000
6	-4.429225000	-3.806996000	-4.860363000
9	-5.329884000	-2.960842000	-5.376324000
9	-3.601321000	-4.161820000	-7.051202000
6	-6.153469000	-4.131308000	-1.690157000
7	-6.871853000	-3.520076000	-0.684853000
6	-7.582549000	-4.471315000	-0.059897000
6	-8.443051000	-4.154007000	1.046178000
6	-8.437452000	-2.878276000	1.612188000
7	-7.521482000	-1.905423000	1.288472000
1	-6.860414000	-2.080713000	0.530938000
6	-7.832535000	-0.692508000	1.870769000
6	-7.097505000	0.465963000	1.703648000
6	-7.674130000	1.749753000	2.168199000
6	-6.980952000	2.606368000	3.041133000

9	-5.770584000	2.263810000	3.500242000
6	-7.521704000	3.806230000	3.494481000
9	-6.842163000	4.572943000	4.352366000
6	-8.789375000	4.197635000	3.071190000
9	-9.316836000	5.346854000	3.500522000
6	-9.504604000	3.381271000	2.199441000
6	-8.946439000	2.184916000	1.760723000
9	-9.666972000	1.455334000	0.891869000
9	-10.714959000	3.760749000	1.770535000
6	-9.011289000	-0.946350000	2.680168000
1	-9.489850000	-0.210162000	3.310682000
6	-9.347335000	-2.260476000	2.558447000
1	-10.162369000	-2.769201000	3.052936000
6	-9.389320000	-5.180226000	1.561308000
6	-8.932237000	-6.346191000	2.189050000
9	-7.615215000	-6.546856000	2.346589000
6	-9.799125000	-7.327549000	2.662173000
9	-9.321094000	-8.426706000	3.256788000
6	-11.174248000	-7.153148000	2.527270000
6	-11.666444000	-6.004051000	1.914245000
6	-10.777754000	-5.044305000	1.435436000
9	-11.295058000	-3.958495000	0.840894000
9	-12.986680000	-5.834571000	1.776698000
9	-12.016571000	-8.083165000	2.984637000
6	-7.338676000	-5.770624000	-0.683889000
6	-6.433071000	-5.559767000	-1.679321000
1	-6.026325000	-6.294405000	-2.361584000
1	-7.780655000	-6.713897000	-0.393115000
7	-6.453249000	-1.216791000	-2.095070000
1	-7.233520000	-1.661955000	-1.624066000
6	-8.488690000	1.092713000	-2.403670000
6	-8.859861000	1.916058000	-3.473757000
9	-8.001984000	2.834672000	-3.944773000
6	-10.111794000	1.830739000	-4.078214000
6	-11.030484000	0.885771000	-3.629067000
6	-10.690470000	0.038334000	-2.577182000
6	-9.437807000	0.156522000	-1.983452000
9	-9.139336000	-0.689580000	-0.979835000
9	-11.565901000	-0.871736000	-2.141967000
9	-12.230078000	0.790881000	-4.205021000
9	-10.430339000	2.642502000	-5.091807000
1	-8.315327000	3.852767000	-1.537146000
6	7.128714000	1.174105000	1.786768000
6	8.488672000	1.092629000	2.403727000
6	9.437778000	0.156374000	1.983627000
6	10.690417000	0.038209000	2.577411000
9	11.565839000	-0.871926000	2.142315000

6	11.030418000	0.885738000	3.629227000
9	12.229990000	0.790872000	4.205229000
6	10.111738000	1.830774000	4.078252000
9	10.430274000	2.642629000	5.091775000
6	8.859829000	1.916069000	3.473744000
9	8.001963000	2.834760000	3.944630000
9	9.139314000	-0.689822000	0.980087000
6	6.164565000	0.121582000	2.100873000
7	6.453194000	-1.216864000	2.095067000
1	7.233474000	-1.662046000	1.624097000
6	5.385365000	-1.951986000	2.568805000
6	5.354154000	-3.389567000	2.555348000
6	6.153331000	-4.131375000	1.690132000
7	6.871746000	-3.520159000	0.684843000
6	7.582425000	-4.471414000	0.059889000
6	7.338487000	-5.770722000	0.683857000
1	7.780433000	-6.714008000	0.393076000
6	6.432872000	-5.559845000	1.679277000
1	6.026086000	-6.294477000	2.361522000
6	4.414200000	-4.066588000	3.480665000
6	4.429036000	-3.807024000	4.860310000
9	5.329702000	-2.960892000	5.376295000
6	3.546254000	-4.424528000	5.740936000
9	3.601069000	-4.161834000	7.051129000
6	2.609451000	-5.332998000	5.253517000
6	2.564149000	-5.614533000	3.890521000
6	3.455214000	-4.984815000	3.025393000
9	3.353781000	-5.268405000	1.721020000
9	1.657759000	-6.476710000	3.418306000
9	1.760181000	-5.932283000	6.089973000
6	4.399761000	-1.022573000	2.952644000
6	4.869770000	0.255053000	2.639930000
1	4.367030000	1.195196000	2.817052000
1	3.438946000	-1.271065000	3.381207000
1	2.483609000	-0.400565000	-1.359462000
9	7.615085000	-6.546984000	-2.346531000
9	9.320931000	-8.426867000	-3.256723000
9	12.016415000	-8.083399000	-2.984518000

MD1-a

9	-13.747761000	0.030443000	5.652925000
6	-12.758409000	0.903168000	5.427904000
6	-12.659336000	2.055153000	6.203478000
9	-13.544808000	2.287869000	7.176048000
6	-11.631689000	2.963178000	5.960853000
9	-11.527807000	4.068514000	6.707944000

6	-10.709981000	2.704136000	4.949956000
6	-10.780569000	1.559936000	4.144725000
6	-11.833032000	0.675113000	4.412132000
9	-11.973504000	-0.441562000	3.681644000
6	-9.794868000	1.313664000	3.057580000
6	-8.933833000	0.216849000	3.145801000
6	-8.931352000	-0.904635000	4.065862000
1	-9.584117000	-1.002412000	4.921350000
6	-8.001340000	-1.803840000	3.640702000
1	-7.748916000	-2.745633000	4.107592000
6	-7.325875000	-1.257516000	2.477748000
7	-7.909683000	-0.022614000	2.262788000
1	-7.809664000	0.574907000	1.441508000
6	-6.291963000	-1.776188000	1.722458000
6	-5.973820000	-3.218006000	1.855942000
6	-6.966012000	-4.207765000	1.734121000
9	-8.235117000	-3.868380000	1.453948000
6	-6.693521000	-5.566095000	1.860304000
9	-7.672878000	-6.468330000	1.722179000
6	-5.390536000	-5.987487000	2.106015000
9	-5.113824000	-7.288599000	2.226635000
6	-4.375569000	-5.041549000	2.221398000
6	-4.668777000	-3.686021000	2.095545000
9	-3.655688000	-2.822638000	2.236876000
9	-3.123588000	-5.446268000	2.468991000
6	-5.439664000	-0.927303000	0.885272000
6	-4.830256000	-1.251628000	-0.327843000
7	-5.061436000	-2.332083000	-1.194633000
6	-6.359118000	-2.776065000	-1.547093000
6	-7.457509000	-1.963600000	-1.706103000
6	-8.811173000	-2.584083000	-1.862886000
6	-9.807958000	-2.426765000	-0.895521000
6	-11.086456000	-2.955387000	-1.043276000
9	-12.008384000	-2.780005000	-0.093047000
6	-11.398149000	-3.678451000	-2.192342000
9	-12.617091000	-4.198155000	-2.346833000
6	-10.432574000	-3.854700000	-3.179924000
9	-10.728018000	-4.543129000	-4.287368000
6	-9.165243000	-3.302084000	-3.010982000
9	-8.266953000	-3.490208000	-3.990573000
9	-9.548707000	-1.717778000	0.219195000
6	-7.352752000	-0.518901000	-1.876771000
7	-8.238229000	0.389737000	-1.352580000
1	-8.848286000	0.241482000	-0.556302000
6	-7.974294000	1.656185000	-1.830174000
6	-8.645946000	2.835953000	-1.353212000
6	-9.235893000	2.883879000	-0.094582000

7	-9.048812000	1.870559000	0.820795000
6	-9.774436000	2.172499000	1.908950000
6	-10.488634000	3.432932000	1.703360000
1	-11.153031000	3.914530000	2.407928000
6	-10.133059000	3.883619000	0.469366000
1	-10.475260000	4.783787000	-0.023804000
6	-8.660049000	4.023511000	-2.240442000
6	-9.138973000	3.947356000	-3.558020000
9	-9.633973000	2.795185000	-4.028143000
6	-9.147314000	5.047627000	-4.409720000
9	-9.625287000	4.938237000	-5.654190000
6	-8.667314000	6.273992000	-3.955771000
6	-8.182928000	6.387407000	-2.655242000
6	-8.181042000	5.273778000	-1.819252000
9	-7.678747000	5.427811000	-0.587716000
9	-7.709271000	7.560323000	-2.220839000
9	-8.673412000	7.336028000	-4.763699000
6	-6.918840000	1.526020000	-2.752638000
6	-6.516617000	0.189456000	-2.761263000
1	-5.717946000	-0.248464000	-3.339161000
1	-6.480518000	2.336052000	-3.318642000
6	-6.217738000	-4.189451000	-1.828828000
1	-7.027798000	-4.832493000	-2.139792000
6	-4.901662000	-4.524964000	-1.764908000
1	-4.470978000	-5.487933000	-1.999126000
6	-4.133618000	-3.351495000	-1.405324000
6	-2.761502000	-3.188969000	-1.297064000
6	-1.891391000	-4.401017000	-1.304119000
6	-1.697878000	-5.163900000	-2.460406000
9	-2.319651000	-4.817858000	-3.600477000
6	-0.877501000	-6.289156000	-2.480630000
6	-0.203711000	-6.666857000	-1.321306000
6	-0.358209000	-5.919510000	-0.155623000
6	-1.194932000	-4.806137000	-0.159940000
9	-1.308127000	-4.096543000	0.971725000
9	0.295083000	-6.274593000	0.955566000
9	0.592364000	-7.738003000	-1.329081000
9	-0.722375000	-6.997672000	-3.604228000
6	-2.125239000	-1.904262000	-1.229416000
6	-2.812564000	-0.552882000	-1.597946000
7	-3.891241000	-0.294244000	-0.638486000
6	-3.911058000	0.673631000	0.345837000
6	-4.868385000	0.336545000	1.266865000
1	-5.105714000	0.890118000	2.164351000
1	-3.214454000	1.494501000	0.306026000
8	-3.411835000	-0.567246000	-2.872829000
6	-2.534057000	-0.836128000	-3.965557000

1	-3.130061000	-0.679067000	-4.866831000
1	-2.176724000	-1.873402000	-3.948978000
1	-1.683381000	-0.146602000	-3.968411000
7	-1.793145000	0.471886000	-1.476015000
6	-0.694150000	-0.134218000	-1.110670000
6	-0.831827000	-1.585053000	-0.951781000
1	-0.048349000	-2.271854000	-0.656357000
6	0.529715000	0.602964000	-0.889230000
7	1.706764000	0.001881000	-0.453185000
1	1.829693000	-0.979250000	-0.253423000
6	2.686322000	0.941772000	-0.350688000
6	2.166470000	2.190429000	-0.740452000
6	0.800869000	1.948321000	-1.063161000
1	0.081300000	2.672673000	-1.415237000
6	2.882490000	3.447885000	-0.883824000
6	2.088831000	4.709430000	-0.905408000
6	1.759301000	5.371435000	-2.091604000
9	2.174870000	4.882251000	-3.269335000
6	1.008064000	6.544918000	-2.096688000
9	0.710123000	7.156024000	-3.248631000
6	0.562763000	7.079327000	-0.890074000
9	-0.158014000	8.202409000	-0.883226000
6	0.875017000	6.444056000	0.310535000
6	1.629893000	5.274631000	0.290376000
9	1.926809000	4.689397000	1.456678000
9	0.454119000	6.963600000	1.467801000
6	4.233800000	3.504011000	-1.099433000
7	5.062641000	2.353893000	-1.026095000
6	4.995046000	1.511668000	0.105655000
7	3.912245000	0.661057000	0.243634000
6	4.098103000	-0.106912000	1.395822000
1	3.330004000	-0.783880000	1.737970000
6	5.282128000	0.256399000	1.962912000
6	5.849991000	1.336360000	1.190970000
6	6.954358000	2.184244000	1.642895000
6	6.791343000	3.653504000	1.590322000
6	5.587925000	4.288159000	1.951725000
9	4.549833000	3.572430000	2.403954000
6	5.414908000	5.666954000	1.870348000
9	4.246664000	6.220643000	2.217916000
6	6.463286000	6.474374000	1.438638000
6	7.671362000	5.886883000	1.072772000
6	7.816658000	4.505271000	1.139313000
9	8.984987000	3.996061000	0.712915000
9	8.676493000	6.651075000	0.627786000
9	6.308726000	7.798944000	1.365537000
6	8.079461000	1.639445000	2.237236000

7	8.442523000	0.310317000	2.127578000
1	8.091883000	-0.366659000	1.450276000
6	9.625675000	0.050763000	2.777164000
6	10.314351000	-1.162131000	2.680905000
6	9.913266000	-2.161617000	1.730533000
7	9.006756000	-1.902251000	0.773465000
6	8.801792000	-3.070841000	0.072899000
6	7.923099000	-3.123697000	-1.008959000
6	7.553347000	-4.429276000	-1.610087000
6	7.731867000	-4.681257000	-2.979196000
6	7.384913000	-5.897034000	-3.561253000
9	7.582136000	-6.103923000	-4.867721000
6	6.840254000	-6.908068000	-2.772916000
9	6.505496000	-8.077800000	-3.320881000
6	6.646446000	-6.692980000	-1.410819000
9	6.110161000	-7.656386000	-0.653514000
6	6.998086000	-5.468482000	-0.848660000
9	6.765523000	-5.300040000	0.459901000
9	8.273423000	-3.746645000	-3.770152000
6	7.320128000	-1.945707000	-1.558477000
6	6.122651000	-1.788318000	-2.286555000
1	5.491463000	-2.596506000	-2.629674000
6	5.894577000	-0.420616000	-2.447858000
1	5.073052000	0.049945000	-2.969423000
6	6.973562000	0.272227000	-1.860859000
7	7.821391000	-0.674978000	-1.356013000
1	8.607188000	-0.513487000	-0.733692000
6	7.271264000	1.701501000	-1.908417000
6	6.309334000	2.643225000	-1.640192000
6	6.272693000	4.053609000	-1.965748000
6	5.055168000	4.563801000	-1.632055000
1	4.704580000	5.568201000	-1.827530000
1	7.066509000	4.579262000	-2.476589000
6	8.614900000	2.135415000	-2.399447000
6	8.781378000	2.704947000	-3.668057000
9	7.715979000	2.905213000	-4.459186000
6	10.028201000	3.094603000	-4.151419000
9	10.142693000	3.642511000	-5.366049000
6	11.162083000	2.901992000	-3.366576000
6	11.037481000	2.326941000	-2.104246000
6	9.776955000	1.961891000	-1.642112000
9	9.698859000	1.400976000	-0.420142000
9	12.120778000	2.140609000	-1.344208000
9	12.362538000	3.265695000	-3.822132000
6	9.634613000	-4.122387000	0.635590000
1	9.696444000	-5.144085000	0.284888000
6	10.340704000	-3.554922000	1.653476000

1	11.056880000	-4.039445000	2.303843000
6	11.490506000	-1.401927000	3.549574000
6	12.740887000	-1.757637000	3.020217000
9	12.903809000	-1.858281000	1.693487000
6	13.848759000	-2.005873000	3.825523000
9	15.023837000	-2.337249000	3.278189000
6	13.736569000	-1.888598000	5.208729000
6	12.514609000	-1.529922000	5.771709000
6	11.416735000	-1.300017000	4.947273000
9	10.255938000	-0.977365000	5.538720000
9	12.397728000	-1.424664000	7.100760000
9	14.795061000	-2.116777000	5.990305000
6	9.986763000	1.284629000	3.443698000
6	9.082568000	2.242454000	3.092866000
1	9.067727000	3.271591000	3.422946000
1	10.851360000	1.410959000	4.079767000
1	5.696211000	-0.142768000	2.877939000
9	-9.729487000	3.598529000	4.754371000

MD2-a

9	11.360950000	0.113640000	7.612962000
6	11.722125000	0.292428000	6.336983000
6	13.064303000	0.476303000	6.014776000
9	13.993239000	0.462679000	6.973774000
6	13.432052000	0.672342000	4.685814000
9	14.721304000	0.836327000	4.368526000
6	12.455972000	0.675862000	3.693609000
6	11.092181000	0.501959000	3.978090000
6	10.761868000	0.314773000	5.329470000
9	9.479739000	0.155327000	5.693274000
6	10.063900000	0.533927000	2.913236000
6	9.165403000	-0.529513000	2.786351000
6	9.198597000	-1.849835000	3.385131000
1	9.922987000	-2.172957000	4.119102000
6	8.207797000	-2.598308000	2.829220000
1	7.964605000	-3.626116000	3.058778000
6	7.462057000	-1.766439000	1.899983000
7	8.069734000	-0.523225000	1.957019000
1	7.935050000	0.247878000	1.307481000
6	6.365718000	-2.080024000	1.126361000
6	5.968433000	-3.507755000	1.031791000
6	6.839869000	-4.495886000	0.549633000
9	8.058862000	-4.160194000	0.093086000
6	6.489644000	-5.841892000	0.503413000
9	7.347947000	-6.748796000	0.019951000
6	5.232103000	-6.241878000	0.946962000
9	4.884371000	-7.529757000	0.905296000

6	4.339290000	-5.290563000	1.433251000
6	4.705482000	-3.949010000	1.457792000
9	3.819209000	-3.072403000	1.946814000
9	3.132907000	-5.669982000	1.864543000
6	5.486641000	-1.057273000	0.544329000
6	4.942844000	0.063895000	1.258691000
1	5.212879000	0.359416000	2.262669000
6	3.953768000	0.625181000	0.496265000
7	3.889847000	-0.053595000	-0.705241000
6	4.827905000	-1.063699000	-0.687975000
7	4.997501000	-1.889490000	-1.813156000
6	6.270192000	-2.287146000	-2.294744000
6	7.396137000	-1.495456000	-2.297299000
6	8.723572000	-2.102453000	-2.629976000
6	9.745384000	-2.200550000	-1.681467000
9	9.533238000	-1.762145000	-0.427264000
6	11.001462000	-2.717096000	-1.983330000
9	11.947903000	-2.793787000	-1.044039000
6	11.264690000	-3.162454000	-3.276661000
9	12.461380000	-3.666629000	-3.581053000
6	10.273377000	-3.077165000	-4.250799000
6	9.029641000	-2.543086000	-3.922651000
9	8.105204000	-2.471511000	-4.894194000
9	10.521902000	-3.496995000	-5.495531000
6	7.334962000	-0.045347000	-2.155383000
7	8.236521000	0.706760000	-1.449847000
1	8.852999000	0.389031000	-0.706383000
6	8.017875000	2.054121000	-1.656696000
6	8.727466000	3.074788000	-0.936772000
6	9.391824000	2.814190000	0.254120000
7	9.288888000	1.585410000	0.874673000
6	10.015411000	1.640449000	1.998021000
6	10.647037000	2.953479000	2.127227000
1	11.279804000	3.278230000	2.942623000
6	10.240129000	3.687311000	1.055465000
1	10.499441000	4.712265000	0.825961000
6	8.727250000	4.458645000	-1.494745000
6	9.883940000	5.015143000	-2.052008000
9	11.017105000	4.301570000	-2.095010000
6	9.906955000	6.306930000	-2.572744000
9	11.030788000	6.806437000	-3.097394000
6	8.747781000	7.078422000	-2.550450000
6	7.578488000	6.553313000	-2.005819000
6	7.582915000	5.262536000	-1.483638000
9	6.438520000	4.793547000	-0.962149000
9	6.463408000	7.291817000	-1.978221000
9	8.756866000	8.316201000	-3.049529000

6	6.972781000	2.149346000	-2.598194000
6	6.532380000	0.856236000	-2.881929000
1	5.740816000	0.568422000	-3.555023000
1	6.569971000	3.067979000	-2.999038000
6	6.062508000	-3.575983000	-2.917484000
1	6.839402000	-4.153346000	-3.396119000
6	4.729965000	-3.850606000	-2.928135000
1	4.248647000	-4.691759000	-3.407016000
6	4.020766000	-2.779030000	-2.268381000
6	2.649528000	-2.587230000	-2.147379000
6	1.746254000	-3.716081000	-2.508271000
6	1.794046000	-4.947851000	-1.842323000
9	2.680469000	-5.138517000	-0.853192000
6	0.952403000	-6.009266000	-2.164406000
9	1.036809000	-7.165201000	-1.500335000
6	0.013198000	-5.856649000	-3.180951000
9	-0.799926000	-6.863428000	-3.500253000
6	-0.066924000	-4.647234000	-3.867997000
6	0.793702000	-3.606597000	-3.532240000
9	0.691426000	-2.470728000	-4.242293000
9	-0.954313000	-4.498416000	-4.855779000
6	2.045834000	-1.346862000	-1.752202000
6	2.802980000	-0.000861000	-1.691325000
7	1.772284000	0.918243000	-1.254612000
6	0.537104000	0.295813000	-1.201667000
6	0.729867000	-1.097053000	-1.460481000
1	-0.081089000	-1.808566000	-1.409125000
6	-0.665921000	0.932584000	-0.932142000
6	-0.909454000	2.306500000	-0.633164000
1	-0.171332000	3.091150000	-0.525403000
6	-2.277047000	2.437081000	-0.431609000
6	-2.787620000	1.077234000	-0.661771000
7	-1.856492000	0.203577000	-0.949206000
7	-4.144066000	0.752125000	-0.788666000
6	-4.640813000	-0.120207000	-1.752979000
1	-3.963647000	-0.791375000	-2.256111000
6	-5.966357000	0.144805000	-1.930564000
1	-6.620115000	-0.338462000	-2.642680000
6	-6.328351000	1.255956000	-1.081195000
6	-5.180127000	1.552111000	-0.349096000
7	-4.981730000	2.432798000	0.733355000
6	-4.211487000	3.607018000	0.596759000
6	-2.986155000	3.629314000	-0.023339000
6	-2.292421000	4.939747000	-0.187819000
6	-2.240294000	5.558222000	-1.442950000
9	-2.836910000	4.981175000	-2.492522000
6	-1.587294000	6.771829000	-1.640632000

9	-1.555341000	7.339742000	-2.851041000
6	-0.965465000	7.400214000	-0.563202000
9	-0.337524000	8.565120000	-0.740727000
6	-1.002712000	6.813612000	0.699212000
6	-1.658425000	5.595894000	0.871239000
9	-1.676460000	5.058663000	2.100030000
9	-0.406207000	7.416749000	1.734064000
6	-4.869643000	4.617794000	1.389568000
1	-4.514913000	5.633235000	1.501391000
6	-5.911626000	4.047344000	2.054650000
6	-5.981656000	2.641020000	1.716032000
6	-6.744847000	1.643031000	2.271505000
6	-6.391099000	0.233002000	2.125820000
6	-5.139601000	-0.382888000	2.328487000
1	-4.220054000	0.143443000	2.541366000
6	-5.323880000	-1.765024000	2.260683000
1	-4.563669000	-2.525451000	2.373279000
6	-6.681457000	-2.008234000	1.971461000
7	-7.299288000	-0.773553000	1.936517000
1	-8.252216000	-0.667894000	1.606092000
6	-7.362400000	-3.235642000	1.673653000
6	-8.535149000	-3.264451000	0.920371000
7	-8.990779000	-2.141990000	0.265636000
6	-10.152579000	-2.463926000	-0.328336000
6	-10.884643000	-1.506900000	-1.108152000
6	-10.335604000	-0.258363000	-1.411512000
7	-9.032168000	0.082497000	-1.143079000
6	-8.787535000	1.418202000	-1.394139000
6	-7.563834000	2.031281000	-1.188190000
6	-7.478699000	3.508839000	-1.210723000
6	-6.491375000	4.195503000	-1.942461000
9	-5.612048000	3.524607000	-2.697637000
6	-6.382739000	5.583237000	-1.932631000
9	-5.420773000	6.189006000	-2.640425000
6	-7.285074000	6.346045000	-1.196984000
9	-7.192582000	7.679012000	-1.189374000
6	-8.278673000	5.706581000	-0.461172000
6	-8.357011000	4.317780000	-0.466174000
9	-9.298761000	3.760196000	0.313279000
9	-9.133778000	6.429268000	0.273542000
6	-10.029652000	1.941484000	-1.929580000
1	-10.167062000	2.955648000	-2.277701000
6	-10.942644000	0.930369000	-1.977141000
1	-11.956673000	0.990260000	-2.345770000
1	-8.447810000	-0.568326000	-0.616700000
6	-12.265386000	-1.826717000	-1.560961000
6	-12.513788000	-2.845337000	-2.490138000

9	-11.491241000	-3.547374000	-3.001452000
6	-13.799227000	-3.171782000	-2.913885000
9	-13.992572000	-4.152195000	-3.804432000
6	-14.888466000	-2.460252000	-2.417460000
6	-14.680762000	-1.436081000	-1.497631000
6	-13.386252000	-1.139343000	-1.077580000
9	-13.234422000	-0.153077000	-0.181004000
9	-15.724800000	-0.753013000	-1.012831000
9	-16.126152000	-2.758240000	-2.822875000
6	-10.492555000	-3.855237000	-0.040612000
6	-9.474456000	-4.356152000	0.714084000
1	-9.390675000	-5.355208000	1.121221000
1	-11.373116000	-4.383172000	-0.380812000
6	-6.758716000	-4.495907000	2.170469000
6	-6.439163000	-5.557126000	1.309475000
9	-6.660135000	-5.452662000	-0.007080000
6	-5.864225000	-6.738343000	1.771223000
9	-5.564095000	-7.723990000	0.917616000
6	-5.586297000	-6.887198000	3.127380000
9	-5.035424000	-8.016123000	3.578916000
6	-5.886666000	-5.853513000	4.011269000
6	-6.462978000	-4.681690000	3.530207000
9	-6.758251000	-3.725948000	4.420320000
9	-5.632752000	-5.998155000	5.316752000
6	-7.877137000	1.999865000	3.180563000
6	-9.209893000	1.747255000	2.842278000
9	-9.500243000	1.179912000	1.656390000
6	-10.270143000	2.036385000	3.695773000
9	-11.528631000	1.775600000	3.330243000
6	-10.010298000	2.612335000	4.936900000
6	-8.696390000	2.880972000	5.310974000
6	-7.654457000	2.566141000	4.441948000
9	-6.403484000	2.836230000	4.846242000
9	-8.441080000	3.429231000	6.504207000
9	-11.016034000	2.903601000	5.764799000
1	-6.535338000	4.525926000	2.795652000
1	1.866708000	1.893478000	-1.504711000
8	3.405838000	0.399987000	-2.899358000
6	2.560213000	0.419381000	-4.055390000
1	2.198835000	-0.580259000	-4.309951000
1	1.703235000	1.089446000	-3.917248000
1	3.185653000	0.802222000	-4.863793000
1	3.254289000	1.413513000	0.719368000
9	12.860324000	0.842616000	2.426229000

Supplementary References

1. Becke, A. D. Density-functional exchange-energy approximation with correct asymptotic behavior. *Phys. Rev. A* **38**, 3098-3100 (1988).
2. Becke, A. D. Density-functional thermochemistry. I. The effect of the exchange-only gradient correction. *J. Chem. Phys.* **96**, 2155-2160 (1992).
3. Hehre, W. J., Ditchfield, R. & Pople, J. A. Self—Consistent Molecular Orbital Methods. XII. Further Extensions of Gaussian—Type Basis Sets for Use in Molecular Orbital Studies of Organic Molecules. *J. Chem. Phys.* **56**, 2257-2261 (1972).
4. Wang, C.-W., Hui, K. & Chai, J.-D. Short- and long-range corrected hybrid density functionals with the D3 dispersion corrections. *J. Chem. Phys.* **145**, 204101 (2016).
5. Blanqui, F., Jouannaud, J.-P. & Okada, M. Corrigendum to "Inductive-data-type systems" [Theoret. Comput. Sci. 272 (1–2) (2002) 41–68]. *Theoret. Comput. Sci.* **817**, 81-82 (2020).
6. Costa, A. C., et al. DFT: B3LYP/6-311G (d, p) vibrational analysis of bis-(diethyldithiocarbamate)zinc (II) and natural bond orbitals. *Spectrochim Acta, Part A* **105**, 251-258 (2013).
7. Boese, A. D. & Martin, J. M. L. Development of density functionals for thermochemical kinetics. *J. Chem. Phys.* **121**, 3405-3416 (2004).
8. Adamo, C. & Barone, V. Toward reliable density functional methods without adjustable parameters: The PBE0 model. *J. Chem. Phys.* **110**, 6158-6170 (1999).
9. Chai, J.-D. & Head-Gordon, M. Long-range corrected hybrid density functionals with damped atom–atom dispersion corrections. *Phys. Chem. Chem. Phys.* **10**, 6615-6620 (2008).
10. Yanai, T., Tew, D. P. & Handy, N. C. A new hybrid exchange–correlation functional using the Coulomb-attenuating method (CAM-B3LYP). *Chem. Phys. Lett.* **393**, 51-57 (2004).
11. Zhao, Y. & Truhlar, D. G. The M06 suite of density functionals for main group thermochemistry, thermochemical kinetics, noncovalent interactions, excited states, and transition elements: two new functionals and systematic testing of four M06-class functionals and 12 other functionals. *Theor. Chem. Acc.* **120**, 215-241 (2008).
12. Tomasi, J., Mennucci, B. & Cammi, R. Quantum Mechanical Continuum Solvation Models. *Chem. Rev.* **105**, 2999-3094 (2005).
13. Wei, P., et al. Neo-Fused Hexaphyrin: A Molecular Puzzle Containing an N-Linked Pentaphyrin. *Angew. Chem. Int. Ed.* **53**, 14069-14073 (2014).
